# Memory of stochastic single-cell apoptotic signaling promotes chemoresistance in neuroblastoma

**DOI:** 10.1126/sciadv.abp8314

**Published:** 2023-03-03

**Authors:** Jordan F. Hastings, Sharissa L. Latham, Alvin Kamili, Madeleine S. Wheatley, Jeremy Z. R. Han, Marie Wong-Erasmus, Monica Phimmachanh, Max Nobis, Chiara Pantarelli, Antonia L. Cadell, Yolande E. I. O’Donnell, King Ho Leong, Sophie Lynn, Fan-Suo Geng, Lujing Cui, Sabrina Yan, Joanna Achinger-Kawecka, Clare Stirzaker, Murray D. Norris, Michelle Haber, Toby N. Trahair, Frank Speleman, Katleen De Preter, Mark J. Cowley, Ozren Bogdanovic, Paul Timpson, Thomas R. Cox, Walter Kolch, Jamie I. Fletcher, Dirk Fey, David R. Croucher

**Affiliations:** ^1^ Cancer Ecosystems Program, Garvan Institute of Medical Research, Sydney, NSW 2010, Australia.; ^2^ School of Clinical Medicine, Faculty of Medicine and Health, UNSW Sydney, Sydney, NSW, Australia.; ^3^ Children’s Cancer Institute, Lowy Cancer Research Centre, UNSW Sydney, Sydney, NSW, Australia.; ^4^ University of New South Wales Centre for Childhood Cancer Research, UNSW Sydney, Sydney, NSW, Australia.; ^5^ Kids Cancer Centre, Sydney Children’s Hospital, Randwick, NSW 2031, Australia.; ^6^ Center for Medical Genetics, Ghent University, Ghent, Belgium.; ^7^ Cancer Research Institute Ghent, Ghent University, Ghent, Belgium.; ^8^ Systems Biology Ireland, School of Medicine, University College Dublin, Belfield, Dublin 4, Ireland.; ^9^ Conway Institute of Biomolecular and Biomedical Research, University College Dublin, Belfield, Dublin 4, Ireland.

## Abstract

Gene expression noise is known to promote stochastic drug resistance through the elevated expression of individual genes in rare cancer cells. However, we now demonstrate that chemoresistant neuroblastoma cells emerge at a much higher frequency when the influence of noise is integrated across multiple components of an apoptotic signaling network. Using a JNK activity biosensor with longitudinal high-content and in vivo intravital imaging, we identify a population of stochastic, JNK-impaired, chemoresistant cells that exist because of noise within this signaling network. Furthermore, we reveal that the memory of this initially random state is retained following chemotherapy treatment across a series of in vitro, in vivo, and patient models. Using matched PDX models established at diagnosis and relapse from individual patients, we show that HDAC inhibitor priming cannot erase the memory of this resistant state within relapsed neuroblastomas but improves response in the first-line setting by restoring drug-induced JNK activity within the chemoresistant population of treatment-naïve tumors.

## Teaser

A stochastic, but targetable form of non-genetic drug resistance emerges from the noise of apoptotic signaling in neuroblastoma.

## INTRODUCTION

The emergence of chemoresistance is a major clinical problem for almost all tumor types where chemotherapy remains the frontline treatment. This includes high-risk neuroblastoma, an aggressive childhood tumor with high rates of chemoresistance and no established, clinically successful targeted therapies beyond disialoganglioside immunotherapy (*1*). These patients receive multiagent chemotherapy, surgery, high-dose consolidation therapy, radiotherapy, and immunotherapy; however, ~15% of these high-risk neuroblastoma patients do not respond to treatment with chemotherapy, and a further 40 to 50% of patients will relapse following an initial response (*2*).

Numerous studies have demonstrated that chemoresistance can arise from the clonal expansion of a low-frequency subset of tumor cells bearing either a preexisting or de novo acquired somatic mutation that promotes therapeutic resistance (*3*). These genetic mechanisms of chemoresistance are known to occur in relapsed high-risk neuroblastoma tumors, occasionally resulting in actionable mutations that are rarely present at diagnosis (*4*, *5*). Emerging precision medicine approaches are now aiming to treat these tumors with targeted therapies (*6*). However, it is becoming apparent that strategies capable of improving the efficacy of first-line treatments, and thereby reducing the occurrence of relapse, are more likely to result in robust improvements in patient outcome (*1*).

An emerging concept in overcoming drug resistance across several tumor types involves recognition of the nongenetic mechanisms that can also play a prominent role in drug response and the acquisition of a resistant state (*7*). To further understand the nature of nongenetic chemoresistance in neuroblastoma, we previously performed patient-specific computational simulations of chemotherapy-induced c-Jun N-terminal kinase (JNK) signaling in neuroblastoma tumors (*8*). This modeling facilitated the identification of poor-outcome tumors hallmarked by an impaired ability to activate apoptotic signaling, revealing that interpatient heterogeneity in JNK activation was due to a positive feedback loop, which amplified the impact of small alterations in the expression of individual components of the JNK network. However, it follows that this nonlinear signaling mechanism would also introduce a form of nongenetic intratumoral heterogeneity in JNK activation, with significant consequences for the dynamics of single-cell apoptotic signaling and the potential to influence patient response to chemotherapy.

Because of the stochastic nature of gene expression, the abundance of any particular protein can vary significantly between individual cells of an otherwise clonal cell population (*9*). This single-cell variation in gene expression emerges from discrete transcriptional bursts (*10*), with evidence to suggest that promoter elements influence the amplitude of these bursts, while enhancers control their frequency (*11*). The dynamics of these transcriptional bursts can also be further influenced by chromatin remodeling (*12*), where the degree of noise observed for an individual gene is influenced by the local accessibility of DNA regions for interaction with the transcriptional machinery. Collectively, these mechanisms can generate a broad distribution of single-cell protein levels that contribute to a significant yet underappreciated source of intratumor heterogeneity (*3*, *13*).

A number of studies have elegantly demonstrated how transcriptional bursts and gene expression noise can result in a rare population of cells with high levels of specific genes that promote therapeutic resistance (*13*–*15*). Here, we have adapted our patient-level model of apoptotic JNK activation to perform predictive simulations of single-cell JNK activity and demonstrate that a chemoresistant phenotype can arise at a much higher frequency within a clonal population as the emergent property of noise within multiple proteins of a functional network, without the need for rare expression states. Through longitudinal high-content imaging and in vivo intravital imaging with a JNK activity biosensor, we now experimentally confirm the existence of these chemoresistant cells and reveal that a memory of this resistant state is retained following treatment with standard-of-care chemotherapy. Promisingly, therapeutic interventions aimed at restoring JNK activity and lowering apoptotic thresholds can sensitize the JNK-impaired cells within primary tumors, highlighting an important option for developing more effective first-line treatment strategies.

## RESULTS

### Predicted and observed single-cell JNK activity

**Fig. 1.**
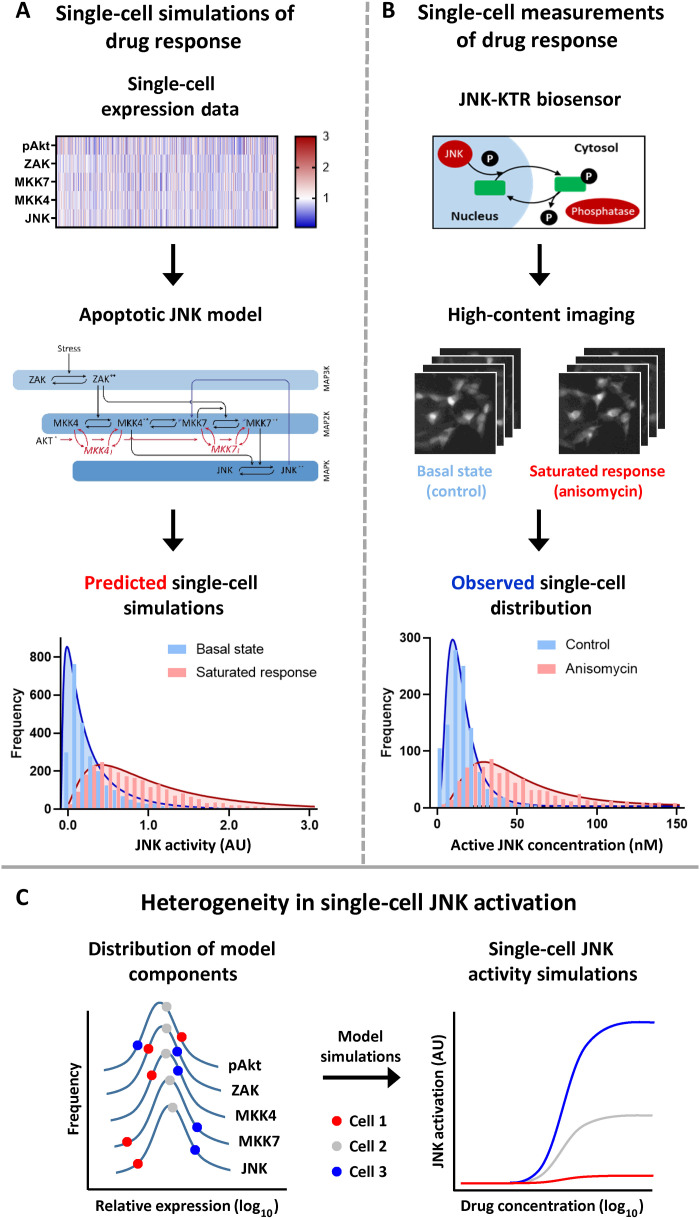
Heterogeneity in single-cell JNK activation. (**A**) Normalized single-cell expression data were generated by five-color flow cytometry in the SH-SY5Y cell line and used as parameters within an ODE model of the apoptotic JNK network 13 to perform single-cell simulations of JNK activation (*n* = 2000). This network model consists of a core three-tiered MAPK cascade (ZAK → MKK4/7 → JNK), with activating phosphorylation shown as black arrows and double-phosphorylated active kinases indicated by asterisks (**). The positive feedback from JNK to MKK7 is shown as a blue arrow, while the inhibitory cross-talk from AKT is shown as a red arrow with the inhibited forms of MKK4 and MKK7 indicated by subscript I. (**B**) SH-SY5Y cells expressing the JNK-KTR mRuby2 biosensor were analyzed by high-content imaging under control conditions and following stimulation with anisomycin (300 nM; 30 min; *n* = 2000). The JNK-KTR cytoplasmic:nuclear ratio was calculated and converted into concentration values of active JNK (see Materials and Methods). (**C**) Schematic demonstrating the integration of gene expression noise within components of the JNK network into a heterogeneous single-cell response. AU, arbitrary units.

To investigate whether the heterogeneity in JNK activation predicted by our patient-specific simulations also translated to the emergence of single-cell heterogeneity, we used five-color flow cytometry to measure the relative single-cell expression of each model component in the SH-SY5Y neuroblastoma cell line, a thrice subcloned derivative of the SK-N-SH line (*16*). This analysis produced single-cell distributions of JNK, mitogen-activated protein kinase (MAPK) kinase kinase 4 (MKK4), MKK7, mitogen-activated protein kinase kinase kinase 20 [(MAP3K20), ZAK], and phospho-Akt (Ser^473^) expression (Fig. 1A and fig. S1A). These normalized distributions were then used as parameters within our existing ordinary differential equation (ODE) model (Fig. 1A and fig. S1B) to perform single-cell simulations of both basal activity and a saturated stress- or chemotherapy-induced JNK activation (Fig. 1A). Performing these single-cell simulations resulted in an extended, log-normal distribution of chemotherapy-induced JNK activity, including a population of cells (~10%) that were unable to activate JNK signaling above the mean value of the basal activation state (fig. S1C).

To validate these model predictions, we stably expressed the JNK mRuby2 kinase translocation reporter (JNK-KTR) within the SH-SY5Y line (Fig. 1B). This biosensor relies upon JNK-dependent phosphorylation of both a nuclear export and import sequence to induce nucleocytoplasmic shuttling of a fluorescent reporter, resulting in a direct correlation between the cytoplasmic:nuclear ratio of the biosensor and single-cell kinase activity (*17*). We then used high-content imaging to measure single-cell JNK activity under control conditions and in response to a saturating concentration of the potent JNK agonist anisomycin (300 nM) at the peak time point of JNK activation (30 min) (*8*). By converting the cytoplasmic:nuclear ratio values obtained with the biosensor into values for active JNK concentration, we could recapitulate the log-normal single-cell distribution of JNK activity predicted by our modeling approach (Fig. 1B), including the presence of a population of JNK-impaired cells below the average obtained under control conditions (fig. S1D). In our previous study, we identified a positive feedback loop from JNK to MKK7, which was vital for the prognostic significance of our patient-specific simulations (*8*). By performing the single-cell simulations with and without this positive feedback (fig. S1C), we could directly compare the distribution of single-cell responses to that obtained with the JNK-KTR biosensor. Through a cumulative distribution analysis of replicate datasets (fig. S1E), we could again demonstrate that the experimental data were more closely recapitulated by the predictions of the full model (*Z* score = 4.05) rather than the model without the positive feedback from JNK to MKK7 (*Z* score = 39.3; fig. S1F).

Together, this combination of model-based prediction and single-cell imaging activity demonstrates that a broad JNK activation potential can exist within a clonal population of cells. In contrast to the emergence of drug-resistant cells resulting from the high expression of a single gene, this single-cell heterogeneity arises because of the varying position of each network component, within each individual cell, on a different point of an otherwise expected distribution (Fig. 1C). Within these simulations, the contribution of gene expression noise for each individual network component to the emergent heterogeneity of single-cell responses becomes apparent when comparing the noise within the distribution of the model components to the noise of the model output (fig. S1G). For the single-cell expression data of each model component (ZAK, MKK4, MKK7, JNK, and phospho-Akt), the coefficient of variation (CoV) was between ~33 and 43%, whereas the CoV for the single-cell JNK activity simulations was ~73%. This was closely matched by a CoV of ~90% derived from the single-cell imaging of the JNK-KTR biosensor, which may also be influenced by signaling components not considered within our model and by noise introduced by the biosensor itself. When this single-cell JNK activity was simulated without the positive feedback between JNK and MKK7, the CoV actually increased, although the amplitude of the signaling output was greatly reduced (fig. S1, E and G). This finding suggests that it is the integration of gene expression noise from multiple network components that generates the inherent noise of JNK signaling, whereas the positive feedback serves mainly to regulate the amplitude of single-cell responses.

### Longitudinal single-cell imaging of JNK activity

**Fig. 2.**
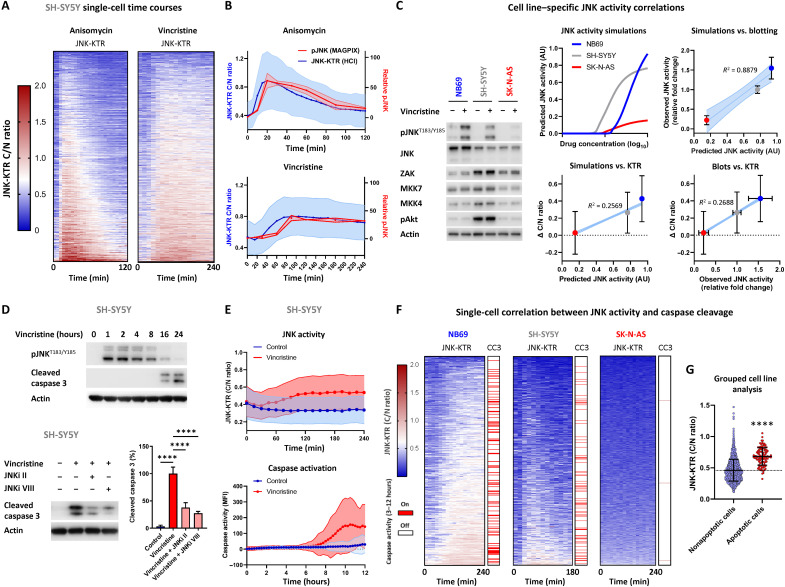
Longitudinal imaging of single-cell JNK activation. (**A**) SH-SY5Y JNK-KTR mRuby2 cells treated with anisomycin (300 nM; *n* = 589) and vincristine (300 nM; *n* = 1080) were imaged every 5 min for 120 min and 15 min for 240 min, respectively. The cytoplasmic:nuclear ratio was calculated for each cell and presented in rows, ranked according to the sum of all time course values. (**B**) The population average of all JNK-KTR single-cell values (blue; means ± SD) correlated to JNK phosphorylation measured by bead-based analysis (red; means ± SD, *n* = 3) at the time points indicated. (**C**) Western blotting for components of the JNK network model, with and without vincristine stimulation (300 nM; 2 hours). Quantified values were used to perform simulations of predicted JNK activation in each cell line, which was correlated to observations of vincristine-induced JNK activity by Western blotting (means ± SD, *n* = 3) and the JNK-KTR (means ± SD, *n* = 589, 654, and 1045). Linear regressions are displayed ± the 95% confidence interval (CI). (**D**) Western blotting of SH-SY5Y cells treated with vincristine and JNK inhibitors as indicated (means ± SD, *n* = 3, ****P < 0.0001). (**E**) Longitudinal high-content imaging of SH-SY5Y JNK-KTR cells in the presence of the fluorescent caspase substrate NucView 488, treated with vincristine (300 nM) and imaged at the time points indicated (*n* = 667 and 883, means ± SD). (**F**) Single-cell tracking of JNK-KTR mRuby2 and caspase activation in NB69 (*n* = 354), SH-SY5Y (*n* = 599), and SK-N-AS (*n* = 1434) cells. All cell lines were treated with vincristine (100 nM) and imaged every 15 min for 12 hours. JNK activity data are presented for the first 3 to 4 hours and caspase activation for the remainder of the time course summarized into an “on” or “off” signal (red line, on). (**G**) The peak JNK activation value for each individual cell across all lines, grouped according to the activation of caspases (means ± SD, ****P < 0.0001). MFI, mean fluorescence intensity.

While our initial high-content imaging analysis was based on a single time point at the peak of JNK activity, we further demonstrated that the single-cell heterogeneity of JNK activation is maintained when individual cells are tracked over an extended time course (Fig. 2A). The treatment of SH-SY5Y JNK-KTR cells with either anisomycin (300 nM; 2 hours) or the neuroblastoma standard-of-care chemotherapy drug vincristine (300 nM; 4 hours) resulted in a broad distribution of single-cell responses, with no delayed activation apparent within cells that had low JNK activity at the peak time point. Furthermore, the averaged population-level dynamics of JNK activity observed with this biosensor (blue) tracked closely to an orthogonal bead-based readout (red) that relies upon antibody-based detection in cell lysates (Fig. 2B). This combined analysis demonstrated that the single-cell imaging approach produced a robust readout of JNK activity and highlights how population-level analyses of signaling activity will inevitably misrepresent the actual distribution of single-cell responses.

In addition to being apparent across multiple stimuli, this heterogeneity in single-cell JNK activation could also be reproduced across multiple cell lines, in proportion to their individual ability to activate this signaling pathway (Fig. 2C and fig. S2, A and B). We have previously demonstrated that alterations in the relative expression level of each component of our JNK activation model can be used to predict the ability of cell lines to activate JNK signaling in response to stress or chemotherapy (*8*). Using the NB69 (high JNK activating), SH-SY5Y (moderate JNK activating), and SK-N-AS (low JNK activating) neuroblastoma cell lines (Fig. 2C), we can again demonstrate this function of the model by using the relative expression levels of JNK, MKK4, MKK7, ZAK, and phospho-Akt in these cell lines as parameters within our model. Using this approach, we can demonstrate a highly significant linear correlation (*R*^2^ = 0.8879) between the predicted ability of each cell line to activate JNK and the observed JNK phosphorylation measured by Western blotting in response to a saturating concentration of vincristine, at the peak time point of activation (300 nM; 2 hours). These model-based predictions also correlate directly to the peak vincristine-induced activation measured using the JNK-KTR in each cell line, albeit with a lower *R*^2^ value influenced by the noise of these single-cell data (fig. S2, A and B). Similarly, the JNK-KTR measurements also correlated directly with the Western blotting measurements. As a whole, these multimodal multiple-cell line data demonstrated the robustness and interrelatability of our model predictions of single-cell JNK activation and the imaging-based readout of JNK activity using the JNK-KTR.

Furthermore, we also demonstrated that this broad distribution of single-cell JNK activity is not an artifact of this particular biosensor or imaging modality, as using an alternative biosensor as a readout of single-cell JNK activity, the JNKAR1 fluorescence resonance energy transfer (FRET) biosensor (fig. S2C) (*18*), recapitulated this single-cell heterogeneity of JNK activation in SH-SY5Y cells following anisomycin stimulation (fig. S2D). In addition, the generation of single-cell clones from the SH-SY5Y JNK-KTR line highlights the stochastic nature of this heterogeneity. Here, the analysis of four individual single-cell clones revealed a similar distribution of JNK activity to that observed within the parental cell line (fig. S2E), confirming that this single-cell heterogeneity was not a result of stable subclonal populations within the SH-SY5Y line.

### Single-cell correlations of JNK activity and apoptosis

As JNK is known to facilitate caspase-dependent apoptosis in response to a number of chemotherapy agents, we next sought to directly correlate drug-induced JNK activity with caspase cleavage at a single-cell resolution. While the treatment of SH-SY5Y cells with vincristine results in a rapid peak of JNK activation after ~2 hours, caspase 3 cleavage is not apparent until between 8 and 24 hours (Fig. 2D, top). Despite this temporal delay, the causative link between JNK activation and caspase cleavage can be demonstrated by the significant reduction in vincristine-induced caspase 3 cleavage caused by the presence of JNK inhibitors (Fig. 2D, bottom). Therefore, we used our longitudinal high-content imaging approach to measure both the JNK-KTR biosensor and caspase activation with a fluorescent caspase substrate. At the population level, the early increase in JNK activity induced by vincristine treatment and the delayed caspase cleavage were readily apparent (Fig. 2E). We therefore correlated these two events at the single-cell level by monitoring JNK activity for the first 3 to 4 hours and then tracking each individual cell for the ensuing 8 hours to determine whether caspase cleavage occurred (Fig. 2F). For clarity, this caspase activation is presented as a simplified “on or off” signal and allows a direct correlation between the early phase of JNK activation and the ultimate initiation of apoptosis at the single-cell level. By ordering the cells vertically according to their levels of JNK activity during the first 3 to 4 hours of imaging, we did not observe a defined threshold, but rather, we found a decreased likelihood of caspase activation in cells with low JNK activity (Fig. 2F). This relationship was conserved across each of the cell lines, although again in proportion to their individual propensity to activate JNK. In the high activating NB69 cells, a population of ~5% of JNK-impaired cells was observed, whereas almost the entire population of cells in the impaired SK-N-AS line did not activate JNK signaling. Rather than a strict threshold, an element of stochasticity in caspase cleavage was still observed within the population of cells with high JNK activity. Still, grouping the single-cell JNK activity data for both apoptotic and nonapoptotic cells from all three lines together revealed the significant elevation in JNK activity required for the initiation of apoptosis, with almost all apoptotic cells having JNK activity above the mean value of the nonapoptotic cells (Fig. 2G). Together, these data suggest that a threshold level of JNK activity is necessary for the induction of apoptosis but is not sufficient in isolation for full commitment to this cell fate decision. In this context, it is likely that further regulation by downstream components is responsible for the initiation of caspase cleavage once the JNK threshold is breached.

### Chemoresistance in JNK-impaired cells

**Fig. 3.**
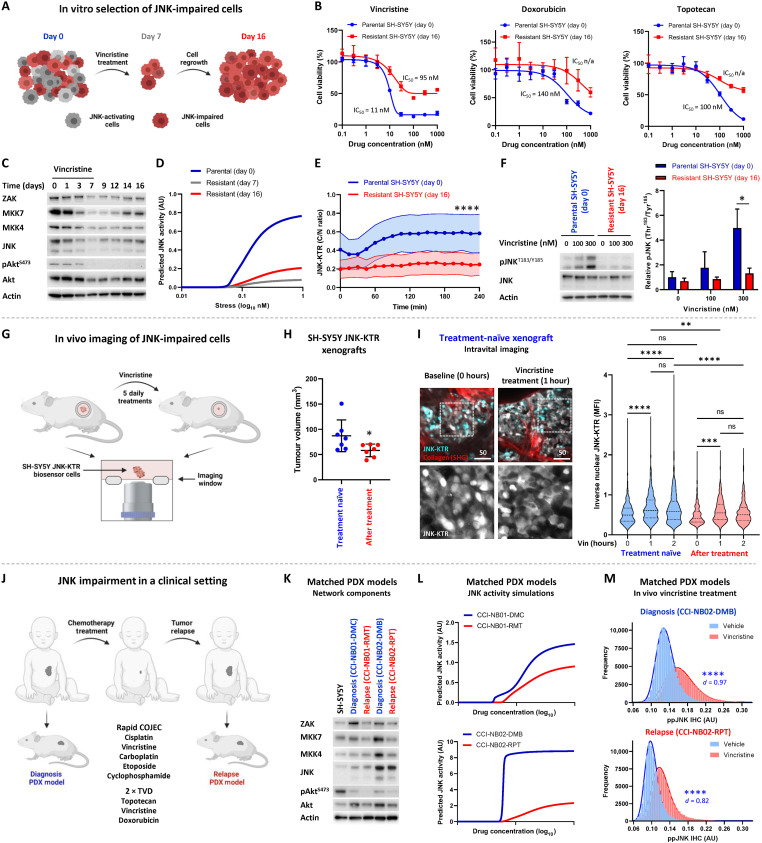
In vitro, in vivo, and clinical observations of JNK-impaired cells. (**A**) Schematic of in vitro selection of JNK-impaired cells through vincristine treatment (100 nM). (**B**) Cytotoxicity assays performed with parental and resistant SH-SY5Y cells with the indicated drugs and concentrations (48 hours, *n* = 6, means ± SD). (**C**) Western blotting of SH-SY5Y cells collected during long-term vincristine treatment (100 nM). (**D**) Predictive simulations of JNK activation using expression levels of the JNK network components during vincristine treatment. (**E**) Longitudinal high-content imaging of parental and resistant SH-SY5Y JNK-KTR cells, treated with vincristine (300 nM) and imaged at the time points indicated (*n* = 231 and 372, means ± SD, ****P < 0.0001 for all time points). (**F**) Western blotting of parental and resistant SH-SY5Y cells following stimulation with vincristine at the concentrations indicated (2 hours, *n* = 3, means ± SD, *P < 0.05). (**G**) Schematic of the in vivo intravital imaging of SH-SY5Y JNK-KTR mClover H2B-mCherry cells through titanium windows, before and following five daily vincristine treatments (1 mg/kg, i.v.). (**H**) Tumor volume of SH-SY5Y JNK-KTR mRuby2 xenografts before and after this treatment regimen (*n* = 7, *P < 0.05). (**I**) Intravital imaging of treatment-naïve SH-SY5Y JNK-KTR mClover H2B-mCherry xenografts before treatment and again 1 and 2 hours following vincristine treatment (*n* = 243 to 435, **P < 0.01 and ****P < 0.0001). Scale bars, 50 μm. (**J**) Schematic for the generation of matched PDX models established at the point of diagnosis and relapse in the same patient. (**K**) Western blotting of tumor lysates from two sets of matched PDX models. (**L**) Predictive simulations of JNK activation using expression levels of the JNK network components from the two matched PDX models. (**M**) Single-cell quantification of cytoplasmic immunohistochemistry staining for pJNK^T183/Y185^ following in vivo vincristine (1 mg/kg, i.v.; 2 hours) or vehicle treatment (*n* = 98,104 and 73,304 for CCI-NB02-DMB; *n* = 75,739 and 73,655 for CCI-NB02-RPT). All schematic images were created with BioRender.com. ns, not significant; IC_50_, median inhibitory concentration; n/a, not available.

The combination of our single-cell modeling and longitudinal imaging suggests that a population of JNK-impaired cells, existing because of gene expression noise in components of the JNK network, may be inherently chemoresistant. We therefore undertook a number of in vitro, in vivo, and clinical approaches to validate this hypothesis. First, we sought to isolate these nonapoptotic, drug-resistant neuroblastoma cells through in vitro drug treatment (Fig. 3A). This selection of resistant cells by culturing in the presence of an IC_95_ concentration of vincristine (100 nM) (*8*) for 7 days resulted in a cell population that demonstrated cross-resistance to two other neuroblastoma standard-of-care chemotherapy drugs, doxorubicin and topotecan (Fig. 3B), indicating a general apoptotic defect in these cells instead of a drug-specific resistance mechanism. Furthermore, whole-genome sequencing of the parental SH-SY5Y cell line and three resistant cell populations (fig. S3A) precluded the presence of any preexisting low-frequency somatic mutation that became enriched following the selection of these resistant populations. Known driver mutations in *KRAS* (G12V) and *ALK* (P1174L) were identified by this analysis, along with a number of other potentially somatic mutations, but the frequency of these mutations was not altered across all the resistant populations (fig. S3B). In addition, analysis of copy number variation identified known features of the SH-SY5Y cell line, including the gain of chromosome 17q and the loss of chromosome 14p and 22q (fig. S3C) (*19*). However, there was no change in copy number variation across all chromosomes between the parental SH-SY5Y line and the resistant populations (Supplementary Materials). We next quantified genomic levels of DNA methylation (5-methylcytosine), a repressive gene-regulatory mark that has previously been shown to control the expression of many apoptosis-related genes in neuroblastoma (*20*). We observed that global DNA methylation patterns were also maintained between the parental SH-SY5Y cells and the resistant populations (fig. S3D). Collectively, this analysis demonstrated that the apoptotic defect within this cell population was the result of a nongenetic mechanism of drug resistance, in line with their proposed existence due to gene expression noise.

In support of this hypothesis, we observed significantly lower expression of each JNK network component within the population of surviving cells present during the course of drug selection, which only partially increased upon the removal of drug and expansion of the cell population (Fig. 3C and fig. S3, E and F). This included a decrease in the inhibitory form of phosphorylated Akt (S473), which may have been influenced by the decreased cell density following drug treatment. Nonetheless, using the relative expression levels of the JNK network components as parameters within our model of JNK activation, we could predict that the cells surviving at the point of selection (day 7) still had a greatly decreased ability to activate JNK signaling in response to vincristine, which only partially recovered in the resulting cell population (day 16; Fig. 3D). The significantly impaired drug-induced JNK activation of this resistant cell population was further confirmed by high-content imaging of the JNK-KTR biosensor following vincristine treatment of parental (day 0) and resistant (day 16) SH-SY5Y lines (Fig. 3E), along with Western blotting of lysates from both the parental and resistant SH-SY5Y populations (Fig. 3F). This finding suggests that vincristine treatment resulted in the selection of a population of cells based on an impaired ability to activate apoptotic JNK signaling, which existed before treatment because of gene expression noise. The preexisting nature of this JNK-impaired population was further supported by a correlation between the relative expression levels of JNK, MKK4, MKK7, and ZAK observed at the point of drug selection (day 7; fig. S3G) and the averaged expression level of these components within the lowest 5% of JNK-activating cells from the single-cell simulations performed in this cell line before treatment (Fig. 1A).

To further validate this process of drug-induced selection on the basis of impaired single-cell JNK activation, we next performed in vivo longitudinal intravital imaging of JNK activity through titanium optical windows in neuroblastoma xenografts during the course of chemotherapy treatment (Fig. 3G). For this imaging, we used SH-SY5Y cells expressing the JNK-KTR Clover biosensor and H2B-mCherry as a nuclear marker (fig. S4A), which demonstrated a broad single-cell distribution of JNK activity in vitro (fig. S4B), similar to the JNK-KTR mRuby2 biosensor (Fig. 2A). Single-cell JNK activity was first quantified before treatment and then again 1 and 2 hours following vincristine treatment [1 mg/kg, intravenous (i.v.)]. This imaging was performed in treatment-naïve tumors and then again in the same tumors following 5 days of vincristine treatment, which caused significant tumor regression within this model (Fig. 3H). In line with our in vitro assay, this intravital single-cell imaging confirmed that a resistant cell population remained following chemotherapy treatment in vivo with a significantly impaired ability to activate JNK signaling (Fig. 3I).

The ability of these resistant cells to maintain their JNK-impaired state, given that their selection was initially based on random gene expression noise, was further supported by our demonstration that this loss of function in JNK signaling also occurs upon relapse in the clinical setting. Here, we used two matched patient-derived xenograft (PDX) models (Fig. 3J) (*21*), each established from individual patients at both diagnosis and relapse as part of the SIOPEN HR NB-1 clinical trial. Both patients received Rapid COJEC induction chemotherapy (cisplatin, vincristine, carboplatin, etoposide, and cyclophosphamide). One patient (CCI-NB01) did not respond completely to induction chemotherapy and received an additional two cycles of topotecan, vincristine, and doxorubicin chemotherapy but developed progressive disease. The other patient (CCI-NB02) achieved a complete response following Rapid COJEC but experienced prolonged treatment delays due to a severe surgical complication and ultimately relapsed. To investigate molecular changes that may have influenced this JNK-impaired state, we performed whole-genome sequencing on these PDX models (fig. S5A and table S1) and identified neuroblastoma driver mutations as previously described (*22*). In both pairs of patient models, the tumor mutation burden of single-nucleotide variants increased (3.0 to 5.0 and 0.72 to 1.97 mutations per megabase), but there was no gain or loss of driver mutations at relapse in either model. In one model, with high-level *MYCN* and *ALK* amplification with *ALK* fusion at diagnosis (CCI-NB02), all three drivers remained intact at relapse. A clonal nonsense driver mutation in *SETD2* was lost at relapse, and several subclonal variants were observed in *ALK* and *MYCN* in <5% of the tumor cells, which is often observed in highly amplified oncogenes. In CCI-NB01, the *MYCN*, *NF-1*, *CDKN2A/B*, and *TERT* driver mutations found at diagnosis were also retained at relapse. Several arm-level copy number alterations were lost at relapse, including a chr4 and chr14 amplification from three copies down to two, which are unlikely to be driver events. In summary, while some genetic changes were observed, the main driver mutations in key neuroblastoma genes were retained between diagnosis and relapse.

Therefore, using lysates prepared from these PDX tumors, we measured the relative expression of all JNK network model components (Fig. 3K) and simulated their ability to activate drug-induced JNK signaling (Fig. 3L). Notably, these simulations predicted that CCI-NB01-DMC (diagnosis) would have a lesser ability to activate JNK than CCI-NB02-DMB (diagnosis), which is in line with the innate resistance observed for the original CCI-NB01 patient tumor. Furthermore, these simulations demonstrated that both models would have a greatly impaired ability to activate JNK signaling upon relapse, when compared to the matched treatment-naïve tumor. Vincristine treatment of the matched CCI-NB02 PDX models in vivo recapitulated this expected pattern of JNK activation. Immunohistochemistry staining of pJNK^T183/Y185^ in these tumors (fig. S5B) followed by single-cell quantification of JNK phosphorylation (Fig. 3M) demonstrated that a broad, log-normal distribution of JNK activity could be observed within the CCI-NB02-DMB (diagnosis) model, similar to the heterogeneity predicted by our model of single-cell JNK activity in SH-SY5Y cells (Fig. 1A). Furthermore, JNK activation was also significantly impaired within the matched relapse model (CCI-NB02-RPT) when compared to the diagnosis model (*P* < 0.0001, *d* = 1.01), as predicted by our simulations (Fig. 3L). These observations were also observed following vincristine treatment performed on ex vivo cultures of all four PDX models (fig. S5C). This orthogonal analysis validated both the innate JNK-impaired state of the CCI-NB01-DMC model and the consistent JNK impairment within both relapse models (fig. S5D).

Together, these combined observations from in vitro, in vivo, and clinical models of drug relapse demonstrate that treatment with chemotherapy can drive tumor evolution toward a JNK-impaired phenotype. This occurs through the selection of an initially stochastic population of JNK-impaired cells, which then demonstrate an intriguing ability to retain this impaired state following cessation of treatment.

### Restoring activity to JNK-impaired cells

To further investigate the hypothesis that these JNK-impaired cells exist because of gene expression noise in components of the JNK network, we also investigated a number of alternative hypotheses by performing high-content imaging of the JNK-KTR biosensor coupled to costaining assays. As large sample sizes, such as those generated with single-cell imaging, can produce statistically significant results despite small differences between samples, for all datasets with *n* > 1000, we adopted a combined approach of not only analyzing *P* values to assess significance but also measuring effect size (*d*, Cohen’s test) to determine the magnitude of differences (see Materials and Methods for more details).

**Fig. 4.**
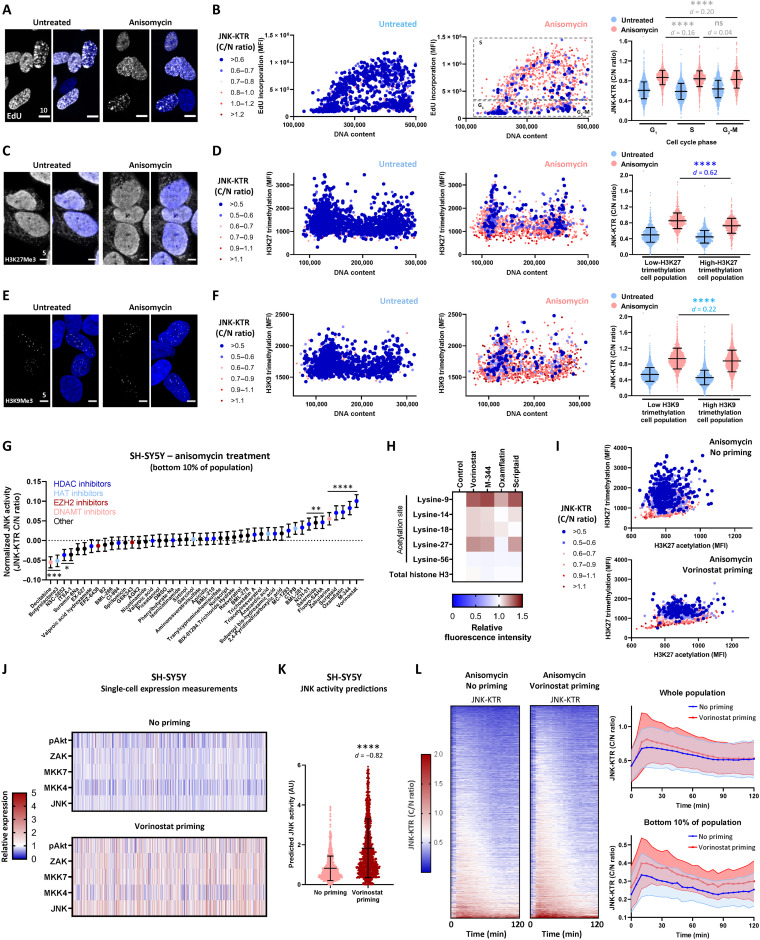
Restoring JNK activity with an HDAC inhibitor. (**A** to **F**) SH-SY5Y JNK-KTR mRuby2 cells were treated with anisomycin (300 nM; 30 min), fixed, and stained as indicated, followed by confocal microscopy and high-content imaging. For visualization, the JNK-KTR cytoplasmic:nuclear ratio values were binned as indicated, with dark blue points indicating the JNK-impaired cells (*n* = 2000). The JNK-KTR data were quantified on the basis of cell cycle stage (B) (untreated *n* = 7399 and anisomycin *n* = 7093), a median H3K27me3 cutoff (D) (untreated *n* = 2443 and anisomycin *n* = 2098), or a median H3K9me3 cutoff (F) (untreated *n* = 6352 and anisomycin *n* = 5551; means ± SD). Scale bars, 5 or 10 μm. (**G**) SH-SY5Y JNK-KTR mRuby2 cells were pretreated as indicated (100 nM; 24 hours), followed by anisomycin treatment (300 nM; 30 min) and high-content imaging. JNK-KTR ratios are displayed for the lowest 10% of cells (*n* = 212 to 300). (**H**) SH-SY5Y cells were pretreated as indicated (100 nM; 24 hours), then fixed, and stained as indicated for high-content imaging. (**I**) SH-SY5Y JNK-KTR mRuby2 cells were treated with vorinostat (100 nM; 24 hours) and then anisomycin (300 nM; 30 min) before fixing and staining with 4′,6-diamidino-2-phenylindole (DAPI) and the antibodies indicated for high-content imaging (*n* = 2000). (**J**) SH-SY5Y cells were treated with vorinostat (100 nM; 24 hours), then fixed, and stained for flow cytometry analysis as indicated. Values were normalized to the mean value of each component in the untreated population (*n* = 1000). (**K**) Predictive simulations of peak single-cell JNK activation using the data in (J) (means ± SD, *n* = 1000). (**L**) Longitudinal single-cell tracking of SH-SY5Y JNK-KTR mRuby2 cells, pretreated with vorinostat (100 nM; 24 hours) before anisomycin stimulation (300 nM). Cells were imaged every 5 min for 2 hours (means ± SD, *n* = 3110 for no priming and *n* = 3664 for vorinostat priming). Negligible effect sizes with *d* < 0.2 are shown in light gray; small effect sizes with *d* = 0.2 to 0.5 are shown in light blue, and large effect sizes with *d* > 0.5 are shown in dark blue; *P < 0.05, **P < 0.01, ***P < 0.001, and ****P < 0.0001.

Within the literature, drug-resistant cell populations are often described as slow cycling or stem cell-like (*7*). Therefore, we first combined 5-ethynyl-2′-deoxyuridine (EdU) incorporation (a marker of S phase cells) and staining for DNA content to determine the cell cycle state of JNK-impaired cells (Fig. 4A). This analysis demonstrated that there was no correlation between cell cycle state and the ability of SH-SY5Y cells to activate JNK signaling, suggesting that they are proliferating at a similar rate to the whole cell population (Fig. 4B). Second, we investigated single-cell expression levels of the common drug-efflux pump ABCB1, which has been implicated in multidrug resistance across a number of tumor types (*23*). Here, we used antibody staining to reveal that there was no correlation between single-cell ABCB1 expression levels and JNK signaling (fig. S6A). We then investigated the potential impact of two main JNK phosphatases, the inducible dual specificity protein phosphatase 16 (DUSP16) (fig. S6B) and the constitutively expressed DUSP1 (fig. S6C). For DUSP1, there was no correlation between elevated phosphatase expression and impaired JNK signaling, although DUSP16 contributed a small effect to the reduced JNK activity in resistant cells, which is likely too small to lend itself to therapeutic targeting.

While the oncogenic transcription factor N-Myc is commonly amplified in high-risk neuroblastoma, the SH-SY5Y cell line instead displays elevated expression of the related c-Myc protein (*24*). We therefore investigated the correlation between single-cell expression levels of c-Myc and JNK signaling (fig. S6D), again demonstrating only a small effect size association between elevated c-Myc and impaired JNK signaling.

The repressive chromatin marks histone-3 lysine-9 trimethylation (H3K9me3) and lysine-27 trimethylation (H3K27me3) have also been previously associated with drug-resistant cells (*25*). These histone modifications are known to promote a closed chromatin state, which can influence gene expression noise by reducing the availability of specific regions of DNA and, thus, the frequency of transcriptional bursts from these sites (*12*). Accordingly, costaining with antibodies against these marks revealed that elevated levels of H3K27me3 were significantly associated with impaired JNK signaling, with a large effect size (Fig. 4, C and D). Elevated H3K9me3 levels were also significantly associated with impaired JNK activation but with a small effect size (Fig. 4, E and F). H3K27me3 is known to be catalyzed by the enhancer of zeste homolog 2 (EZH2) methyltransferase as part of the Polycomb repressive complex 2. In line with this, costaining with an EZH2 antibody revealed that elevated single-cell expression of EZH2 was significantly associated with both higher H3K27me3 and impaired JNK activation, with a large effect size (fig. S7A), adding a further layer to the complex influence of gene expression noise on single-cell JNK signaling.

Given the potential association between a repressed chromatin state and drug resistance, we sought to determine whether an epigenetic focused therapeutic strategy could be developed to restore apoptotic capability to JNK-impaired cells. To achieve this, we primed parental SH-SY5Y cells with a library of 47 drugs (100 nM) targeting a number of different epigenetic regulators for 24 hours before stimulation with anisomycin (Fig. 4G). Unexpectedly, at this concentration, the drugs most effective at restoring JNK activation within the impaired cells (i.e., the lowest 10% of the whole population) were the four histone deacetylase (HDAC) inhibitors—vorinostat, M-344, oxamflatin, and scriptaid—an early HDAC inhibitor from which vorinostat was eventually developed. This was despite the presence of EZH2 inhibitors within the library, including GSK-343 and EPZ-6438 (tazemetostat), which we expected would influence JNK activity through repression of H3K27me3 levels. Further analysis demonstrated that treatment of SH-SY5Y JNK-KTR cells with EPZ-6438 at 100 nM, over an extended time course, resulted in a significant but modest decrease in H3K27me3 and a nonsignificant increase in JNK activity within impaired cells (fig. S7, B and C). However, using EPZ-6438 at the higher concentration of 1 μM resulted in a slow but continual decrease in H3K27me3 over a 96-hour period, with significantly increased JNK activation observed within impaired cells after 48 to 72 hours (fig. S7, D and E).

While EPZ-6438 is known to function by reducing H3K27me3, each of the HDAC inhibitors vorinostat, M-344, and scriptaid significantly increased acetylation at both H3K27 (H3K27acet) and H3K9 (H3K9acet) in SH-SY5Y cells, while oxamflatin only significantly increased H3K9acet (Fig. 4H and fig. S7F). Costaining for H3K27acet and H3K27me3 in anisomycin-stimulated SH-SY5Y JNK-KTR cells demonstrated that baseline single-cell H3K27acet levels did not influence JNK activity, unlike the inverse relationship associated with H3K27me3 (fig. S8A). However, further analysis of costaining data revealed that priming with vorinostat not only elevated H3K27acet but also significantly reduced H3K27me3 (Fig. 4I and fig. S8B), with similar results observed for H3K9acet/Me3 (fig. S8C). While it cannot be excluded that changes in histone acetylation levels alone could be responsible for restoring JNK activity within impaired cells, the lack of influence of baseline H3K27acet levels on JNK activity suggests that promoting a relaxed chromatin state by simultaneously decreasing trimethylation and increasing acetylation may restore apoptotic JNK activation more rapidly than the relatively slower dynamics observed through the inhibition of trimethylation achieved by EZH2 inhibitors.

Vorinostat is a class I/II HDAC inhibitor that has undergone both phase 1 and 2 clinical trials in pediatric populations (*26*). Rather than having a global impact upon gene expression, it has previously been shown to affect the expression of discrete subsets of genes in cancer cells, specifically those associated with cell cycle and apoptosis regulation (*27*). In line with this, the priming of SH-SY5Y cells with vorinostat resulted in increased single-cell expression of all components of the JNK network model (Fig. 4J and fig. S8D), including the inhibitory phospho-Akt. Nonetheless, simulating the single-cell distribution of JNK activation on the basis of these expression profiles predicted that the changes induced by vorinostat priming would be sufficient to significantly increase the ability of the entire cell population to activate apoptotic JNK signaling (Fig. 4K). This prediction was validated by the single-cell tracking of JNK activity in SH-SY5Y cells (Fig. 4L), which demonstrated that vorinostat priming (100 nM; 24 hours) increased anisomycin-induced JNK activity across the whole population of cells, with a particularly prominent effect upon the most impaired bottom 10% of cells.

**Fig. 5.**
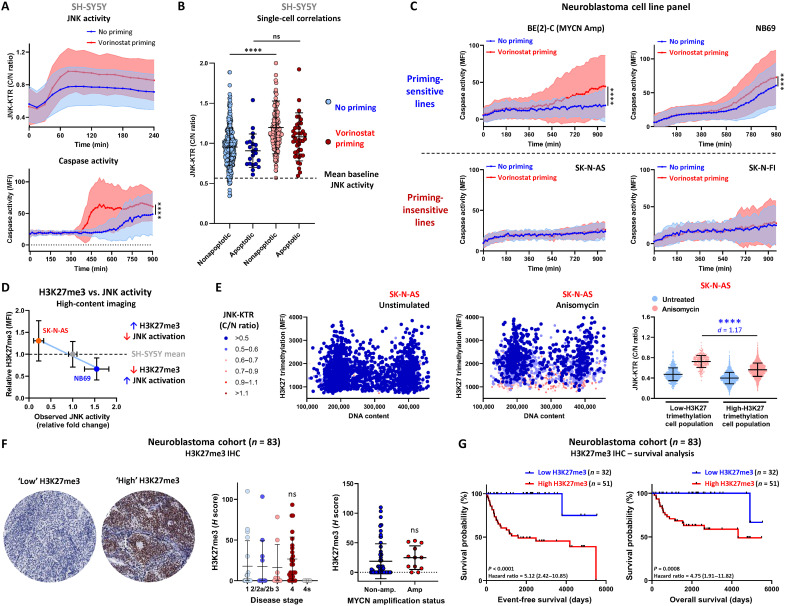
H3K27me3 is elevated in JNK-impaired cells. (**A**) Longitudinal tracking of SH-SY5Y JNK-KTR mRuby2 cells in the presence of NucView 488, pretreated with vorinostat (100 nM; 24 hours) before vincristine stimulation (100 nM). Cells were imaged every 15 min for 15 hours (means ± SD, *n* = 1114 for no priming and *n* = 1210 for vorinostat priming, ****P < 0.0001). (**B**) Peak JNK activity in apoptotic and nonapoptotic cells from the data presented in (A). (**C**) Longitudinal tracking of the cell lines indicated in the presence of NucView 488, pretreated with vorinostat (100 nM; 24 hours) before vincristine stimulation (100 nM). Cells were imaged every 15 min for 16 hours (means ± SD, *n* > 840 in all conditions, ****P < 0.0001). (**D**) Correlation between relative H3K27me3 levels in the NB69, SH-SY5Y, and SK-N-AS lines, obtained by high-content imaging of H3K27me3 antibody staining (means ± SD, *n* = 2000). (**E**) High-content imaging of SK-N-AS JNK-KTR mRuby2 cells stained with an H3K27me3 antibody and DAPI nuclear marker following anisomycin stimulation (300 nM; 30 min). For visualization, the JNK-KTR cytoplasmic:nuclear ratio values are displayed in the colored bins as indicated, with the dark blue points indicating the JNK-impaired cells (*n* = 2000). Raw JNK-KTR cytoplasmic:nuclear ratio values were used for quantification based on a median H3K27me3 cutoff from SH-SY5Y cells (means ± SD, *n* = 2000 for untreated and *n* = 2000 for anisomycin, ****P < 0.0001). (**F**) Immunohistochemistry staining for H3K27me3 in a tumor microarray cohort of 83 pretreatment neuroblastoma patient samples. Two representative images of low and high H3K27me3 are shown, along with the correlation between H3K27me3 staining and disease stage or MYCN amplification status of each tumor. (**G**) Overall and relapse-free survival analysis in this cohort based on H3K27me3 expression. Samples with an *H* score of >0 were considered “high H3K27me3.”

Using the neuroblastoma standard-of-care chemotherapy drug vincristine, we also observed that vorinostat priming of SH-SY5Y JNK-KTR cells significantly increased vincristine-induced JNK and caspase activation (Fig. 5A). In addition, single-cell tracking in this context revealed that vorinostat priming did not alter the threshold required for the initiation of apoptosis; instead, it increased the peak JNK activity of all cells, ensuring that JNK activity within all cells was now above the previously defined threshold of mean baseline activation (Fig. 5B). This priming regimen was also effective at sensitizing the BE(2)-C (MYCN-amplified) and NB69 neuroblastoma cell lines to vincristine treatment, although the SK-N-AS and SK-N-FI lines were not sensitized by vorinostat priming (Fig. 5C), suggesting a context dependency to this sensitization mechanism.

Comparing the levels of H3K27me3 between the SH-SY5Y line, the high JNK-activating priming-sensitive NB69 cell line, and the JNK-impaired priming-insensitive SK-N-AS line revealed an inverse correlation between H3K27me3 levels and JNK activity (Fig. 5D). This pattern of H3K27me3 was recapitulated by an analysis of lysates from these three lines (fig. S9A), although these H3K27me3 levels were independent of EZH2 expression levels. In the single-cell analysis, most cells within the low-H3K27me3 JNK-activating NB69 cell line sit below the SH-SY5Y median, whereas the high-H3K27me3 JNK-impaired SK-N-AS cells are mostly above this cutoff. Accordingly, correlating single-cell H3K27me3 with JNK activity in SK-N-AS cells confirmed that the highly methylated cells were significantly JNK-impaired compared to the much smaller population of cells with low levels of H3K27me3 (Fig. 5E).

While we have not demonstrated a direct causal mechanism, these data suggest that elevated H3K27me3 may be associated with a JNK-impaired drug-resistant phenotype at the single-cell level. As we have previously demonstrated that JNK impairment is associated with poor overall survival in neuroblastoma, we investigated the association between H3K27me3 levels and patient survival in a neuroblastoma cohort. Immunohistochemistry staining of H3K27me3 in this cohort of 83 pretreatment patient samples revealed that the levels of this repressive chromatin mark were highly elevated in a subset of tumors, with a nonsignificant increase in stage 4 tumors and no significant association with MYCN amplification status (Fig. 5F). However, elevated H3K27me3 was significantly associated with both poorer overall and event-free survival (Fig. 5G).

The SH-SY5Y cell line was originally derived from a posttreatment metastasis, which could have contributed to the presence of a cell population with elevated H3K27me3 levels; however, our analysis of patient samples and high-content imaging of a number of cell line models confirms that populations of cells with high levels of H3K27me3 can be present in neuroblastoma tumors before treatment. The association of this state with poor patient survival may also point to a subset of patient tumors that are inherently resistant to standard-of-care chemotherapy due to repressed apoptotic JNK signaling and may require alternative treatment strategies.

### Epigenetic imprinting in relapsed neuroblastoma

**Fig. 6.**
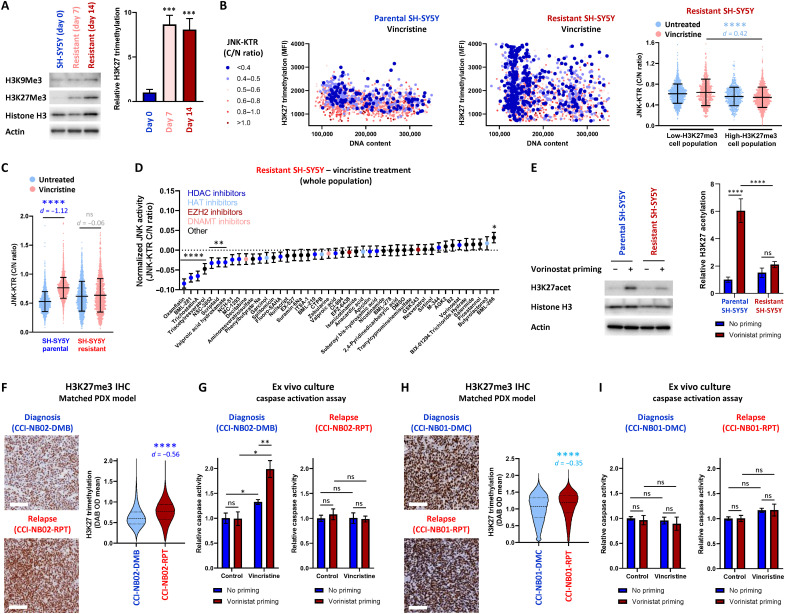
H3K27me3 in resistant populations. (**A**) Western blotting of parental and vincristine-resistant SH-SY5Y cell populations. Quantification was performed on three independent replicates, normalized to total histone H3 (means ± SD. *n* = 3, ***P < 0.01). (**B**) High-content imaging of parental and resistant SH-SY5Y JNK-KTR mRuby2 cells stained with an H3K27me3 antibody and DAPI nuclear marker following vincristine stimulation (300 nM; 2 hours). JNK-KTR cytoplasmic:nuclear ratio values are displayed in the colored bins indicated (*n* = 2000). Quantification within the resistant line was based on a median H3K27me3 cutoff from parental SH-SY5Y cells, as shown in fig. S8C (means ± SD, *n* = 2000, ****P < 0.0001). (**C**) JNK-KTR cytoplasmic:nuclear ratios measured in parental and resistant SH-SY5Y cells following treatment with vincristine (300 nM; 2 hours; means ± SD, *n* = 1897 to 2010, ****P < 0.0001). (**D**) Resistant SH-SY5Y JNK-KTR mRuby2 cells were pretreated with the drugs indicated (100 nM; 24 hours) and then with vincristine (300 nM; 2 hours) before high-content imaging. JNK-KTR cytoplasmic:nuclear ratio values are displayed for the whole cell population (means ± SD, *n* = 1120 to 2254; *P < 0.05, **P < 0.01, and ****P < 0.0001). (**E**) Western blotting performed with lysates from parental and resistant SH-SY5Y cell populations, pretreated with vorinostat (100 nM; 24 hours). Quantification was performed with three independent replicates, and H3K27acet values were normalized to total histone H3 (means ± SD, *n* = 3, ****P < 0.0001). (**F** and **H**) Immunohistochemistry staining of H3K27me3 in the PDX models indicated, along with single-cell quantification (means ± SD, CCI-NB02 *n* = 825,148 and 883,741, respectively; CCI-NB01 *n* = 644,427 and 550,902, respectively; ****P < 0.0001). Scale bars, 100 μm. (**G** and **I**) Caspase activation measured by luminescence assay in ex vivo cultures of PDX models treated with vincristine for 12 hours (means ± SD, *n* = 3, *P < 0.05 and **P < 0.01). Inconsequential effect sizes with *d* < 0.20 are presented in light gray; small effect sizes with *d* = 0.2 to 0.5 are presented in light blue, and large effect sizes with *d* > 0.5 are presented in dark blue.

To further investigate the potential link between elevated H3K27me3, JNK impairment, and sensitivity to vorinostat priming, we evaluated H3K27me3 levels in lysates from parental SH-SY5Y cells (day 0) and resistant cells at both the point of selection with vincristine (day 7) and following regrowth of the cell population (day 14; Fig. 6A). In a similar manner to our stratified cell lines, this analysis revealed that H3K27me3 was significantly higher within the resistant cells at the point of selection and was maintained following the cessation of treatment and expansion of the cell population. This significant increase in H3K27me3 levels within resistant cells was also validated by high-content imaging of H3K27me3 antibody staining in parental and resistant SH-SY5Y JNK-KTR cells (fig. S9B). Correlating this single-cell H3K27me3 staining with JNK activity following vincristine treatment (Fig. 6B) again confirmed that these highly methylated cells within the parental SH-SY5Y line were significantly JNK-impaired (fig. S9C). Furthermore, these highly methylated cells became enriched within the resistant population, where the significant association between high H3K27me3 and JNK impairment at the single-cell level was maintained (Fig. 6B). H3K27me3 chromatin immunoprecipitation sequencing (ChIP-seq) analysis of these cell populations also revealed that the global architecture of H3K27me3 deposition was not altered within these cell populations following drug treatment (fig. S9D). Combined, these observations reinforce the hypothesis that cells with elevated H3K27me3 exist before treatment and are selected on the basis of their impaired ability to activate apoptotic JNK signaling in response to chemotherapy, not due to dynamic remodeling induced by the drug treatment itself.

In line with our previous data (Fig. 3E), directly measuring JNK activity in the parental and resistant SH-SY5Y JNK-KTR mRuby2 cells following vincristine treatment demonstrated that the resistant population of cells is no longer able to significantly activate JNK signaling (Fig. 6C). Therefore, to investigate whether any epigenetic therapies were capable of restoring JNK signaling within this enriched population of JNK-impaired cells, we used our library of epigenetic modifying drugs to prime resistant SH-SY5Y cells before the activation of JNK signaling with vincristine (Fig. 6D). This analysis demonstrated that the ability of all these epigenetic modifiers to restore JNK activity was greatly limited compared to the earlier treatment of treatment-naïve cells (Fig. 4G). In particular, vorinostat elicited a nonsignificant increase in JNK activation. In line with this, priming with vorinostat had a modest sensitizing effect on resistant SH-SY5Y cells treated with vincristine but could not resensitize them to the same level as parental SH-SY5Y cells (fig. S9E). This priming step was also unable to effectively sensitize these resistant cells following treatment with either doxorubicin or topotecan. Furthermore, priming with the EZH2 methyltransferase inhibitor EPZ-6438 was still ineffective at sensitizing the resistant SH-SY5Y cells to all three drugs (fig. S9F), although this resistant population was highly enriched for cells with significantly elevated H3K27me3.

To further investigate this within another model characterized by high-H3K27me3 JNK-impaired cells, we used the SK-N-AS cell line that was originally derived from a relapse tumor (*24*). Following priming of SK-N-AS cells with the library of epigenetic modifiers, there was a significant but greatly reduced ability of vorinostat and other HDAC inhibitors to rescue JNK activity across the whole cell population (fig. S9G). Accordingly, neither vorinostat nor EPZ-6438 priming was capable of increasing caspase activation following vincristine, doxorubicin, or topotecan treatment (fig. S9H). In contrast to the data obtained with SH-SY5Y cells, vorinostat priming was unable to change the distribution of single-cell JNK activity induced by either anisomycin or vincristine, as measured by longitudinal single-cell tracking (fig. S9, I and J). The lack of sensitization achieved by vorinostat priming in these high-H3K27me3 JNK-impaired resistant cell populations is possibly attributed to the inability of this HDAC inhibitor to significantly increase H3K27acet levels in both the resistant SH-SY5Y (Fig. 6E) and impaired SK-N-AS populations (fig. S9K). Collectively, all of these observations suggest that high-H3K27me3 cells can become enriched within resistant cell populations and maintain this methylation within a state that cannot be reversed with an HDAC inhibitor.

Within these cell line models, the long-term maintenance of this JNK-impaired state seems to run counter to the hypothesis that the JNK-impaired cells were initially present simply because of stochastic gene expression noise. However, we also observed this process of stable tumor evolution toward a high-H3K27me3 JNK-impaired phenotype within the two matched PDX models derived from individual patients at both diagnosis and relapse. When comparing the matched CCI-NB02 models, H3K27me3 was significantly higher in the relapsed PDX compared to the original diagnosis PDX (Fig. 6F). Accordingly, vorinostat priming significantly sensitized ex vivo cultures of the low-H3K27me3 diagnosis PDX to vincristine treatment but did not sensitize the high-H3K27me3 relapse PDX (Fig. 6G). In addition, in line with the innate resistance of CCI-NB01 tumor in the clinic and our observation that the CCI-NB01-DMC PDX is JNK impaired (Fig. 3, K and L, and fig. S5D), this diagnosis model displayed elevated H3K27me3, which was only slightly increased in the relapse PDX (Fig. 6H). As expected, in both of these elevated H3K27me3 states, vorinostat priming did not sensitize ex vivo cultures of either the diagnosis or relapse PDX model to vincristine treatment (Fig. 6I).

These combined observations from an in vitro model of drug resistance, an innately resistant cell line, and two clinically derived models of relapse demonstrate that cells with elevated H3K27me3 levels become enriched within drug-resistant populations, potentially because of their inability to activate apoptotic JNK signaling. While these JNK-impaired cells exist initially because of gene expression noise, the selection pressure associated with chemotherapy treatment appears to result in a population of cells that remain within a highly methylated JNK-impaired state. In this state, priming with the HDAC inhibitor vorinostat is no longer able to restore JNK signaling, suggesting that an alternative approach will likely be needed to sensitize relapsed neuroblastomas to standard-of-care chemotherapies.

### Lowering the apoptotic threshold with BH3 mimetics

**Fig. 7.**
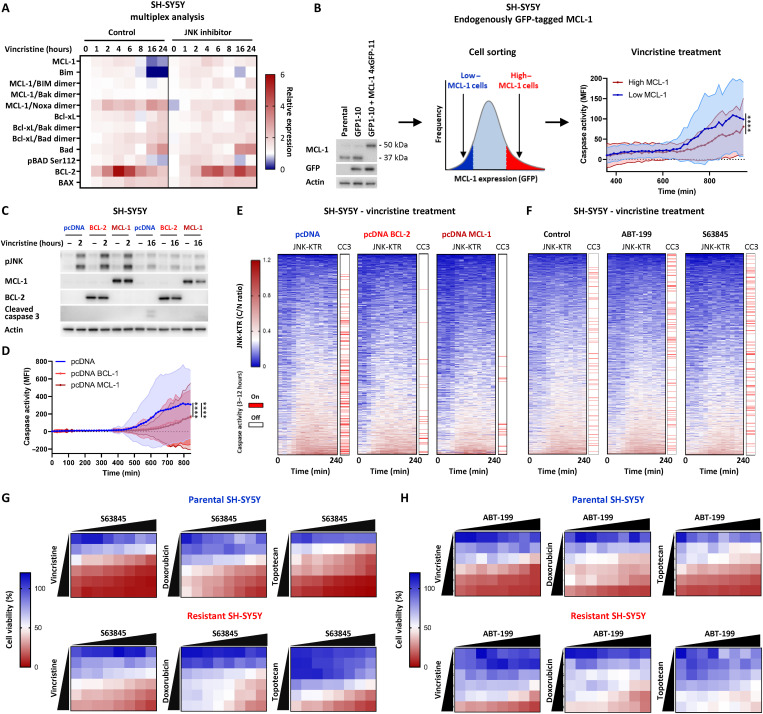
Lowering apoptotic thresholds with BH3 mimetics. (**A**) Multiplexed analysis of apoptotic regulators in SH-SY5Y cells treated with a JNK inhibitor (JNK–IN-8, 10 μM; 30 min) and then vincristine (100 nM) at the time points indicated (mean, *n* = 3). (**B**) Western blotting of an SH-SY5Y line bearing an endogenous tag on MCL-1 (GFP-10 + MCL-1 4× GFP11). The lowest and highest 5% of fluorescent cells were isolated by cell sorting, grown for 24 hours, treated with vincristine (100 nM), and imaged every 15 min for 16 hours in the presence of NucView 530 (means ± SD, *n* = 478 to 902, ****P < 0.0001). (**C**) Western blotting of lysates from SH-SY5Y JNK-KTR mRuby2 cells expressing either an empty vector control (pcDNA), BCL-2, or MCL-1. Cells were treated with vincristine (100 nM) for the time periods indicated. (**D**) Longitudinal high-content imaging of the SH-SY5Y JNK-KTR mRuby2 pcDNA, BCL-2, and MCL-1 cell lines in the presence of NucView 488, following treatment with vincristine (100 nM). Cells were imaged every 15 min for 14 hours (means ± SD, *n* = 1044, 822, and 906; ****P < 0.0001). (**E**) Single-cell tracking of the cells in (D). (**F**) Longitudinal single-cell tracking of SH-SY5Y JNK-KTR mRuby2 cells in the presence of NucView 488, treated with vincristine (100 nM) and either ABT-199, S63845, or a DMSO control. Cells were imaged every 15 min for 12 hours (*n* = 1145, 1128, and 933). (**G**) Cytotoxicity assays performed in parental and resistant SH-SY5Y cell populations, treated with either vincristine (0 to 100 nM), doxorubicin (0 to 500 nM), or topotecan (0 to 500 nM) in the presence of S63845 (0 to 1000 nM) (mean, *n* = 6). (**H**) Cytotoxicity assays performed in parental and resistant SH-SY5Y cell populations, treated with either vincristine (0 to 100 nM), doxorubicin (0 to 500 nM), or topotecan (0 to 500 nM) in the presence of ABT-199 (0 to 1000 nM; mean, *n* = 6).

To further investigate potential treatment strategies aimed at overcoming the JNK-impaired state of relapsed neuroblastoma, we sought to understand the mechanistic basis underlying the conversion of JNK signaling into an apoptotic response. To do this, we performed an analysis of vincristine-induced apoptotic signaling in SH-SY5Y cells, with and without a JNK inhibitor, using a multiplexed bead-based platform (Fig. 7A). This analysis identified significant vincristine-induced, JNK-dependent degradation of the antiapoptotic proteins MCL-1 and BIM after 16 to 24 hours and, to a lesser extent, Bad and BCL-2 after 24 hours (fig. S10A). We also observed JNK-dependent phosphorylation of MCL-1 (Thr^163^) between 2 and 8 hours and of BCL-2 (Thr^56^) between 16 and 24 hours (fig. S10B), both of which are known to promote increased degradation of their cognate protein (*28*, *29*). Confirming the general nature of this apoptotic mechanism, the JNK-dependent degradation of MCL-1 and BCL-2 was also confirmed following the treatment of both SH-SY5Y and NB69 cells with vincristine, doxorubicin, and topotecan (fig. S10C).

As these data were collected using methods that produce an averaged response of a multitude of single-cell responses and we know that JNK activation displays broad single-cell heterogeneity, we sought to gain an understanding of the contribution of gene expression noise to the processing of apoptotic signaling from JNK through MCL-1. To do this, we endogenously tagged MCL-1 in the SH-SY5Y cell line using a split–green fluorescent protein (GFP) system, whereby we stably expressed the nonfluorescent GFP1-10 fragment in the SH-SY5Y cells and then introduced four repeats of the complementary GFP11 fragment (corresponding to the 11th β strand of the superfolder GFP β-barrel structure) at the C terminus of the *MCL-1* gene through CRISPR-mediated tagging (Fig. 7B). By using fluorescence-assisted cell sorting, we isolated the lowest 5% and highest 5% of cells on the basis of MCL-1 expression. There was no significant difference in vincristine-induced JNK phosphorylation between these two cell populations (fig. S10D). However, by tracking the time course of vincristine-induced caspase cleavage in these cells, we observed that the “high” MCL-1 cells were significantly more resistant than the “low” MCL-1 cells. This finding suggests that the single-cell distribution of MCL-1 and, by extension, BCL-2 adds another layer of gene expression noise that could contribute to the stochasticity of the apoptotic response downstream of JNK by altering the intrinsic apoptotic threshold on a single-cell basis.

To further demonstrate this principle, we stably overexpressed MCL-1 and BCL-2 in the SH-SY5Y JNK-KTR line, which again did not alter vincristine-induced JNK activation but did prevent caspase activation (Fig. 7C). Tracking the time course of vincristine-induced caspase activation in these cells confirmed the significant reduction in apoptosis within both the MCL-1– and BCL-2–overexpressing cell lines (Fig. 7D). Furthermore, single-cell tracking also demonstrated that overexpressing either BCL-2 or MCL-1 not only decreased caspase activation but also increased the single-cell threshold of JNK activity required for the downstream activation of caspases (Fig. 7E).

Given the importance of MCL-1 and BCL-2 for integrating the noise of JNK signaling into an apoptotic response, we performed single-cell tracking in the presence of the BH3-mimetic drugs: the MCL-1 targeting S63854 and the BCL-2 targeting ABT-199 (venetoclax; Fig. 7F). Both of these therapeutic agents increased the vincristine-induced activation of caspases, which also occurred within cells that were previously below the identified single-cell apoptotic threshold of JNK activation. Given that all of our models of chemotherapy resistance resulted in impaired JNK activation, this finding prompted us to investigate the potential for synergy between these BH3-mimetic drugs and standard-of-care chemotherapy drugs in both primary and resistant neuroblastoma cells. This analysis demonstrated that both S63845 (Fig. 7G and fig. S11A) and ABT-199 (Fig. 7H and fig. S11B) were highly synergistic with three standard-of-care chemotherapy drugs—vincristine, doxorubicin, and topotecan—in parental SH-SY5Y cells. Within the resistant SH-SY5Y cells, both of these BH3 mimetics displayed some level of synergy with all three chemotherapy drugs (Fig. 7, G and H, and fig. S11, A and B), yet they could not completely restore the initial levels of sensitivity observed in the primary SH-SY5Y cells.

### Targeting single-cell heterogeneity in primary neuroblastoma

**Fig. 8.**
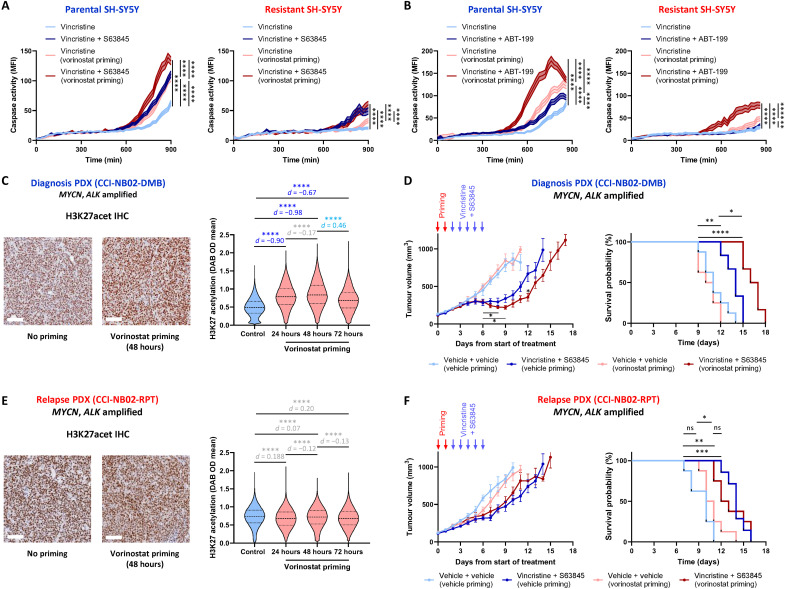
Targeting gene expression noise in primary and relapsed neuroblastoma. (**A**) Longitudinal high-content imaging of parental and resistant SH-SY5Y cells in the presence of NucView 488, pretreated with vorinostat (100 nM) or a DMSO control, and then treated with either vincristine (100 nM) or vincristine and S63845 (100 and 250 nM, respectively; means ± 95% CI, *n* = 361 to 1883, ****P < 0.0001). (**B**) Parental and resistant SH-SY5Y cells treated and imaged as in (A), with the inclusion of ABT-199 instead of S63845 (means ± 95% CI, *n* = 582 to 1551, ****P < 0.0001). (**C**) Immunohistochemistry of H3K27acet in the CCI-NB02-DMB PDX model. NSG (nonobese diabetic–severe combined immunodeficient–gamma) mice were implanted with 1 × 10^6^ tumor cells, and once tumors reached 200 mm^3^, the mice were treated with vorinostat (125 mg/kg, i.p.) each day for up to 3 days (means ± SD, *n* = 476,064 to 621,432, ****P < 0.0001). Scale bars, 100 μm. (**D**) NSG mice were implanted with 1 × 10^6^ CCI-NB02-DMB cells, and once tumors reached 100 mm^3^, the mice were treated with either vorinostat (125 mg/kg) or vehicle once daily for 2 days. This was followed by treatment with either vincristine (0.2 mg/kg, i.v.) plus S63845 (25 mg/kg, i.v.) or the relevant vehicle controls once daily for 5 days. Tumor growth was measured every day until ethical end point (1000 mm^3^; means ± SEM, *n* = 8 for vehicle priming arms and *n* = 6 for vorinostat priming arms; *P < 0.05, **P < 0.01, and ****P < 0.001). (**E**) Immunohistochemistry of H3K27acet in the CCI-NB02-RPT PDX model, treated as in (C) (means ± SD, *n* = 375,615 to 670,344, ****P < 0.0001). Scale bars, 100 μm. (**F**) Tumor growth and survival analysis of the CCI-NB02-RPT PDX model, treated as in (D) (means ± SEM, *n* = 7 vehicle priming and combination therapy arm and *n* = 8 for all other arms; *P < 0.05, **P < 0.01, and ***P < 0.001). Inconsequential effect sizes with *d* < 0.20 are presented in light gray. Small effect sizes with *d* = 0.2 to 0.5 are presented in light blue. Large effect sizes with *d* > 0.5 are presented in dark blue.

By investigating the impact of gene expression noise on the components of the chemotherapy-induced JNK network in neuroblastoma cells, we have now shown that the HDAC inhibitor vorinostat can restore JNK activity to otherwise apoptotic-impaired cells and that BH3 mimetics can increase sensitivity to chemotherapy by lowering the single-cell apoptotic threshold set by the downstream antiapoptotic proteins MCL-1 and BCL-2. To evaluate whether each of these layers of the apoptotic pathway functions as independent regulatory steps, each contributing to the emergent stochasticity of apoptotic signaling, we performed vorinostat priming and combination therapy with the BH3 mimetics in the SH-SY5Y parental and resistant cells (Fig. 8, A and B). By tracking vincristine-induced caspase activation in the SH-SY5Y parental cells, we observed that vorinostat priming followed by combination therapy with vincristine and S63845 significantly increased caspase activation over and above that induced by either vorinostat priming followed by vincristine or a control priming condition followed by vincristine and S63845 (Fig. 8A). This pattern was also recapitulated by performing the same experiment with the BCL-2 inhibitor ABT-199 (Fig. 8B) and also by replacing vincristine with topotecan, mafosfamide (cyclophosphamide analog), or doxorubicin (fig. S11, C to F). The only exception to this pattern was the use of doxorubicin combined with S63845, which was also the least synergistic combination within the cytotoxicity assays (fig. S11A). Nonetheless, the combined benefit of these two approaches demonstrates that targeting multiple layers of heterogeneity in apoptotic signaling concurrently can improve the response to chemotherapy in treatment-naïve neuroblastoma cells. However, none of these conditions were capable of resensitizing the resistant SH-SY5Y cells to the levels of apoptosis induction observed in the primary SH-SY5Y line (Fig. 8, A and B, and fig. S11, C and D), again underscoring the need to focus on improving first-line therapies to prevent the occurrence of highly resistant relapsed tumors.

Having demonstrated this principle within our in vitro model of drug resistance, we performed an in vivo analysis with the *MYCN*-amplified CCI-NB02 diagnosis and relapse PDX models (Fig. 8, C to F). Within both of these PDX models, neither vincristine (0.2 mg/kg, i.v.) nor S63845 [25 mg/kg, intraperitoneally (i.p.)] had any effect on tumor growth or survival as a single-agent therapy administered for five consecutive days (fig. S11, G and H). We next sought to establish the timing of vorinostat priming required in vivo and demonstrated that two daily doses of vorinostat (125 mg/kg, i.p.) produced the peak levels of H3K27acet within the CCI-NB02-DMB diagnosis model (Fig. 8C). By combining 2 days of vorinostat priming with subsequent 5 days of combination therapy with vincristine and S63845, we could demonstrate that priming alone did not affect tumor growth or survival (Fig. 8D). However, vorinostat priming followed by combination therapy resulted in a significant increase in overall survival, along with significant tumor regression in the 3 days following treatment and a significant delay in tumor growth compared to all other conditions, including combination therapy without priming (*P* < 0.05 at day 12; Fig. 8D). These findings demonstrate the ability of vorinostat priming to sensitize chemoresistant cells within the treatment-naïve tumor. However, this HDAC inhibitor did not increase H3K27acet in the CCI-NB02-RPT PDX following vorinostat priming (Fig. 8E) and did not sensitize this relapse model to combination therapy, with no significant difference between these treatment arms for either tumor growth or survival time (Fig. 8F). This result again demonstrates that neither priming with an HDAC inhibitor nor combination therapy with BH3 mimetics is able to overcome the resistant state within a relapse tumor. While additional work will be required to develop these approaches for clinical utilization, our data nonetheless highlight the potential for targeting the noise of apoptotic signaling as a strategy to improve response in the context of frontline standard-of-care chemotherapy.

## DISCUSSION

The clonal selection of resistant cancer cells during the course of chemotherapy or targeted therapy is typically considered to be driven by genetic mechanisms. However, there is an emerging recognition that nongenetic mechanisms may also play a significant role in this process (*7*). Within this field, a number of studies have demonstrated that gene expression noise can contribute to nongenetic drug resistance through the emergence of a population of rare cells with high expression levels of individual genes that promote this resistant phenotype (*14*, *30*). Here, we have demonstrated that chemoresistant cells can arise with higher frequency as an emergent property of a functional network of genes existing within a regulatory structure capable of integrating and amplifying the expression noise associated with each individual component. In contrast to this, a number of regulatory systems have evolved to buffer gene expression noise and thereby increase fidelity within both transcriptional and signaling networks (*31*). A key example of this is the negative feedback from extracellular signal–regulated kinase (ERK) to Raf-1 that confers robustness to cell fate decisions at the population level despite fluctuations in protein levels within the ERK pathway between individual cells (*32*). Conversely, we have now shown that the ultrasensitive response produced by the positive feedback from JNK to MKK7 (*8*) serves to amplify the noise of JNK signaling across a cell population and significantly increases the single-cell heterogeneity of apoptotic signaling.

From an evolutionary viewpoint, this form of single-cell heterogeneity may have emerged as a mechanism to confer a survival advantage during environmental stress, as has been demonstrated in yeast (*33*). Accordingly, genes involved in the stress response are known to be generally noisier when compared to proteasomal (*34*) or protein synthesis genes (*35*). In line with this, stress-induced ultrasensitivity in JNK activation has previously been shown to occur across several model systems (*18*, *36*). Our data now demonstrate that this heterogeneity is also used by cancer cells to ensure the survival of a remnant population following treatment with chemotherapy. The existence and persistence of such a stochastic population of chemoresistant cells within a tumorare not incompatible with either the selection of a clonal population of cells based on pre-existing low-frequency somatic mutations or the acquisition of de novo mutations during treatment. All these mechanisms are likely to occur concurrently and may even display a degree of interdependency, where the initial survival of cells via nongenetic mechanisms may facilitate the accrual of somatic mutations through a form of adaptive mutagenesis (*37*).

Unlike the selection or acquisition of genetic mutations, stochastic gene expression noise is not generally considered a heritable characteristic. As the population of JNK-impaired chemoresistant cells that we have identified existed before treatment because of gene expression noise, it may be unexpected that this phenotype was maintained following chemotherapy. Nonetheless, we observed both enrichment and memory of the JNK-impaired state within each of our in vitro, in vivo, and clinical models of drug resistance. Previous studies of melanoma cells treated with the B-Raf inhibitor vemurafenib also observed that a population of rare cells with transiently high expression of specific resistance-mediating genes survived this drug treatment and then retained a memory of their initially random expression state (*30*). Under homeostatic conditions, fluctuations in single-cell expression levels are thought to vary slowly over the course of multiple cell divisions (*9*, *15*), although it is thought that an epigenetic memory of a cellular perturbation can be maintained over a longer term because of the relatively slow rate of chromatin dynamics (*12*). Changes in histone methylation are known to maintain a memory of environmental stress in *Saccharomyces cerevisiae* and *Caenorhabditis elegans* (*38*), while the epigenetic imprinting of drug resistance originating from nongenetic mechanisms has also been observed in other tumor types (*39*).

While further work is required to demonstrate a direct causal relationship between elevated H3K27me3 and JNK impairment within neuroblastoma cells, the enrichment of this repressive chromatin mark within resistant cells may give further insight into the stability of this cell state. Transient “drug-tolerant persister” and stem cell–like populations have previously been identified within a number of tumor types, although these cells were characterized by low levels of the active chromatin mark H3K4me2/3 (*40*, *41*). In contrast, our identification of a heritable JNK-impaired state in cells with elevated H3K27me3 suggests that maintenance of these states may be dependent on a mechanism involving repressed chromatin. This hypothesis is strengthened by previous studies observing that parental nucleosomes from regions of repressed chromatin are deposited back into the same position following DNA replication and cell division, whereas nucleosomes from regions of active chromatin become dispersed, suggesting that only these repressive chromatin structures can be heritable across multiple cellular generations (*42*).

Studies in yeast have also demonstrated that higher variability in gene expression is positively correlated with nucleosome density around the transcription start site and that inherently noisy genes are therefore likely to be more sensitive to perturbation of chromatin regulators (*12*). This strengthens our rationale for the use of epigenetic modifiers to target the noise of apoptotic signaling, where we expected that the established EZH2 inhibitors, EPZ-6438 and GSK-343, would be effective at reducing H3K27me3, restoring JNK activity, and sensitizing these resistant cells. Our single-cell imaging of SH-SY5Y cells demonstrated that the levels of EZH2 positively correlated with both H3K27me3 and a JNK-impaired state, potentially in line with previous observations showing that EZH2 expression is increased in neuroblastoma (*43*) and associated with a poor patient outcome (*44*). While long-term treatment with higher concentrations of EPZ-6438 was able to significantly reduce H3K27me3 and elevate JNK activity within impaired cells, the emergence of a number of HDAC inhibitors from our JNK activity restoration screen suggested that reducing H3K27me3 alone was not the most efficient method to restore JNK activation potential. While the exact mechanism underlying the ability of HDAC inhibitors to restore JNK activity remains to be elucidated, our data suggest that the ability to alter the equilibrium between both histone acetylation and methylation may make this class of drugs more effective in this context.

A number of HDAC inhibitors have already been used within both preclinical studies and clinical trials for neuroblastoma. However, of these, only vorinostat and panobinostat have progressed through to phase 2 clinical studies. Vorinostat is a class I/II HDAC inhibitor, which has activity against neuroblastoma cells as a single agent, as well as in combination with the standard-of-care chemotherapy drug doxorubicin and with radiotherapy (*26*). However, none of these studies have investigated the potential use of vorinostat as a priming agent capable of restoring apoptotic capability to cells before treatment with standard-of-care chemotherapy. The use of a priming or sequential treatment regimen has previously been suggested as a method to rewire apoptotic signaling networks (*45*) and is an emerging approach in other tumor types (*46*). This rationalized sequential delivery has several advantages over typical combination therapies, foremost though is the potential to maximize the response to chemotherapy while minimizing the toxicity that is often compounded by combination therapies. This priming approach was highly successful within both our in vitro and in vivo models of primary treatment-naïve neuroblastoma, although the mechanism underlying resistance to vorinostat in models of resistant and relapsed neuroblastoma remains to be fully elucidated. Resistance to HDAC inhibitors in general is an emerging and active field of research for a number of tumor types (*26*), although in the context of this study, the potential for the maintenance of an irreversible, highly methylated state is one possibility, as is the loss of specific histone acetyl transferases within these resistant cells. Furthermore, while the basal levels of H3K27ac did not influence single-cell heterogeneity in JNK activation within our study, they have been shown to define four separate super-enhancer–driven epigenetic subtypes of neuroblastoma (*47*), with the potential to influence HDAC inhibitor–induced changes in this chromatin mark.

In an effort to overcome this insensitivity to HDAC inhibitors, we investigated the potential for targeting the apoptotic machinery downstream of chemotherapy-induced JNK activity. In this context, the combination of HDAC inhibitor priming and BH3-mimetic combination therapy is a mechanistically coherent approach involving the “upstream” restoration of JNK activity within resistant cells and the “downstream” lowering of apoptotic thresholds. In support of this logical approach, our data demonstrated that HDAC inhibitor priming and combination therapy with BH3 mimetics in vitro resulted in an additive effect to chemotherapy-induced caspase activation, supporting the hypothesis that these therapeutic interventions act at different points within the apoptotic cascade. Within this framework, the inability of the BH3 mimetics to fully resensitize JNK-impaired models of resistance is in line with the upstream requirement of JNK activity to promote the downstream degradation of MCL-1/BCL-2 and induce apoptosis (Fig. 7A). This targeted inhibition of BCL-2 family members has previously been shown to inhibit neuroblastoma tumor growth as both single agents and in combination with standard-of-care therapy (*48*), although, in the setting of relapsed neuroblastoma, it appears that the ability of these drugs to lower the apoptotic threshold is not sufficient to overcome the JNK impairment of these resistant tumors, which should be noted for the further clinical development of this class of drugs.

The highly resistant nature of relapsed neuroblastoma, which was observed across all our in vitro and in vivo models, is a salient lesson in the need to develop more effective first-line therapies and reduce the occurrence of this relapsed state. Furthermore, our data also highlight the need to specifically trial these potential frontline therapies within this clinical context. It is common for many emerging drugs to be trialed in late-stage disease where their efficacy may be predictably limited. However, for HDAC inhibitor priming to have efficacy, it would need to be trialed as a first-line therapy, likely before receiving induction therapy such as Rapid COJEC. Combination therapy with BH3 mimetics would also be most beneficial within an induction therapy regimen, before the acquisition of a resistant JNK-impaired phenotype.

While further work will be required to develop this approach into a clinically relevant therapeutic strategy, from a mechanistic viewpoint, the combined effectiveness of HDAC inhibitor priming and combination therapy with BH3 mimetics is a clear demonstration of the multiple layers of gene expression noise that ultimately manifest the stochasticity of apoptosis. A wealth of recent research has focused on the development and personalization of targeted therapies to treat neuroblastoma and many other tumors. However, our work now unveils the opportunities that exist to improve therapeutic response with both existing and emerging treatments, through the application of a detailed understanding of the fundamental processes that promote heterogeneity in drug-induced apoptotic signaling at the single-cell level.

## MATERIALS AND METHODS

### Study design

All in vitro experiments were performed and quantified in at least biological triplicates, with relevant sample sizes reported for each experiment. The sample size for all single-cell high-content imaging experiments is also reported for each individual experiment. The criteria for the analysis of statistical significance and noise within these datasets are described below. In vivo xenograft studies were randomized, with relevant group sizes reported for each individual experiment.

### Reagents

The source and details for all reagents are listed in table S2.

### Experimental model and subject details

The SH-SY5Y, NB69, SK-N-AS, SK-N-FI, and BE(2)-C neuroblastoma cell lines were all maintained in phenol-red RPMI 1640 with 10% fetal bovine serum (FBS) and penicillin-streptomycin under standard tissue culture conditions (5% CO_2_ and 20% O_2_). The human embryonic kidney (HEK) 293T cell line was cultured in Dulbecco’s modified Eagle’s medium containing 10% FBS under standard tissue culture conditions (5% CO_2_ and 20% O_2_). All cell lines were authenticated by short tandem repeat polymorphism, single-nucleotide polymorphism, and fingerprint analyses; passaged for less than 6 months; and confirmed as negative for mycoplasma contamination using the MycoAlert luminescence detection kit (Lonza, Switzerland). Stable cell lines expressing the KTR biosensors were generated by lentiviral transduction as previously described (*49*). Confocal fluorescence imaging of these biosensor-expressing lines was performed as previously described (*50*).

The neuroblastoma tumor microarray used for immunohistochemistry analysis was provided by F.S. (Ghent University). This cohort consisted of 83 primary neuroblastoma tumor samples from untreated patients, as previously described (*51*).

### Cell-based assays

Cytotoxicity assays were performed using the CellTiter 96 aqueous nonradioactive cell proliferation assay or the Caspase-Glo 3/7 Assay System from Promega (USA), according to the manufacturer’s instructions. Quantification of synergy within cytotoxicity assays was performed with SynergyFinder 2.0 (*52*). Plasmid transfection of the neuroblastoma cell lines was performed using JetPRIME (Polyplus Transfection) according to the manufacturer’s instructions.

### Flow cytometry

To derive single-cell distributions for the JNK network model components, multicolor flow cytometry was performed with fluorescently conjugated primary antibodies. Briefly, 3 × 10^6^ SH-SY5Y cells were seeded into 15-cm plates and incubated under standard tissue culture conditions for 24 hours. Cells were then harvested with phosphate-buffered saline (PBS)/EDTA before fixation with 4% paraformaldehyde (PFA) for 15 min at room temperature. Following centrifugation, the cell pellet was washed with PBS and then permeabilized with PBS/1% bovine serum albumin (BSA)/0.1% Triton X-100 for 5 min at room temperature. After two washes with PBS/1% BSA, cells were resuspended at 2.5 × 10^6^ cells/ml, and 200-μl aliquots were used for each antibody incubation (1:100). Following two washes with PBS/1% BSA, flow cytometry analysis was performed on the FACS Symphony A5 (Becton Dickinson). Individually labeled cells were used to perform compensation between each fluorophore, before the five-color staining analysis.

### High-content imaging

All fixed and live-cell high-content imaging was performed with a Cellomics ArrayScan VTI HCS Reader (Thermo Fisher Scientific). Analysis of the generated images was performed using the nuclear translocation settings within the ArrayScan analysis software (version 6.1.2.6060). Briefly, a nuclear mask was generated using images obtained from a nuclear counterstain, and a 4-pixel cytoplasmic ring was generated in all directions except into the nuclei of neighboring cells. This nuclear/cytoplasmic mask was overlaid onto images of the KTR biosensor, allowing quantification of the cytoplasmic-to-nuclear ratio using the average intensity values of the biosensor, which is directly proportional to kinase activity (*17*). For antibody staining, EdU incorporation, or caspase activation, the average intensity values were used from either the nuclear or cytoplasmic mask, as appropriate for the subcellular localization of the staining. For nuclear counterstains, the total nuclear intensity values were used.

For single time-point imaging of the SH-SY5Y JNK-KTR mRuby cells, 5 × 10^5^ cells were seeded into Cellstar μclear cell culture 96-well F-bottom microplates (Greiner Bio-One) in 100 μl of growth media and cultured for 24 hours. Nuclear counterstaining was performed through the addition of 50 μl of growth media containing Hoechst 33342 (final dilution, 1:1000) for 30 min, before the addition of a further 50 μl of growth media containing anisomycin (final concentration, 300 nM). After 30 min, cells were imaged and analyzed as above. Hoechst 33342 was imaged with the BGRFR_386_23 channel, and the JNK-KTR mRuby2 biosensor was imaged with the BGRFR_549_15 channel.

For longitudinal single-cell imaging and tracking of the JNK-KTR biosensor in NB69, SH-SY5Y, SK-N-AS, and HEK-293T lines, cells were seeded as above, although the far-red nuclear marker SiR-DNA was used for 2 hours (final dilution, 1:2000) before stimulation with either anisomycin or vincristine (final concentration, 300 nM). Kinetic tracking was enabled within the ArrayScan analysis software using standard parameters, and individual cells that were tracked continuously throughout the entire imaging period were filtered and analyzed. SiR-DNA was imaged with the BGRFR_650_13 channel, and the JNK-KTR mRuby2 biosensor was imaged with the BGRFR_549_15 channel.

For longitudinal simultaneous single-cell tracking of the JNK-KTR mRuby2 biosensor and caspase activation with NucView 488 in the SH-SY5Y (parental, pcDNA/BCL-2/MCL-1), NB69 and SK-N-AS cells were seeded as above, and nuclear counterstaining was performed with SiR-DNA. Vincristine (100 nM) and NucView 488 Caspase-3 enzyme substrate (1 μM; Biotium) were added, and images were taken at 15-min intervals over a 12- to 16-hour time period with kinetic tracking enabled. For the combined analysis of the JNK-KTR biosensor and caspase activation in the NB69 and SK-N-AS lines, only cells that were tracked throughout the entire imaging period were filtered and analyzed. For the more motile SH-SY5Y line, all cells that were continuously imaged for at least the first 8 hours were filtered and analyzed. An average fluorescence intensity of >50 within an individual cell was considered a positive caspase activation event. Individual cells expressing very low (<10 mean fluorescence intensity) or very high (>200 mean fluorescence intensity) levels of the biosensor were excluded from this analysis. The NucView 488 Caspase-3 enzyme substrate was imaged with the BGRFR_485_20 channel; the JNK-KTR mRuby2 was imaged with the BGRFR_549_15 channel, and the SiR-DNA nuclear marker was imaged with the BGRFR_650_13 channel. For the analysis of caspase activation only in the SH-SY5Y (parental, resistant, and endogenously tagged MCL-1 lines), no filtering was performed, and all cells were analyzed regardless of the length of tracking. The NucView 488 Caspase-3 enzyme substrate was imaged with the BGRFR_485_20 channel; the NucView 530 Caspase-3 enzyme substrate was imaged with the BGRFR_549_15 channel, and the SiR-DNA nuclear marker was imaged with the BGRFR_650_13 channel.

To perform costaining along with the JNK-KTR, cells were seeded as above and stimulated as required with the absence of a DNA counterstain. Cells were then fixed rapidly with prewarmed 4% PFA for 30 min at room temperature. Samples were then washed twice with PBS, permeabilized with 0.1% Triton X/PBS for 5 min, and blocked for 30 min in a 2% BSA/PBS blocking solution. For unconjugated antibodies, samples were incubated in primary antibody solutions diluted in blocking buffer for 1 hour at room temperature, washed twice with PBS, and subsequently labeled with secondary antibodies diluted in blocking buffer for 30 min at room temperature. For preconjugated antibodies, samples were incubated in antibody solutions in blocking buffer for 1 hour at room temperature. When both unconjugated and preconjugated antibodies were combined, protocols were performed sequentially with the addition of an intermediate 2% normal goat serum/PBS blocking step. For cell cycle analysis, the Click-iT EdU Flow Cytometry Assay Kit (C10632; Thermo Fisher Scientific) was used according to the manufacturer’s instructions. Briefly, cells fixed with 4% PFA for 30 min were washed twice with PBS, incubated in saponin-based permeabilization and wash solution for 15 min, blocked in 2% BSA/PBS blocking buffer for 15 min, and incubated with 1× Click-iT reaction cocktail for 30 min at room temperature. In all cases, samples were washed twice with PBS, counterstained with 4′,6-diamidino-2-phenylindole (DAPI; 1:1000) for 5 min, washed twice with PBS, and imaged as described above. DAPI was imaged with the BGRFR_386_23 channel, the JNK-KTR mRuby2 biosensor was imaged with the XF53_572_15 biosensor, antibody staining was imaged with either the BGRFR_485_20 or BGRFR_650_13 channel depending on the relevant fluorophore, and EdU staining was imaged with the BGRFR_485_20 channel. Note that the protocol for EdU detection resulted in an upward shift in all JNK-KTR ratio values.

For screening of epigenetic modifying compounds, the SCREEN-WELL Epigenetics library (BML-2836-0100) from Enzo Life Sciences (USA) was used, supplemented with EPZ-6438 and GSK-343. For this analysis, SH-SY5Y (parental and resistant) and SK-N-AS cells were seeded as above and then incubated with 100 nM each compound for a further 24 hours, before stimulation with anisomycin or vincristine (300 nM) and staining with Hoechst 33342 for 30 min. Cells were then fixed rapidly with prewarmed 4% PFA for 30 min at room temperature and imaged as described above.

### Mathematical modeling

All simulations of JNK activity were performed using a dynamic ODE model constructed through rule-based modeling that we have fully described previously (*8*). Briefly, this model consists mainly of the three-tiered JNK kinase cascade: a MAP3K (ZAK), two MAP2K kinases (MKK4 and MKK7), and MAPKs (JNK1/2). In addition to this core structure, the model also contains a positive feedback loop mediated via JNK phosphorylation of Thr^66^ and Thr^83^ in the N-terminal region of MKK7 and inhibitory cross-talk through Akt-mediated phosphorylation of MKK4 (Ser^80^) and MKK7 (Thr^385^). All simulations and predictions were performed following the calibration and validation steps outlined in our previous work and executed using MATLAB R2020a.

To simulate JNK activity in different cell lines, input parameters were generated by measuring the relative abundance of each component of the model (i.e., ZAK, MKK7, MKK4, JNK, and phosphorylated Akt Ser^473^) through quantitative Western blotting and densitometry. As this model was initially constructed using the SH-SY5Y cell line, all values were normalized to those obtained for the parental SH-SY5Y cell line.

For single-cell simulations of JNK activity, the relative distribution of each model component was measured using the five-color flow methodology described above. The single-cell fluorescence intensity values were exported for each component and normalized to the mean value obtained using the parental SH-SY5Y cell line. These relative single-cell expression values were imported into the model as input parameters, allowing the prediction of JNK activity in each individual cell. A saturated response was generated by simulating an input of 1000 arbitrary stress units. Basal levels of JNK activity were simulated using an input strength set to 1/20th of the saturated response rather than a nonphysiological input of zero. To perform these single-cell simulations without positive feedback, the model parameters describing the interaction between JNK and MKK7 were set to 0 using commands in the MATLAB console.

For the conversion of cytoplasmic:nuclear ratios obtained with the JNK-KTR biosensor to concentration values of active JNK, we used the ODE model and model parameters previously developed for these biosensors by the original authors (*17*). A saturation point for active kinase concentration was set at a value of 200 to prevent imprecisions in the measurements from producing large variations in the output values. For the purpose of comparison, simulated data were normalized to the highest simulated value of JNK activation, and similarly, measurements of active JNK concentration were normalized to the highest value recorded. This method provided a straightforward nonbiased means to compare these distributions despite the difference in scale.

### Western blotting and immunoprecipitation

Lysates for Western blotting were prepared using normal lysis buffer [50 mM tris-HCl (pH 7.4), 150 mM NaCl, 1 mM EDTA, and 1% (v/v) Triton X-100] containing protease inhibitor cocktail (p8340, Sigma-Aldrich) and 0.2 mM sodium orthovanadate. SDS–polyacrylamide gel electrophoresis (SDS-PAGE) and Western blotting were performed using the NuPAGE SDS PAGE Gel System and NuPAGE Bis Tris Precast Gels (4 to 12%) (Life Technologies). Western Lightning PLUS Enhanced Chemiluminescent Substrate (PerkinElmer) was used for imaging Western blots on the Vilber Lourmat Fusion chemiluminescent imaging system. Quantitative Western blotting was performed using multistrip Western blotting.

### Fluorescence lifetime imaging microscopy/FRET imaging

SH-SY5Y cells were plated in a 35-mm glass-bottom dish at a density of 25,0000 cells/ml. Cells were transiently transfected with 1.5 μg of pcDNA JNKAR1 using jetPRIME transfection reagent. Following transfection, cells were incubated for 16 hours under standard tissue culture conditions and then stimulated with 300 nM anisomycin. Images were taken at 5-min intervals over 1 hour with a Leica TCS SP8 MP using the 40× 1.20 numerical aperture (NA) water immersion lens with a 481/32 filter cube. The donor fluorophore, cerulean, was excited with a wavelength of 840 nm, and the fluorescence lifetime was quantified with FLIMfit 5.1.1 software (*53*) rendered using ImageJ [National Institutes of Health (NIH)] and plotted with GraphPad Prism (version 9).

### Endogenous GFP tagging

#### Generation of stable GFP1-10 cell lines

HEK-293T cells were seeded at 2 × 10^6^ cells per 10-cm dish in 10 ml of media and incubated overnight. Cells were transfected with 4.5 μg of pMDL/pRRE (Addgene, #12251) and 6.4 μg of pRSV-Rev (Addgene, #12253) packaging plasmids, 2.7 μg of envelope plasmid pMD2.G (Addgene, #12259), and 15 μg of pHR-SFFV-GFP1-10 (Addgene, #80409), using Lipofectamine 2000. After 24 hours of incubation, the transfection medium was replaced with fresh culture medium. Lentiviral supernatant was harvested 48 hours later and concentrated 40 times using Amicon Ultra-15 centrifugal filter tubes (Merck, Darmstadt, Germany). To generate stable cell lines expressing GFP1-10, HEK-293T and SH-SY5Y cells were plated at 200,000 per well in 2 ml of media in a six-well plate. Cells were then transduced with concentrated GFP1-10 lentivirus diluted 1:8 in culture medium with polybrene (8 μg/ml). Culture medium was replaced 24 hours later. After a minimum of 72 hours of posttransduction, cells were harvested for lysates, and GFP1-10 expression was confirmed by Western blot analysis.

#### Design of double-stranded DNA Homology-directed repair template primers

The 4× GFP11 double-stranded DNA (dsDNA) donor templates [258 base pairs (bp)] for both N- and C-terminal insertion were synthesized in the pUC57 vector by GenScript (New Jersey, USA), containing four repeats of GFP11 separated by five amino acid linkers, as shown by Leonetti *et al.* (*54*). Homology-directed repair (HDR) templates were generated by polymerase chain reaction (PCR) amplification of the 4× GFP11 donor template with gene-specific primers (IDT, Iowa, USA) using Phusion DNA polymerase (NEB, Massachusetts, USA), resulting in HDR templates with 35- to 45-nucleotide (nt) homology arms. The final PCR product was confirmed on a 1 to 2% agarose gel and purified using the GeneJET PCR Purification Kit (Thermo Fisher Scientific, Massachusetts, USA). The concentration of the HDR template was measured on the NanoDrop before cell transfection.

#### Design of sgRNAs

For each gene, optimal protospacer adjacent motif sequences were identified within 35 nt on either side of the start, for N-terminal tagging, or stop codon, for C-terminal tagging, using http://crispor.tefor.net/ and https://crispr.cos.uni-heidelberg.de/. Alt-R CRISPR-Cas9 single guide RNAs (sgRNAs) were synthesized by IDT (Iowa, USA).

#### Generation of 4× GFP11 endogenously tagged cell lines using CRISPR-Cas9

SH-SY5Y cells were seeded at 100,000 cells per well in 0.5 ml of media in a 24-well plate. Cells were allowed to plate down overnight and then incubated with nocodazole (200 ng/ml) for 16 hours to arrest cells in G_2_-S for more efficient HDR. Culture medium was replaced before transfection using Lipofectamine CRISPRMAX Cas9 transfection reagent (Thermo Fisher Scientific, Massachusetts, USA). Cas9 ribonucleoproteins were prepared by mixing 25 μl of Opti-MEM, 500 ng of HDR donor dsDNA, 1250 ng of TrueCut Cas9 v2 (Thermo Fisher Scientific, Massachusetts, USA), 240 ng of sgRNA, and 2.5 μl of Lipofectamine Cas9 Plus. A second mixture containing 25 μl of Opti-MEM and 1.5 μl of Lipofectamine CRISPRMAX was incubated at room temperature for 1 min. Both mixtures were combined, and the resulting transfection mix was incubated for 10 to 15 min at 37°C. The transfection mixture was added to adherent cells and incubated for 72 hours. Successful knock-in of the 4× GFP11 insert was confirmed via Western blot analysis and confocal fluorescence microscopy. Monoclonal cell lines were established and used for subsequent experiments.

### Intravital imaging with optical windows

For the establishment of primary tumors, BALB/c-Fox1nuAusbJ mice were injected subcutaneously with 4 × 10^6^ SH-SY5Y cells expressing JNK-KTR mClover and H2B-mCherry and engrafted with optical imaging windows once tumors reached an average size of 195 ± 79 mm^3^, as described previously (*55*). Briefly, the surgery was performed following the standard operation procedure “Mammary Window Surgery” approved by the Garvan/St. Vincent’s Animal Ethics Committee (ARA 19/13). All surgical instruments were sterilized by autoclaving or by using a bead sterilizer (Germinator) and equipment, and surfaces were sterilized by wiping down with 70% ethanol and Clidox-S. Cyanoacrylate was applied to the edges of a titanium ring (Russel Symes & Company), and a coverslip with a diameter of 12 mm was placed into the inset of that ring 24 hours before surgery. A minimum of 24 hours before and at least 72 hours after surgery, carprofen (5 mg/kg; Rimadyl) was administered to the mice in the drinking water. During surgery, anesthesia was induced and maintained by inhalation of isoflurane using a calibrated vaporizer, and a subcutaneous injection of buprenorphine (0.075 mg/kg) was used for further pain management before and 6 hours after the surgery. Surgery site was disinfected using 0.5% chlorhexidine/70% ethanol, and the optical windows were inserted and held in place in the skin via a purse string suture (Mersilk, 5-0). The mice were allowed to recover for 72 hours after surgery and weaned off carprofen for a minimum of 24 hours before any treatments and in vivo imaging were performed. Recovery gel and/or sunflower seeds were provided to aid in recovery after surgery. To minimize damage to the window by the mice and cage surroundings, metal food hoppers and plastic domes are removed from the cages, and feed was supplied in food trays on the floor of the cage. Paper-mache domes were further supplied as cage enrichment along with tissues as nesting material.

Before in vivo imaging, mice bearing an optical window were treated with one to five consecutive intravenous injections of saline vehicle or vincristine (1 mg/kg) and imaged under 1 to 2% isoflurane on a heated stage (Digital Pixel, UK). In vivo imaging was performed as described previously using a Leica DMI 6000 SP8 confocal microscope using a 25× 0.95 NA water immersion objective on an inverted stage. The Ti:Sapphire femtosecond laser (Coherent Chameleon Ultra II, Coherent) excitation source operating at 80 MHz was tuned to a pumping wavelength of 920 nm for mClover and second harmonic generation (SHG) and 1040 nm for mCherry, respectively. RLD-HyD detectors were used with 435/40-, 525/50-nm, and 617/60-nm band-pass emission filters to detect the SHG of the collagen I, mClover, and mCherry signals, respectively. Images were acquired at a line rate of 1000 Hz, 512 × 512 pixel, and at a total of 175 frames per image. Realignment of the data was performed using Galene (v2.0.2) (*56*) using the warp realignment mode, 10 realignment points, and a smoothing radius of 2 pixels, and a realignment threshold of 0.6 was applied for all channels. Image stacks were processed in ImageJ (NIH) by performing average intensity Z projections. As defining a cytoplasmic region proved difficult within a three-dimensional tumor, single-cell activity of the JNK-KTR biosensor was calculated as a ratio of the mean fluorescence intensity of the biosensor across each entire image over the mean fluorescence intensity of each individual nuclear region within that image. This analysis was performed on three to four regions per mouse and per condition. The values obtained for four individual mice were plotted and analyzed using Prism 9 (GraphPad).

### Whole-genome and whole-genome bisulfite sequencing and data analysis

Genomic DNA was extracted from SH-SY5Y cells and PDX models using the QIAGEN DNeasy Blood & Tissue Kit according to the manufacturer’s instructions. Whole-genome sequencing was performed by the Kinghorn Centre for Clinical Genomics, Garvan Institute of Medical Research (Australia), on the Illumina HiSeq X Ten platform (SH-SY5Y cell lines) and NovaSeq 6000 (PDX models). Sequencing libraries were prepared using 1 μg of DNA using the TruSeq Nano DNA HT Sample Prep Kit (Illumina). Cell line sequencing was performed with a minimum mean coverage of 30×, whereas early-passage (passage 3) PDX tumors were sequenced to a minimum mean coverage of 60×. Full details of the data analysis and driver variant curation have been previously described (*22*). Copy number analysis was performed on bam index files using Indexcov version 0.1.16, using default settings.

Whole-genome bisulfite sequencing was performed with the MethylC-seq assay. Genomic DNA (1 μg) was fragmented to an average size of 300 bp using a Covaris sonicator, and sonicated DNA was used for MethylC-seq library preparation with the NEXTFLEX Bisulfite Library Prep Kit (PerkinElmer, Waltham, MA, USA) and bisulfite treatment with the EZ DNA-methylation Gold Kit (Zymo Research, Irvine, CA, USA) according to the manufacturer’s instructions. Resultant MethylC-seq libraries were sequenced on the Illumina HiSeq X platform (150-bp paired-end sequencing). The processing of sequenced reads and DNA methylation analysis was performed as previously described (*57*).

### ChIP-seq library preparation, sequencing, and data analysis

H3K27me3 (Cell Signaling Technology, catalog no. 9733) modified histone ChIP was performed with 4 million SH-SY5Y cells for each sample. Three conditions were used: untreated cells, 7 days of vincristine treatment at 150 nM, and 7 days of vincristine treatment at 150 nM treatment + recovery for a further 7 days after the drug removal. The ChIP-seq assay was performed in two biological replicates. Formaldehyde–cross-linked chromatin was fragmented into an average size of 300 bp by sonication with a Bioruptor sonicator (Diagenode). Approximately 5 ng of ChIP-enriched DNA was used for library preparation with the TruSeq ChIP Library Preparation Kit (Illumina) according to the manufacturer’s instructions. Resultant ChIP-seq libraries were sequenced on the Illumina HiSeq X platform (150-bp paired-end sequencing). ChIP-seq data were mapped and analyzed as previously described (*57*). Only H3K27me3 peaks longer or equal to 2 kb were included in the analysis.

### Multiplex analysis

Multiplex analysis was performed using a Bio-Plex MAGPIX system (#171015044) and Bio-Plex Pro-Wash Station (Bio-Rad) as previously described (*58*). Lysates were analyzed using the Bio-Plex Pro RBM Apoptosis Panel 3 (Bio-Rad), the MILLIPLEX MAP Bcl-2 Family Apoptosis Panel 1, and the MILLIPLEX MAP Bcl-2 Family Apoptosis Panel 2 (Merck).

### Immunohistochemistry

Immunohistochemistry was performed on formalin-fixed paraffin-embedded sections using the Leica BOND RX (Leica, Wetzlar, Germany). Slides were first dewaxed and rehydrated. Heat-induced antigen retrieval was performed with citrate (pH 6) retrieval buffer for 20 min at 100°C. Primary antibodies were diluted 1:200 (H3K27me3) and 1:300 (H3K27acet, pJNK^T183/Y185^) in Leica antibody diluent and incubated for 60 min on slides. Antibody staining was completed using the Bond Polymer Refine immunohistochemistry protocol and reagents (Leica, Wetzlar, Germany). Slides were counterstained on the Leica Autostainer XL (Leica, Wetzlar, Germany). Leica CV5030 glass coverslipper (Leica, Wetzlar, Germany) was used, and bright-field images were taken on the Aperio CS2 Slide Scanner (Leica, Wetzlar, Germany). Quantification of single-cell staining intensity was performed using the cell detection function of QuPath (v0.2.3).

### PDX models

All tumor specimens were obtained from patients at the Sydney Children’s Hospital Network (SCHN) under approval by the SCHN Human Research Ethics Committee (LNR/14/SCHN/392, LNR/14/SCHN/497). Informed parental or guardian consent was obtained for each patient. The generation, maintenance, and propagation of the two sets of matched diagnosis and relapse models (CCI-NBO1 and CCI-NB02) have been previously described (*21*). Following expansion of each PDX in nonobese diabetic–severe combined immunodeficient–gamma (NSG) mice, dissociated PDX tumor cells were subcutaneously engrafted into 6-week-old secondary NSG mice (1 × 10^6^ tumor cells per graft in Matrigel) and randomized into treatment groups (eight mice per group). Relevant treatment schedules for each drug or priming combination commenced once tumors reached 100 mm^3^. Tumors were only used for analysis if they reached 100 mm^3^ within a 7-day period following the initiation of treatment. Vincristine was delivered in a PBS vehicle (0.2 mg/kg, i.v.), S63845 was delivered in 20% (2-hydroxypropyl)-β-cyclodextrin made up in 25 mM hydrochloric acid (25 mg/kg i.v.), and vorinostat was delivered in PBS/2% dimethyl sulfoxide (DMSO)/30% polyethylene glycol, molecular weight 300 (125 mg/kg, i.p.). Tumor growth was measured every day, and growth curves were generated. Ethical end point was determined by ulceration, tumor size, or maximum weight loss. Time to end point was assessed using Kaplan-Meier survival curves, which were compared using the log-rank test. All statistical analyses were performed using built-in functions in GraphPad Prism (version 9, GraphPad Software).

To generate ex vivo patient-derived cell cultures from these PDX models, ~20 × 10^6^ to 30 × 10^6^ dissociated cells from each model were plated onto laminin-coated 15-cm tissue culture dishes in Iscove's Modified Dulbecco's Media containing 20% FBS and insulin/transferrin/selenium supplement (1:500). Following a 24-hour incubation under standard tissue culture conditions (5% CO_2_ and 20% O_2_), the tumor cells were harvested with Puck’s EDTA (140 mM NaCl, 5 mM KCl, 5.5 mM glucose, 4 mM NaHCO_3_, 13 μM phenol red, 0.8 mM EDTA, and 9 mM Hepes), which facilitated the selective detachment of tumor cells, while stromal fibroblasts remained attached. Tumor cells were then processed for each functional assay as indicated.

### Statistical tests

All datasets with *n* < 1000 were analyzed using the appropriate *t* test or analysis of variance (ANOVA), depending on the experimental design, and *P* values are reported to support any claim of significance. However, because of the high sample numbers associated with single-cell analysis, all datasets with *n* > 1000 were analyzed with a combined approach that included an effect size analysis along with the appropriate statistical test. For this purpose, we used Cohen’s test, which reports a value (*d*) that reflects an effect size calculated by dividing the difference between two means by the pooled SD of both samples. For this analysis, *d* = 0.2 is considered as a small effect, *d* = 0.5 is considered as a medium effect, and *d* = 0.8 is considered as a large effect size. Therefore, for the purpose of interpreting our high-content imaging and single-cell immunohistochemistry data, we have considered any result with *d* < 0.2 to be inconsequential, even if statistically significant, and presented these values as light gray in the figures. Statistically significant results with *d* between 0.2 and 0.5 are considered to be consequential but with a modest overall effect, and these values are presented as light blue in the figures. Statistically significant results with *d* > 0.5 are considered to be a strong effect and presented as dark blue in the figures.

For longitudinal measurements of single-cell caspase activation, statistical significance between treatment groups was determined by performing pairwise linear regressions within the linear portion of each time course immediately following the initiation of caspase activity. This analysis was performed using the “simple linear regression” function within GraphPad Prism v9.2.

## Notes

View/request a protocol for this paper from *Bio-protocol*.
