# Supplementary material for: Memory of stochastic single-cell apoptotic signaling promotes chemoresistance in neuroblastoma
Source: Sci Adv. 2023 Mar 3;9(9):eabp8314. doi: 10.1126/sciadv.abp8314 (PMC9984174; doi:10.1126/sciadv.abp8314)
Supplement: Supplementary file 1 — Figs. S1 to S11 Legend for data file S1 Tables S1 and S2 [file sciadv.abp8314_sm.pdf]

Science Advances
AAAS

Supplementary Materials for

Memory of stochastic single-cell apoptotic signaling promotes chemoresistance in neuroblastoma

Jordan F. Hastings *et al.*

Corresponding author: David R. Croucher, d.croucher@garvan.org.au

Sci. Adv. **9**, eabp8314 (2023)
DOI: 10.1126/sciadv.abp8314

The PDF file includes:

Figs. S1 to S11
Legend for data file S1
Tables S1 and S2

Other Supplementary Material for this manuscript includes the following:

Data file S1

Figure S1

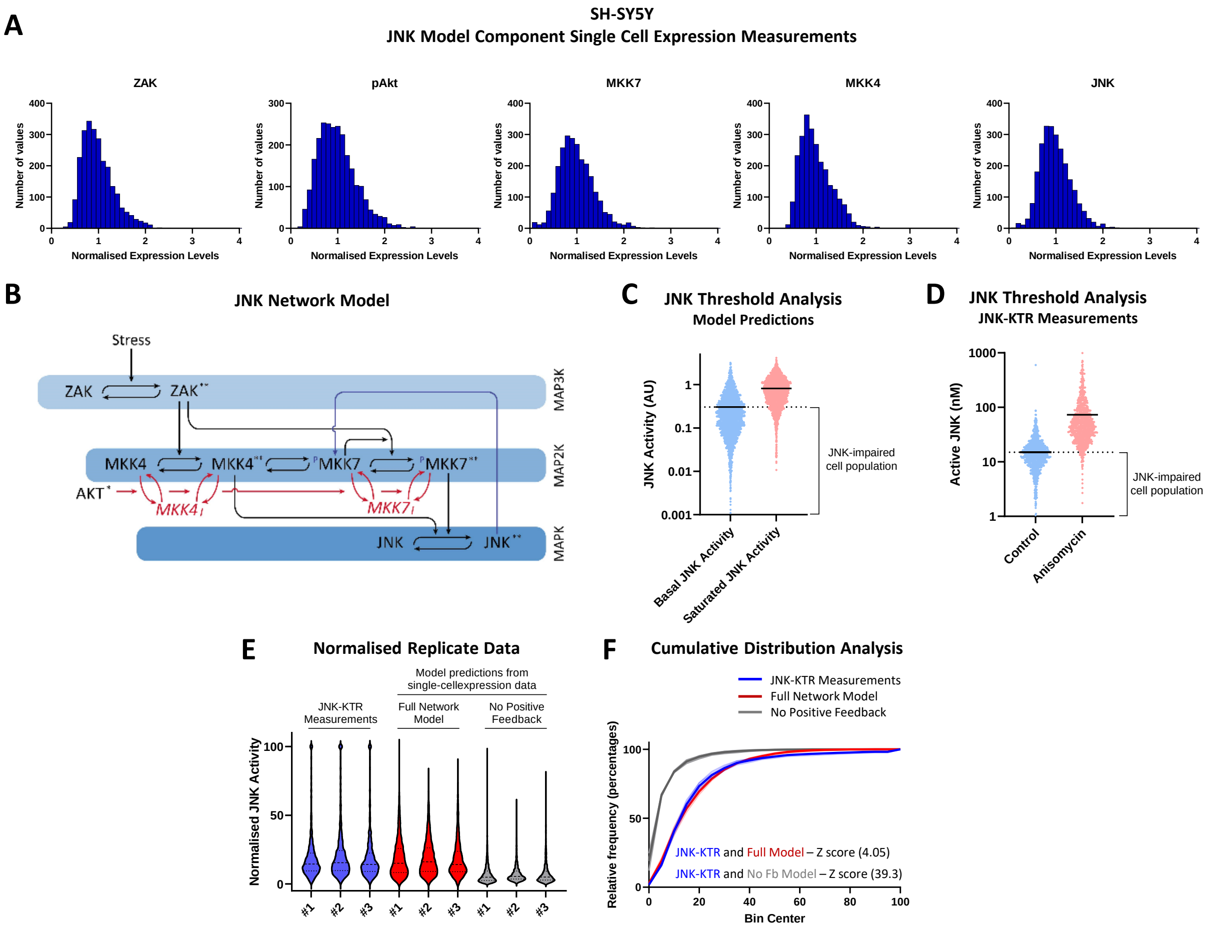

G

	Normalised JNK Network Component Measurements (Flow Cytometry)					Predicted Single Cell JNK Activity		Observed Single Cell JNK Activity
**Observed Variable**	ZAK	pAkt	MKK7	MKK4	JNK	Full network model	Model with no positive feedback	JNK-KTR measurements
**Coefficient of Variation (%)**	34.1 (± 1.1)	42.3 (± 3.5)	36.8 (± 0.6)	36.4 (± 7.1)	33.3 (± 1.1)	73.5 (± 4.5)	103 (± 1.1)	90.6 (± 1.4)

Supplementary Figure 1: Single-cell predictions and observations of JNK activity. (A) SH-SY5Y cells were fixed and permeabilised, prior to incubation with fluorescently conjugated primary antibodies (1:100) towards MKK4 (ATTO390), ZAK (Alexa 488), phospho-Akt Ser 473 (PE), JNK1/2 (Alexa 647) and MKK7 (Alexa 750). Flow cytometry analysis was performed on the FACS Symphony A5 to obtain multiplexed single-cell distributions for each model component, each normalised to its own average mean fluorescence intensity value (n=2500). **(B)** A schematic of the full JNK network model presented as a limited process graph (for clarity, the multiprotein complexes, double-phosphorylation cycles, and multiple inhibitory forms are not shown). Asterisks (**) indicate the double-phosphorylated, active forms of the kinases. Black lines indicate activating phosphorylation steps, the blue line is the positive feedback loop from JNK to MKK7 and the red lines indicate inhibitory phosphorylation from Akt. **(C)** The normalised single-cell expression values were used as parameters within this model to generate single-cell predictions of JNK activity in both a basal and saturated state (Mean, n=1000). Cells below the mean of the basal state are considered JNK-impaired. **(D)** SH-SY5Y JNK-KTR mRuby2 cells were treated with anisomycin (300 nM, 30 min) and a DMSO control prior to high-content imaging to obtain cytoplasmic:nuclear ratio values. These were converted to active JNK concentration values using a previously described model (Regot et al., 2014) (*17*) (Mean, n=2500). Cells below the mean of the control treatment are considered JNK-impaired. **(E)** Triplicate datasets of JNK-KTR measurements converted to active JNK concentrations and single-cell JNK activity predictions (full model and without the JNK to MKK7 positive feedback) were each normalised to their own maximum value (n=2500). **(F)** Cumulative distribution analysis of these triplicate datasets. **(G)** An analysis of noise within each of the individual model components and the predicted and observed measurements of JNK activity, as determined by calculation of the Coefficient of Variation.

Figure S2

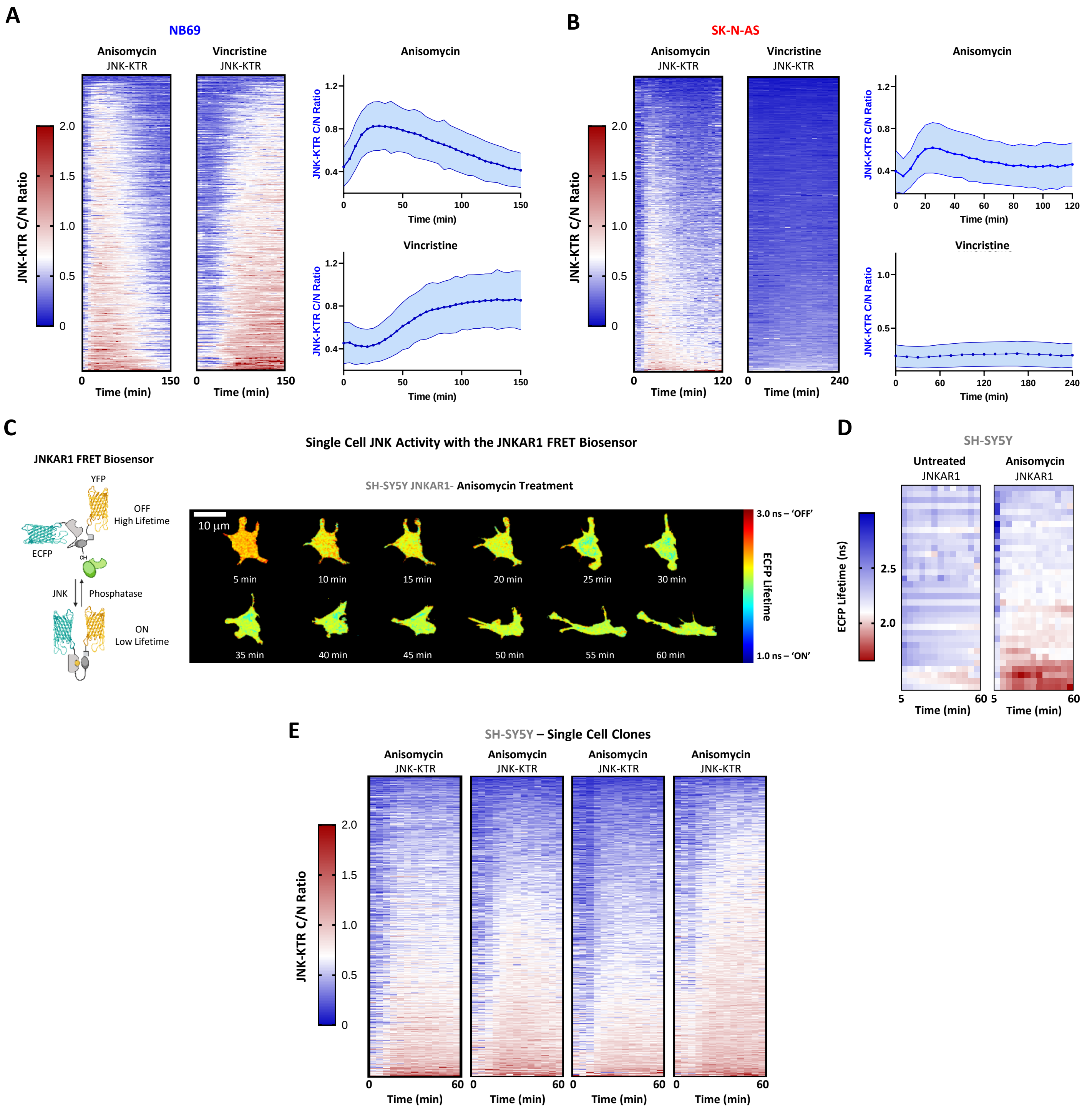

Supplementary Figure 2: Longitudinal single-cell tracking of JNK activation. (A) NB69 JNK-KTR mRuby2 cells treated with anisomycin (300 nM, n=654) or vincristine (300 nM, n=454) were imaged every 5 mins for 150 min. The cytoplasmic:nuclear ratio was calculated and values for each cell presented in rows, ranked according to the sum of all timecourse values. The population average of all JNK-KTR single-cell values is also presented (Mean ± SD). **(B)** SK-N-AS JNK-KTR mRuby2 cells were treated and imaged as in A (n=1045 – anisomycin, n=840 – vincristine). **(C)** SH-SY5Y cells were transfected with the JNKAR1 FRET biosensor and treated with anisomycin (300 nM). FLIM measurements were performed in single cells every 5 min for 1 h. The schematic image was created with BioRender.com. **(D)** FLIM measurements for control (n=34) and anisomycin (n=34) treated cells presented as a row for each cell and ranked according to the inverse sum of all timecourse values. **(E)** Single-cell clones of the SH-SY5Y JNK-KTR mRuby2 line were treated with anisomycin (300 nM, n=1500-2761) and imaged as above.

Figure S3

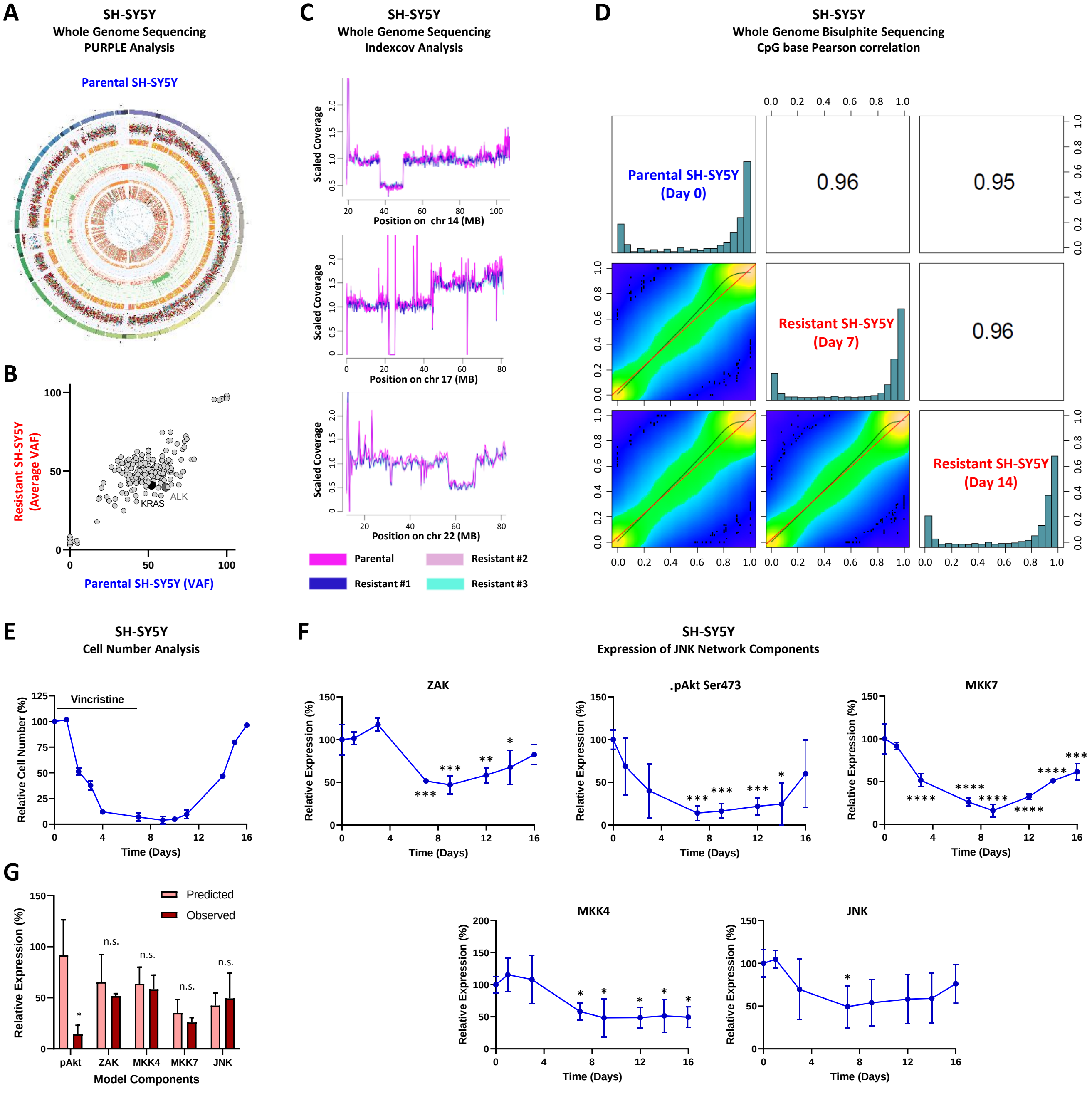

Supplementary Figure 3: Parental and resistant SH-SY5Y cell populations. (A) PURPLE analysis of whole genome sequencing performed on DNA purified from parental SH-SY5Y cells and triplicate resistant SH-SY5Y cell populations, generated by 7 days of culture in the presence of vincristine (100 nM). A CIRCOS plot is shown for the SH-SY5Y parental line. **(B)** Quantification was performed by correlating variant allele frequency (VAF) from the parental line against the average value of variants that were present in all three resistant populations. Known driver mutations in *KRAS* and *ALK* are shown in black and dark grey, respectively. **(C)** Analysis of copy number variation in these samples using Indexcov. Coverage of chromosomes 14, 17 and 22 are shown. **(D)** Pearson correlation of CpG base distribution from whole genome bisulphite sequencing performed on parental SH-SY5Y (Day 0) and resistant SH-SY5Y cell populations generated by 7 days culture in the presence of vincristine (Day 7), followed by 7 days growth in full media (Day 14). **(E)** Relative cell numbers of the SH-SY5Y line during this selection with vincristine and following removal (Mean ± SD, n=3). **(F)** Quantification of the expression of JNK network components during vincristine selection, performed on triplicate Western blots of which a representative is shown in Figure 3C (Mean ± SD, n=3. *p<0.05. **p<0.01. ***p<0.001, ****p<0.0001). **(G)** Comparison of the average expression levels of the lowest 5% of JNK-activating SH-SY5Y cells from the single cell predictions in Figure 1A (Predicted) and the surviving SH-SY5Y cells following 7 days of vincristine treatment in C (Observed).

Figure S4

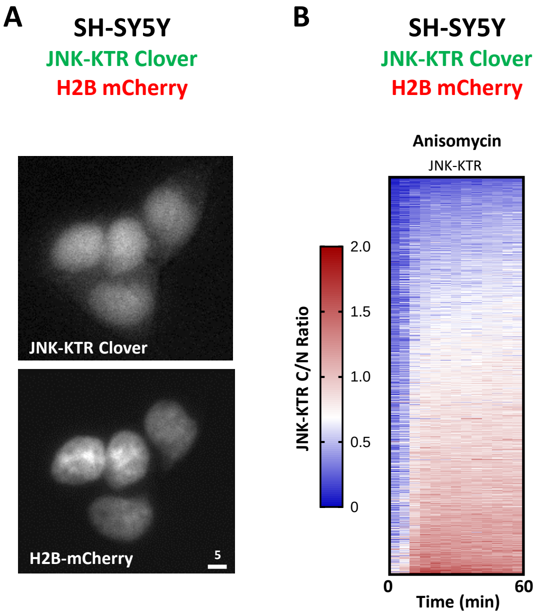

Supplementary Figure 4: Cell line generation. (A) SH-SY5Y cells were modified by lentiviral transduction to express both JNK-KTR Clover and the H2B-mCherry nuclear marker. **(B)** SH-SY5Y JNK-KTR Clover/H2B mCherry cells treated with anisomycin (300 nM, n=730) and imaged every 5 mins for 60 min. The cytoplasmic:nuclear ratio was calculated and values for each cell are presented in rows, ranked according to the sum of all timecourse values.

Figure S5

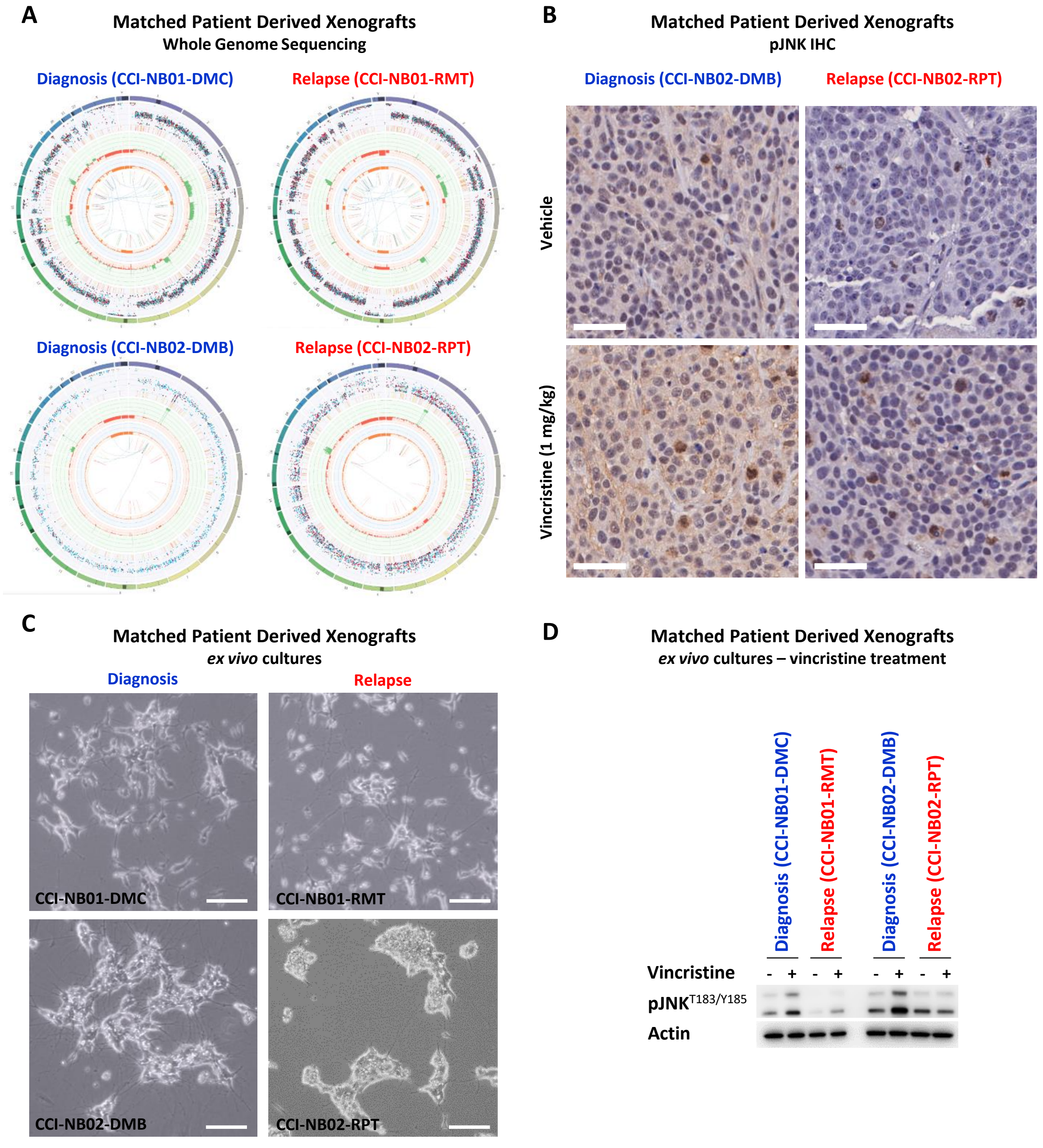

Supplementary Figure 5: Matched PDX models. (A) CIRCOS plots generated from whole genome sequencing performed on DNA extracted from each PDX model and matched patient germline samples. **(B)** Immuno-histochemistry staining for $pJNK^{T183/Y185}$ in a set of matched neuroblastoma PDX models following *in vivo* vincristine (1 mg/kg, i.v., 2 h) or vehicle treatment (n=98,104 and 73,304 for CCI-NB02-DMB. n= 75,739 and 73,655 for CCI-NB02-RPT) (Scale bar = 50 µm). **(C)** *Ex vivo* cultures of matched diagnosis and relapse PDX models. $30x10^6$ dissociated cells from each PDX model were plated onto laminin coated 15cm dishes. Following a 24 hour incubation, the neuroblastoma cells were selectively dissociated with PBS/EDTA and plated onto laminin coated plates for further experimentation (Scale bar = 50 µm). **(D)** Western blotting from lysates of *ex vivo* cultures of the matched PDX models following vincristine treatment (300 nM, 2h).

Figure S6

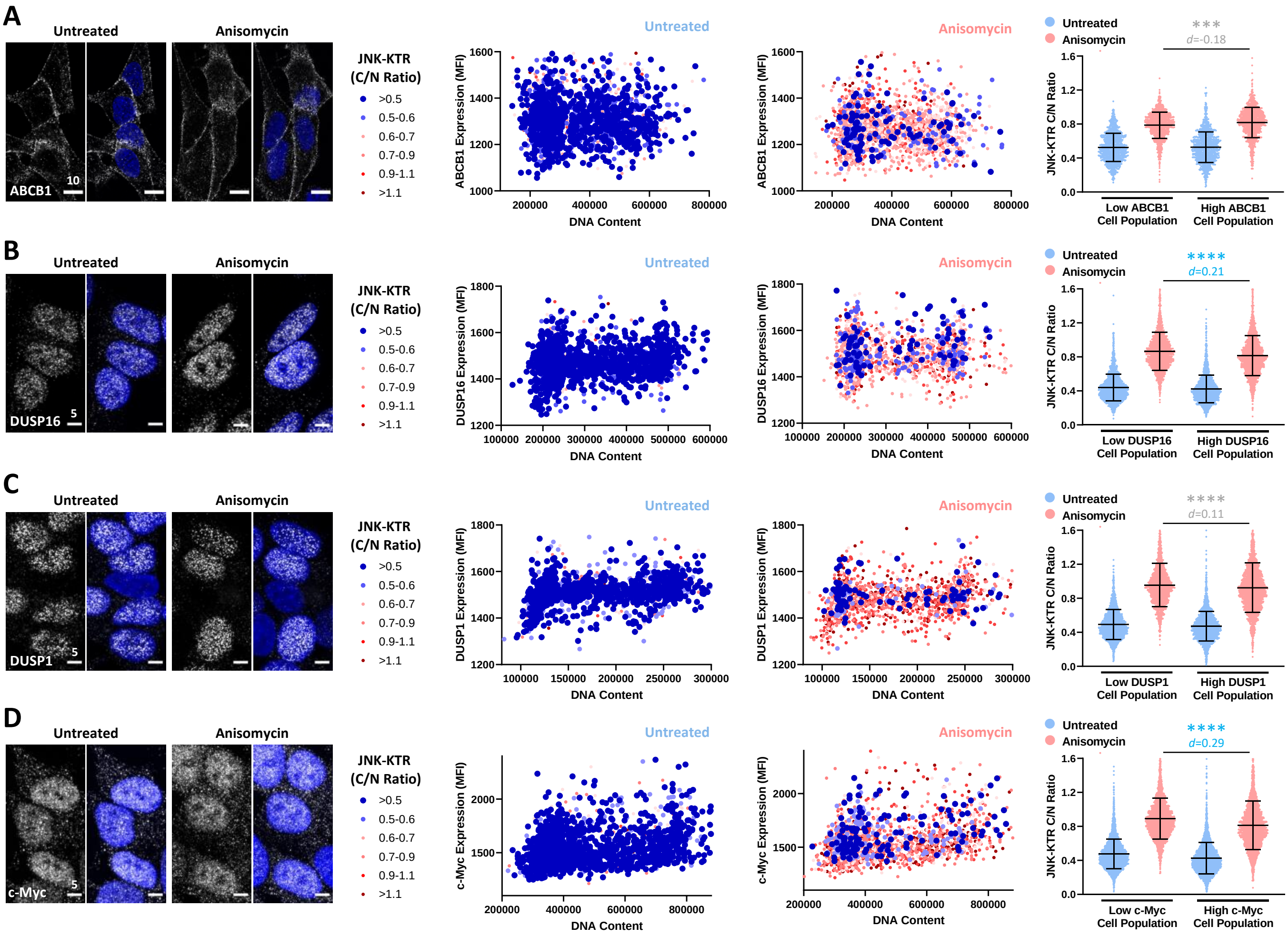

Supplementary Figure 6: Co-staining in SH-SY5Y JNK-KTR mRuby2 cells. (A) Confocal fluorescence microscopy of ABCB1 staining in SH-SY5Y JNK-KTR mRuby2 cells, with and without anisomycin stimulation (300 nM, 30 min). High-content imaging of ABCB1 staining, the JNK-KTR mRuby2 biosensor and DAPI nuclear marker. For visualisation, the JNK-KTR cytoplasmic:nuclear ratio values are displayed in the coloured bins as indicated (n=2000). Raw JNK-KTR cytoplasmic:nuclear ratio values were used for quantification based upon a median cut-off of ABCB1 staining (n=2000 - untreated, n=2000 - anisomycin). **(B)** As above, for DUSP16 antibody staining (n=4665 - untreated, n=4313 - anisomycin). **(C)** DUSP1 antibody staining (n=4984 - untreated, n=4282 - anisomycin). **(E)** c-Myc antibody staining (n=7420 - untreated, n=7188 - anisomycin). All data is mean ± SD, ****p<0.0001, ***p<0.001. Negligible effect sizes with *d*<0.20 are presented in light grey. Small effect sizes with *d*=0.2-0.5 are presented in light blue. Scale bars are 10 µm and 5 µm as indicated.

Figure S7

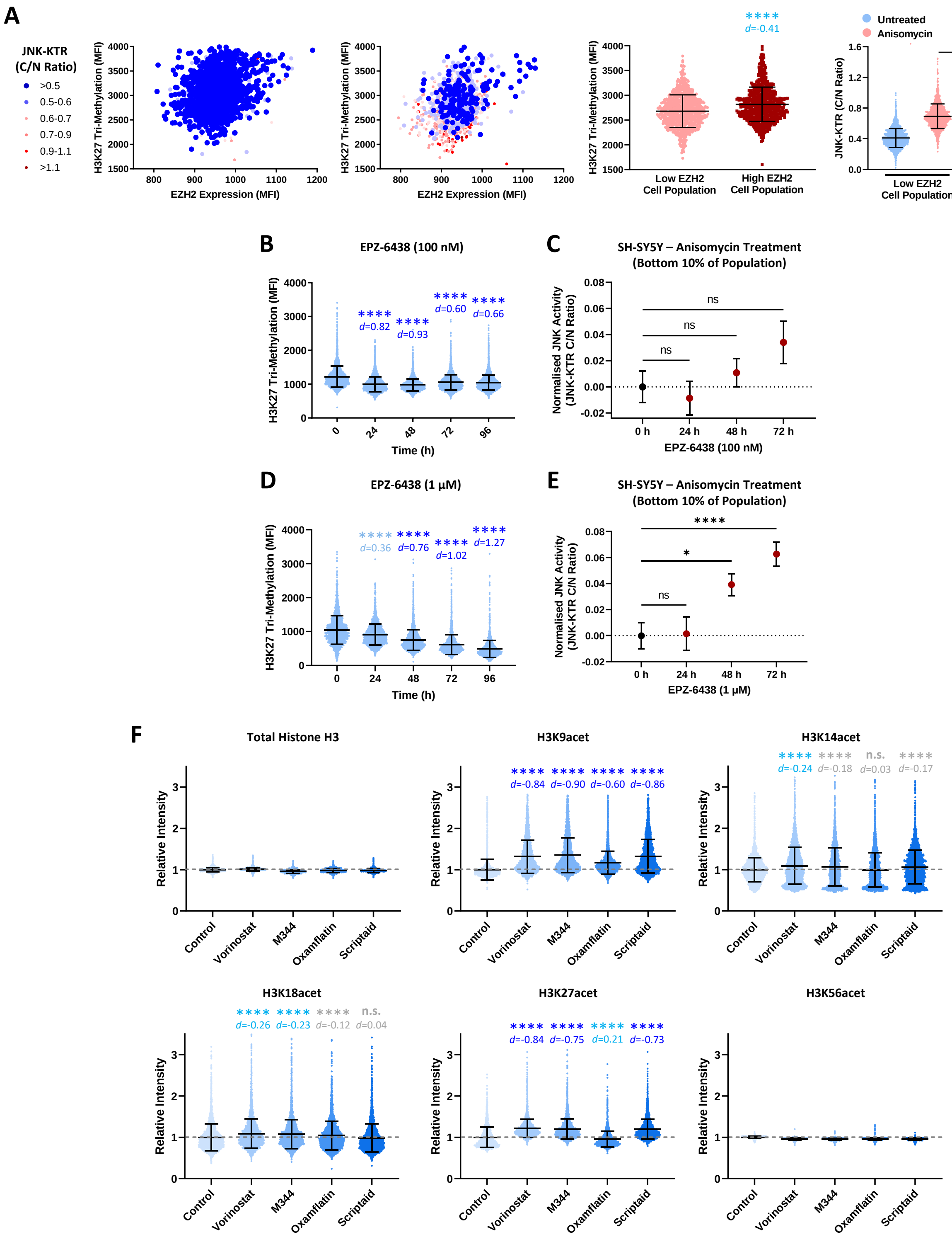

Supplementary Figure 7: High-content imaging of H3K27me3 and H3K27acet. (A) High-content imaging of SH-SY5Y JNK-KTR mRuby2 cells stained with an EZH2 antibody and DAPI nuclear marker following anisomycin stimulation (300 nM, 30 min). For visualisation, the JNK-KTR cytoplasmic:nuclear ratio values are displayed in the coloured bins as indicated (n=2000, 1440). Raw JNK-KTR cytoplasmic:nuclear ratio values were used for quantification based upon a median H3K27me3 cut-off (n=2000 - untreated, n=1440 - anisomycin). **(B,C)** SH-SY5Y JNK-KTR mRuby2 cells were pre-treated with EPZ-6438 (100 nM) for the time points indicated, prior to anisomycin stimulation (300 nM, 30 min), followed by staining with an H3K27me3 antibody (n=1000-3000). **(D,E)** SH-SY5Y JNK-KTR mRuby2 cells were pre-treated with EPZ-6438 (1 µM) for the time points indicated, prior to anisomycin stimulation (300 nM, 30 min), followed by staining with an H3K27me3 antibody (n=1000-3000). **(F)** SH-SY5Y JNK-KTR mRuby2 cells were pre-treated with the HDAC inhibitors indicated (100 nM, 24 h) prior to staining with the antibodies indicated (n>2900). All data is mean ± SD, ****p<0.0001, ***p<0.001, **p<0.01, *p<0.05. Negligible effect sizes with *d*<0.20 are presented in light grey. Small effect sizes with *d*=0.2-0.5 are presented in light blue. Large effect sizes with *d*>0.5 are presented in dark blue.)

Figure S8

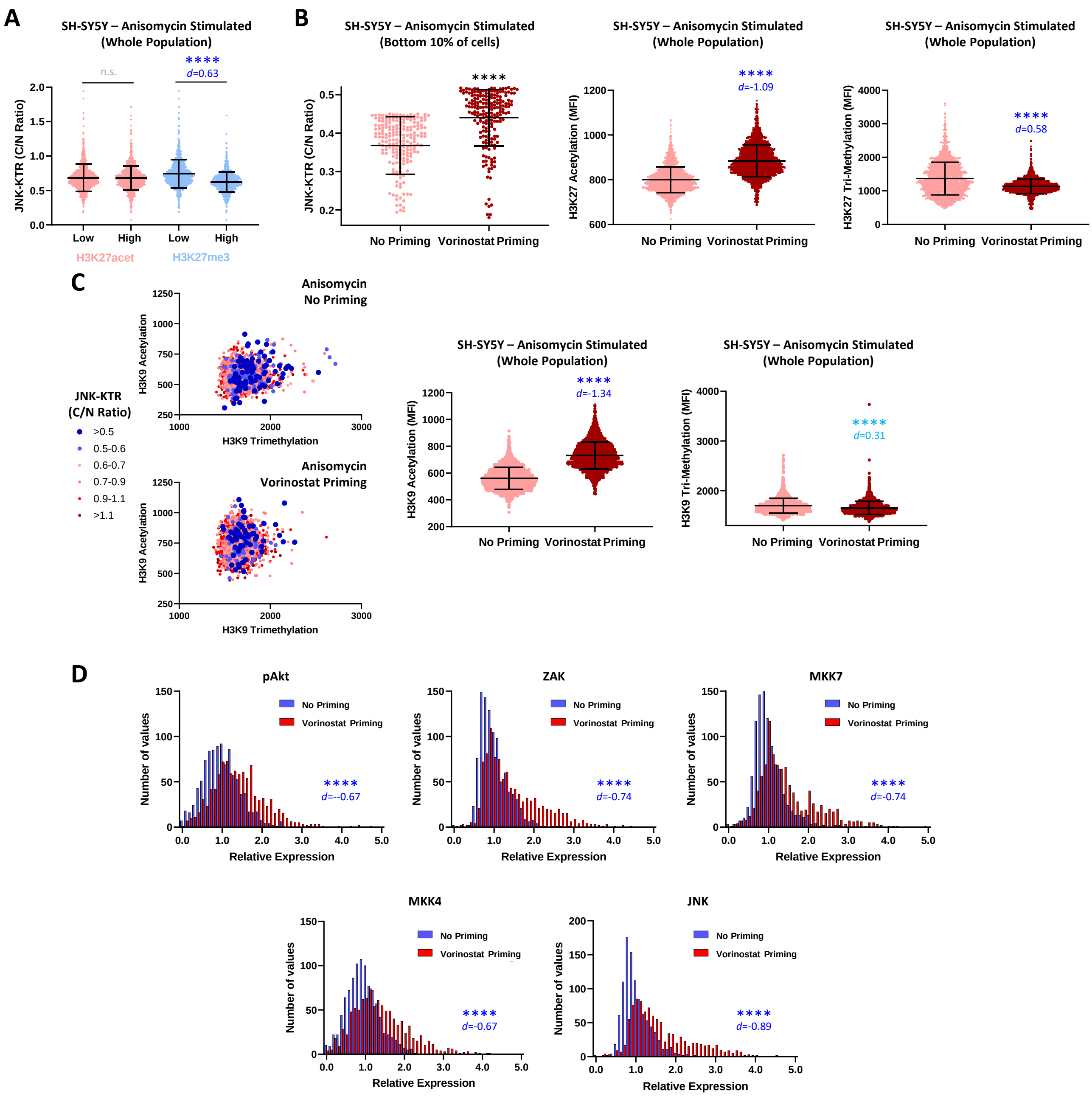

Supplementary Figure 8: Vorinostat priming in SH-SY5Y cells. (A) High-content imaging of SH-SY5Y JNK-KTR mRuby2 cells stimulated with anisomycin (300 nM, 30 min) and stained with antibodies towards H3K27acet and H3K27me3. **(B)** High-content imaging of SH-SY5Y JNK-KTR mRuby2 cells pre-treated with vorinostat (100 nM, 24 h) prior to anisomycin stimulation (300 nM, 30 min). Cells were then stained with antibodies towards H3K27me3 and H3K27acet, shown in Figure 4I. **(C)** High-content imaging of SH-SY5Y JNK-KTR mRuby2 cells pre-treated with vorinostat (100 nM, 24 h) prior to anisomycin stimulation (300 nM, 30 min). Cells were stained with an H3K9me3 antibody and an H3K9acet antibody. For visualisation, the JNK-KTR cytoplasmic:nuclear ratio values are displayed in the coloured bins as indicated (n=2000 – no priming, n=2000 – vorinostat priming). **(D)** SH-SY5Y cells were pre-treated with vorinostat (100 nM, 24 h), then fixed and permeabilised prior to incubation with fluorescently conjugated primary antibodies (1:100) towards MKK4 (ATTO390), ZAK (Alexa 488), phospho-Akt Ser 473 (PE), JNK1/2 (Alexa 647) and MKK7 (Alexa 750). Flow cytometry analysis was performed on the FACS Symphony A5 to obtain multiplexed single-cell distributions for each model component, each normalised to its own average mean fluorescence intensity value (n=1000). All data is mean ± SD, ****p<0.0001. Small effect sizes with *d*=0.2-0.5 are presented in light blue. Large effect sizes with *d*>0.5 are presented in dark blue.

Figure S9

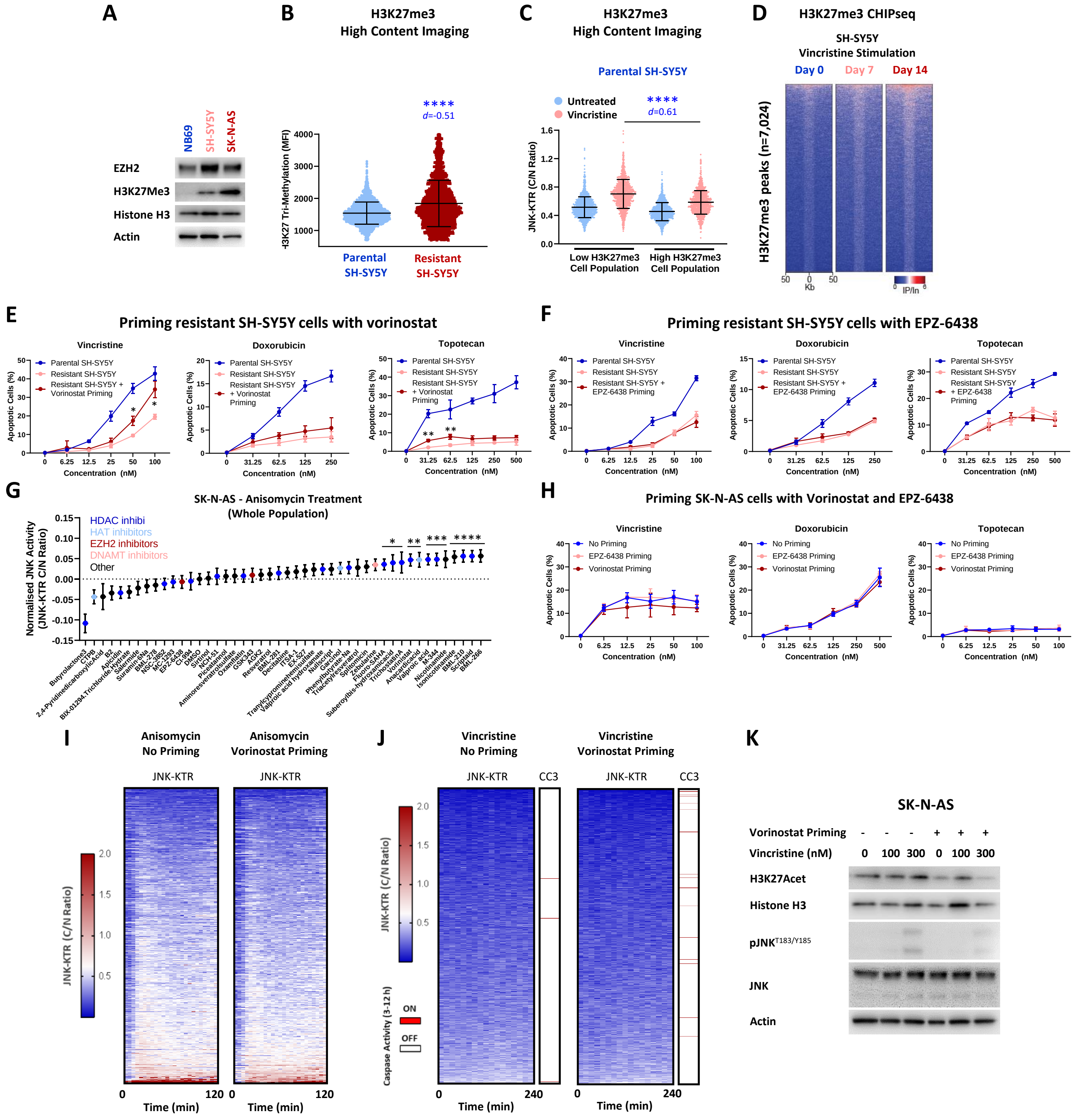

Supplementary Figure 9: H3K27me3 in resistant populations. (A) Western blotting performed with the cell lines and the antibodies indicated. **(B,C)** High content imaging of H3K27me3 antibody staining in parental and resistant SH-SY5Y JNK-KTR mRuby2 cells and parental SH-SY5Y JNK-KTR mRuby2 cells following vincristine stimulation (300 nM, 2 h) from Figure 6B. (Mean ± SD. n=2000 – untreated, n=2000 – vincristine, ****p<0.0001. Large effect sizes with *d*>0.5 are presented in dark blue. **(D)** Heatmaps of H3K27me3 ChIPseq analysis performed on chromatin purified from parental SH-SY5Y cells (Day 0) and resistant SH-SY5Y populations generated by vincristine treatment (100 nM) for 7 days (Day 7), followed by 7 days culture in full media (Day 14). **(E,F)** Quantification of apoptotic cell percentages in parental and resistant SH-SY5Y cells, pre-treated with vorinostat or EPZ-6438 (100 nM, 24 h) as indicated, prior to a further 24 h treatment with the chemotherapy drugs as indicated. High-content imaging was performed following the addition of DAPI and NucView 488, and the percentage of cells with active caspases quantified in triplicate for each condition (Mean ± SD, taken from three replicates of n=400-2700 cells, *p<0.05). **(G)** SK-N-AS JNK-KTR mRuby2 cells were pre-treated with the epigenetic modifying drugs indicated (100 nM, 24 h). Cells were then treated with anisomycin (300 nM, 30 min) before fixing, staining with DAPI and high-content imaging. JNK-KTR cytoplasmic:nuclear ratio values are displayed for the whole cell population (n=1173-1542, ****p<0.0001, ***p<0.001, **p<0.01, *p<0.05). **(H)** Quantification of apoptotic cell percentages in SK-N-AS cells treated as in E and F (Mean ± SD, taken from three replicates of n=350-1100 cells). **(I)** Longitudinal single-cell tracking of SK-N-AS JNK-KTR mRuby2 cells, pre-treated with vorinostat (100 nM, 24 h) prior to anisomycin stimulation (300 nM). Cells were imaged every 5 min for 2 h (n=631 - no priming, n=522 - vorinostat priming). **(J)** Longitudinal single-cell tracking of SK-N-AS JNK-KTR mRuby2 cells in the presence of NucView 488, pre-treated with vorinostat (100 nM, 24 h) prior to vincristine stimulation (100 nM). Cells were imaged every 15 min for 12 h (n=1216 - no priming, n=1146 - vorinostat priming). **(K)** Western blotting of lysates from SK-N-AS cells, pre-treated with vorinostat (100 nM, 24 h) then treated with vincristine at the doses indicated (2 h).

Figure S10

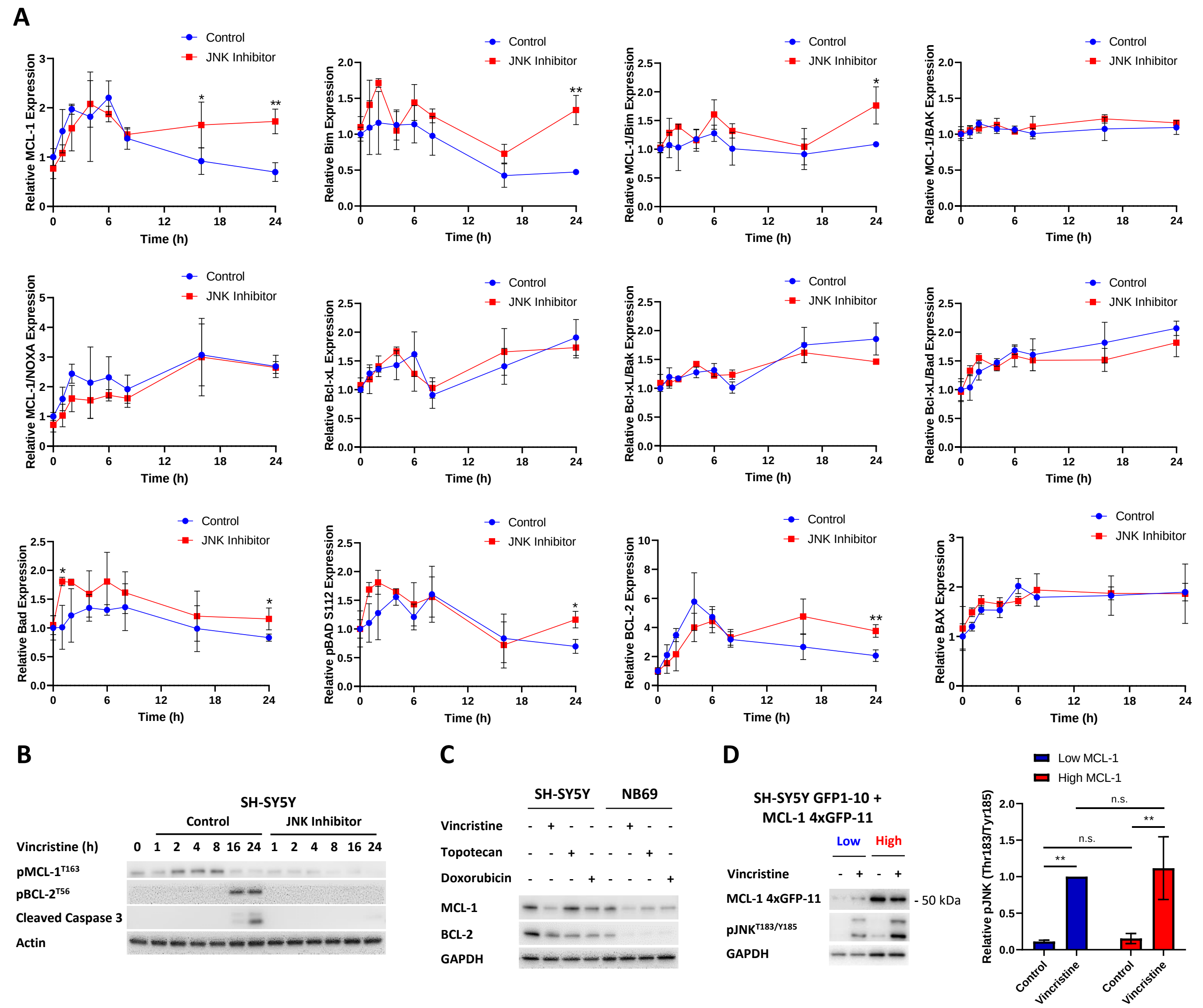

Supplementary Figure 10: JNK-mediated regulation of apoptotic mediators. (A) Quantification of the multiplex analysis in Figure 7A (Mean ± SD, n=3, **p<0.01, *p<0.05). **(B)** Western blotting of lysates from SH-SY5Y cells treated with vincristine (100 nM) for the time periods indicated. **(C)** Western blotting of lysates from SH-SY5Y and NB69 cells treated with the chemotherapy drugs indicated (100 nM, 24 h). (**D**) Western blotting of lysates from the endogenously tagged SH-SY5Y GFP1-10 + MCL-1 4xGFP-11 cell line. Cell sorting was performed to isolate the lowest and highest 5% of fluorescent cells, which were then allowed to plate down for 24 h, prior to treatment with vincristine (300 nM, 2 h). Quantification was performed from three independent replicates, with normalization performed to GAPDH (Mean ± SD, n=3, **p<0.01).

Figure S11

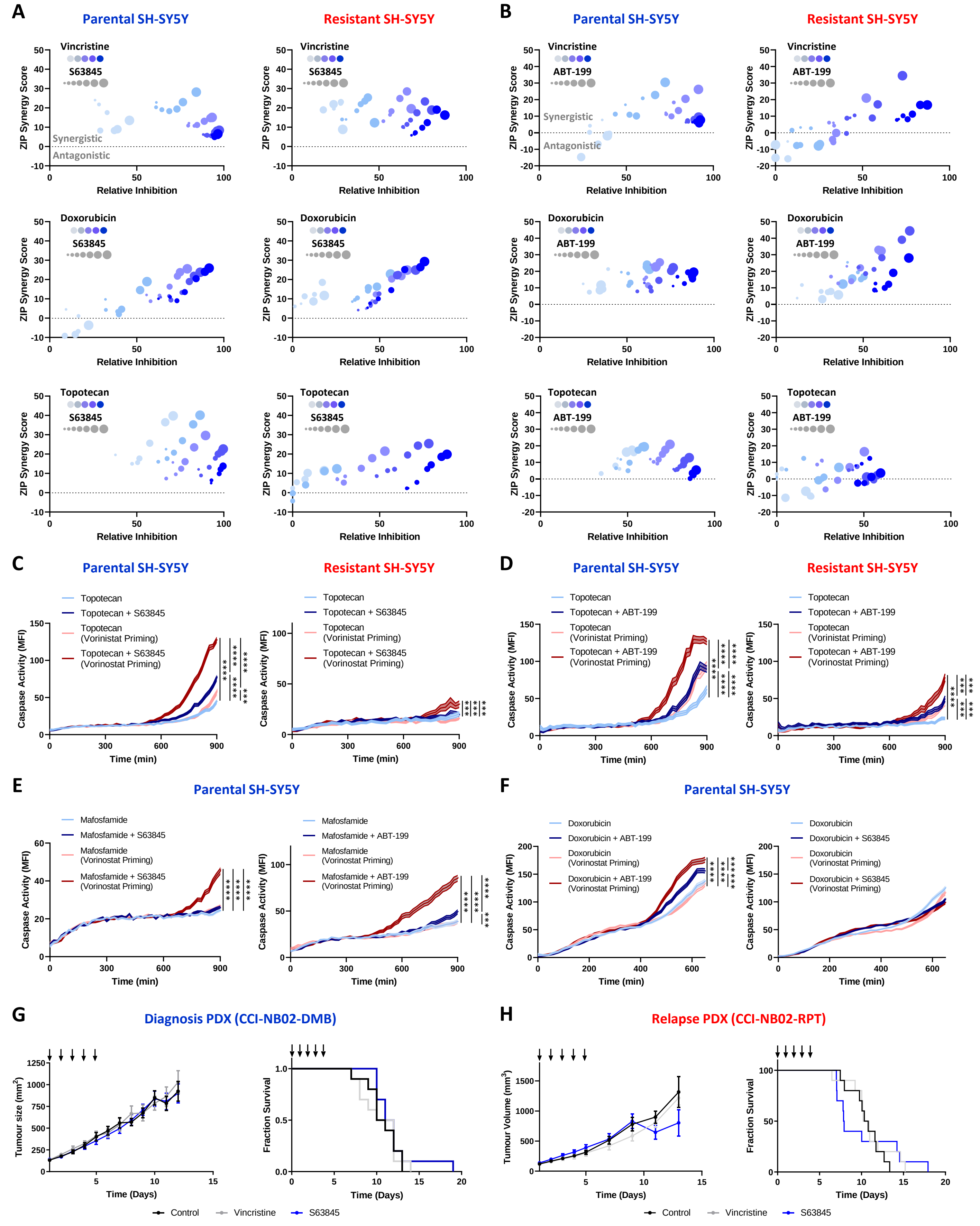

A
Parental SH-SY5Y
Resistant SH-SY5Y
B
Parental SH-SY5Y
Resistant SH-SY5Y
Vincristine
S63845
ABT-199
Doxorubicin
Topotecan
Synergistic
Antagonistic
ZIP Synergy Score
Relative Inhibition
C
Parental SH-SY5Y
Resistant SH-SY5Y
Topotecan
Topotecan + S63845
Topotecan (Vorinistat Priming)
Topotecan + S63845 (Vorinostat Priming)
D
Parental SH-SY5Y
Resistant SH-SY5Y
Topotecan + ABT-199
Topotecan + ABT-199 (Vorinostat Priming)
Caspase Activity (MFI)
Time (min)
E
Parental SH-SY5Y
Mafosfamide
Mafosfamide + S63845
Mafosfamide (Vorinostat Priming)
Mafosfamide + S63845 (Vorinostat Priming)
Mafosfamide + ABT-199
Mafosfamide + ABT-199 (Vorinostat Priming)
F
Parental SH-SY5Y
Doxorubicin
Doxorubicin + ABT-199
Doxorubicin (Vorinostat Priming)
Doxorubicin + ABT-199 (Vorinostat Priming)
Doxorubicin + S63845
Doxorubicin + S63845 (Vorinostat Priming)
G
Diagnosis PDX (CCI-NB02-DMB)
Tumour size (mm³)
Fraction Survival
Time (Days)
Control
Vincristine
S63845
H
Relapse PDX (CCI-NB02-RPT)
Tumour Volume (mm³)
Fraction Survival
Time (Days)
Control
Vincristine
S63845

Supplementary Figure 11: Synergy with BH3 mimetics. (A,B) Synergy analysis of the cytotoxicity assays performed in parental and resistant SH-SY5Y cell populations in Figure 7G,H. Cells were treated with either vincristine (0-100 nM), doxorubicin (0-500 nM) or topotecan (0-500 nM) in the presence of S63845 (0-1000 nM) or ABT-199 (0-1000 nM) (Mean. n=6). Quantification of synergy was performed with SynergyFinder 2.0. Values lower than 0 represent and antagonistic interaction, values higher than 0 a synergistic interaction. Increasing doses of chemotherapy are represented by darker shades of blue for each data point, while increasing doses of S63845/ABT-199 are represented by an increase in size. **(C)** Longitudinal high-content imaging of parental and resistant SH-SY5Y cells in the presence of NucView 488, pre-treated with vorinostat (100 nM) or a DMSO control, then treated with either topotecan (100 nM) or topotecan and S63845 (100 nM and 250 nM, respectively) (Mean ± 95% CI. n=900-2010). **(D)** Longitudinal high content imaging of parental and resistant SH-SY5Y cells in the presence of NucView 488 as in C, with the use of ABT-199 instead of S63845 (Mean ± 95% CI. n=1600-4500). **(E)** Longitudinal high content imaging of parental SH-SY5Y cells in the presence of NucView 488 as in C and D, with the use of mafosfamide (3 µM) (Mean ± 95% CI. n=1600-5000). (**F**) Longitudinal high content imaging of parental SH-SY5Y cells in the presence of NucView 488 as in C and D, with the use of doxorubicin (500 nM) (Mean ± 95% CI. n=1500-2300). **(G)** NSG mice were implanted with $1x10^6$ CCI-NB02-DMB cells, once tumours reached 100 mm^3 the mice were treated with either vincristine (0.2 mg/kg, i.v.), S63845 (25 mg/kg, i.v.) or relevant vehicle controls, once daily for 5 days. Tumour growth was measured every day until ethical endpoint (1000 mm^3) (Mean ± SEM. n=8). **(H)** NSG mice were implanted with $1x10^6$ CCI-NB02-RPT cells, once tumours reached 100 mm^3 the mice were treated as in C.

Data File S1: Copy number analysis of parental and resistant SH-SY5Y cell populations.

Table S1: Analysis of established driver mutations within matched PDX models

Patient ID	Clinical Stage	Copy Number Variation	Single Nucleotide Variation (VAF)	Structural Variation	Mutation Burden
**CCI-NB01**	Diagnosis<br>(CCI-NB01-DMC)	Chr4 gain<br>Chr14 gain<br>CDKN2A/B bi-allelic deletion	MYCN: c.131C>T, p.Pro44Leu (0.70)<br>NF1 c.6006+1del (1)	TERT disruption	3 mutations/Mb
	Relapse<br>(CCI-NB01-RMT)	ChrY loss<br>CDKN2A/B bi-allelic deletion	MYCN: c.131C>T, p.Pro44Leu (0.65)<br>NF1: c.6006+1del (1)	TERT disruption	5 mutations/Mb
**CCI-NB02**	Diagnosis<br>(CCI-NB02-DMB)	MYCN amplification<br>ALK amplification	MYCN: c.485A>C, p.His162Pro (0.03)<br>MYCN: c.524T>G, p.Leu175Arg (0.02)<br>MYCN: c.542A>C, p.His181Pro (0.03)<br>MYCN: c.577T>G, p.Phe193Val (0.02)<br>MYCN: c.583T>G, p.Phe195Val (0.03)<br>MYCN: c.584T>G, p.Phe195Cys (0.03)<br>ALK: c.2762T>C, p.Phe921Ser (0.05)<br>ALK: c.2754A>C, p.Arg918Ser (0.17)<br>SETD2: c.5752G>T, p.Glu1918Ter (0.47)	GACAT3-ALK (inv)	0.72 mutations/Mb
	Relapse<br>(CCI-NB02-RPT)	MYCN amplification<br>ALK amplification	MYCN: c.485A>C, p.His162Pro (0.03)<br>MYCN: c.524T>A, p.Leu175Gln (0.04)<br>MYCN: c.577T>G, p.Phe193Val (0.03)<br>MYCN: c.584T>G, p.Phe195Cys (0.03)<br>MYCN: c.596A>C, p.Lys199Thr (0.02)<br>MYCN: c.733A>C, p.Ser245Arg (0.02)<br>ALK: c.2762T>C, p.Phe921Ser (0.05)	GACAT3-ALK (inv)	1.97 mutations/Mb

Supplementary Table 2 – Reagent details

REAGENT or RESOURCE	SOURCE	IDENTIFIER
**Antibodies**		
α-JNK	Cell Signaling	Cat# 9252
α-phospho-JNK T183/Y185	Cell Signaling	Cat# 9251 (Western Blotting)
α-phospho-JNK T183/Y185	R&D Systems	Cat# AF1205 (Immunohistochemistry)
α-Akt	Cell Signaling	Cat# 9272
α-phospho-Akt S473	Cell Signaling	Cat# 4060
α-Cleaved Caspase 3	Cell Signaling	Cat# 9661
α-ABCB1/MDR1	Cell Signaling	Cat# 13342
α-DUSP1/MKP1	Cell Signaling	Cat# 48625
α-c-Myc	Cell Signaling	Cat# 5605
α-N-Myc	Cell Signaling	Cat# 84406
α-EZH2	Cell Signaling	Cat# 5246
α-MCL-1	Cell Signaling	Cat# 4572
α-phospho-MCL-1 T163	Cell Signaling	Cat# 14765
α-BCL-2	Cell Signaling	Cat# 2870

REAGENT or RESOURCE	SOURCE	IDENTIFIER
α-phospho-BCL-2 T56	Cell Signaling	Cat# 2875
α-Tri-Methyl-Histone H3 (Lys27)	Cell Signaling	Cat# 9733
α-Acetyl-Histone H3 (Lys27)	Cell Signaling	Cat# 8173
α-Tri-Methyl-Histone H3 (Lys9)	Cell Signaling	Cat# 13969
α-Acetyl-Histone H3 (Lys9)	Cell Signaling	Cat# 9683
Acetyl-Histone H3 Antibody Sampler Kit (Lys 9, 14, 18, 27, 56 and Total Histone H3)	Cell Signaling	Cat# 9927
α-MKK4	Abcam	Cat# ab33912
α-ZAK	Abcam	Cat# ab65249
α- DUSP16	ThermoFisher Scientific	Cat# PA5-29888
α-Actin (AC-15)	Merck	Cat# A1978
α-MKK7	Acris Antibodies	Cat# AM00096PU-N
α-phospho-Akt (S473) PE-conjugated	Cell Signaling	Cat# 5315

REAGENT or RESOURCE	SOURCE	IDENTIFIER
α-ZAK Alexa 488 conjugated	Bioss	Cat# bs-13546R-A488
α-JNK Alexa 647 conjugated	Bioss	Cat# bs-2592R-A647
α-MKK7 Alexa 750 conjugated	Bioss	Cat# bs-1979R-A750
α-MKK4 ATTO390	StressMarq Biosciences	Cat# SPC-1173-A390
α-Acetyl-Histone H3 (Lys27) Alexa 488 conjugated	Cell Signaling	Cat# 15485
α-Acetyl-Histone H3 (Lys9) Alexa 488 conjugated	Cell Signaling	Cat# 9683
α-Tri-Methyl-Histone H3 (Lys27) Alexa 647 conjugated	Cell Signaling	Cat# 12158
**Experimental Models: Organisms/Strains**		
BALB/c-Fox1nuAusbJ	Australian BioResources	
NOD.Cg-Prkdc<scid>IL2rg<tm1Wjl>/SzJAusb	Australian BioResources	
**Plasmids**		
Plasmid: pLentiPGK Blast	31	Cat# 59154; RRID: Addgene_59154

REAGENT or RESOURCE	SOURCE	IDENTIFIER
DEST JNKKTRmRuby2		
Plasmid: pLentiPGK Puro DEST JNKKTRClover	[31]	Cat# 59151; RRID: Addgene_59151
Plasmid: pcDNA3-JNKAR1	[32]	Cat #61625; RRID: Addgene_61625
**Chemicals**		
Vorinostat	Selleck Chemicals	Cat# S1047
EPZ-6438	Selleck Chemicals	Cat# S7128
GSK-343	Selleck Chemicals	Cat# S7164
ABT-199	Selleck Chemicals	Cat# S8048
JNK-IN-8	Selleck Chemicals	Cat# S4901
Anisomycin	Sigma-Aldrich	Cat# A9789
Vincristine	Sigma-Aldrich	Cat# V8388
S63845	MedChemExpress	Cat# HY-100741
Hoechst 33342	Thermo Fisher Scientific	Cat# 62249
**Oligonucleotides**		

REAGENT or RESOURCE	SOURCE	IDENTIFIER
4× GFP11 dsDNA donor templates N-Terminal	Genscript	ATGAGAGACCACATGGTTTTGCATGAGTATGTGAACGCGGCGGGTATAACTGGTGGGTCGGGCGGACGAGACCATATGGTGCTTCACGAATACGTAAACGCAGCTGGCATTACTGGCGGATCAGGTGGCAGGGATCACATGGTACTCCATGAGTACGTGAACGCTGCTGGAATCACAGGCGGTAGCGGCGGTCGGGACCATATGGTCCTGCACGAATATGTCAATGCTGCCGGTATCACCGGAAGTTCCGGCGGCTAG
Primer for HDR PCR amplification – MCL-1 Forward Primer (lowercase: gene specific; uppercase: donor specific)	Integrated DNA Technologies	ggcggcggcggcgactggcaatgtttggcctcaaaAGAGACCACATGGTTTTGCAT
Primer for HDR PCR amplification – MCL-1 Reverse Primer (lowercase: gene specific; uppercase: donor specific)	Integrated DNA Technologies	ttgagtccgattaccgcgtttcttttgaggccaaaGCCGCCGGAACTTCCGGTGAT
4× GFP11 HDR template		ggcggcggcggcgactggcaatgtttggcctcaaaAGAGACCACATGGTTTTGCATGAGTATGTGAACGCGGCGGGTATAACTGGTGGGTCGGGCGGACGAGACCATATGGTGCTTCACGAATACGTAAACGCAGCTGGCATTACTGGCGGATCAGGTGGCAGGGATCACATGGTACTCCATGAGTACGTGAACGCTGCTGGAATCACAGGCGGTAGCGGCGGTCGGGACCATATGGTCCTGCACGAATATGTCAATGCTGCCGGTATCACCGGAAGTTCCGGCGGCtttggcctcaaaagaaacgcggtaatcggactcaa

REAGENT or RESOURCE	SOURCE	IDENTIFIER
sgRNA sequence – MCL-1	Integrated DNA Technologies	GATTACCGCGTTTCTTTTG
**Deposited Data**		
Whole genome sequencing datasets	European Genome-Phenome Archive	EGAS00001005811
Whole genome bisulphite sequencing datasets	ArrayExpress database	E-MTAB-11223
H3K27me3 ChIPseq datasets	ArrayExpress database	E-MTAB-11219
**Software and Algorithms**		
Ordinary Different Equation based JNK Network Model	(Fey et al., 2015)	
