# Supplementary material for: Memory of stochastic single-cell apoptotic signaling promotes chemoresistance in neuroblastoma
Source: Sci Adv. 2023 Mar 3;9(9):eabp8314. doi: 10.1126/sciadv.abp8314 (PMC9984174; doi:10.1126/sciadv.abp8314)
Supplement: Supplementary file 2 — Data file S1 [file sciadv.abp8314_data_file_s1.zip › SHSY5Y-indexcov-depth-1.html]

---

back to index
