# Supplementary material for: Memory of stochastic single-cell apoptotic signaling promotes chemoresistance in neuroblastoma
Source: Sci Adv. 2023 Mar 3;9(9):eabp8314. doi: 10.1126/sciadv.abp8314 (PMC9984174; doi:10.1126/sciadv.abp8314)
Supplement: Supplementary file 2 — Data file S1 [file sciadv.abp8314_data_file_s1.zip › SHSY5Y-indexcov.html]

SHSY5Y:indexcov


This is the indexcov summary page created with version 0.1.16.  
Click the ? above each plot for help describing that type of plot.

|  |  |  |
| --- | --- | --- |
| Inferred sex ? | Mapped Counts ? | Bin Counts ? |

---

Pedigree File

contains inferred sex, bins counts, and PCA values used to make the above plots

SHSY5Y-indexcov.ped

Coverage BED File

contains scaled coverage for every sample (each column) for each 16,384 interval in the index

SHSY5Y-indexcov.bed.gz

---
