# Supplementary figures and images for: Memory of stochastic single-cell apoptotic signaling promotes chemoresistance in neuroblastoma
Source: Sci Adv. 2023 Mar 3;9(9):eabp8314. doi: 10.1126/sciadv.abp8314 (PMC9984174; doi:10.1126/sciadv.abp8314)

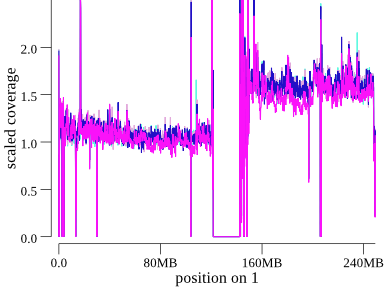

Supplement: Supplementary file 2 — Data file S1 [file sciadv.abp8314_data_file_s1.zip › SHSY5Y-indexcov-depth-1.png]

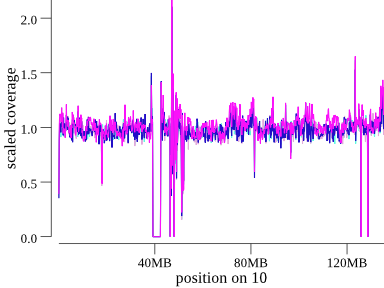

Supplement: Supplementary file 2 — Data file S1 [file sciadv.abp8314_data_file_s1.zip › SHSY5Y-indexcov-depth-10.png]

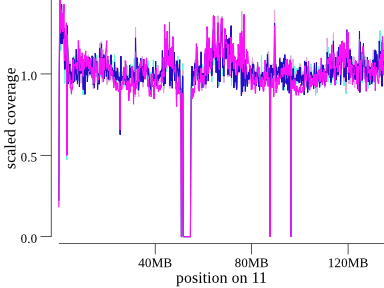

Supplement: Supplementary file 2 — Data file S1 [file sciadv.abp8314_data_file_s1.zip › SHSY5Y-indexcov-depth-11.png]

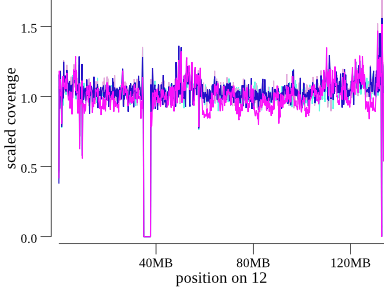

Supplement: Supplementary file 2 — Data file S1 [file sciadv.abp8314_data_file_s1.zip › SHSY5Y-indexcov-depth-12.png]

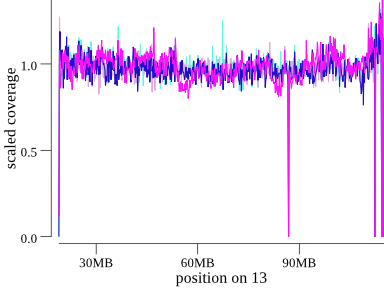

Supplement: Supplementary file 2 — Data file S1 [file sciadv.abp8314_data_file_s1.zip › SHSY5Y-indexcov-depth-13.png]

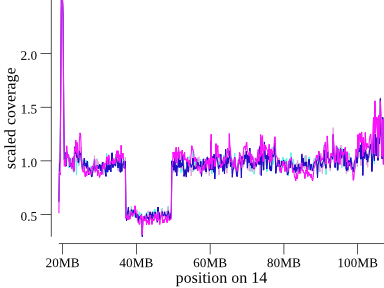

Supplement: Supplementary file 2 — Data file S1 [file sciadv.abp8314_data_file_s1.zip › SHSY5Y-indexcov-depth-14.png]

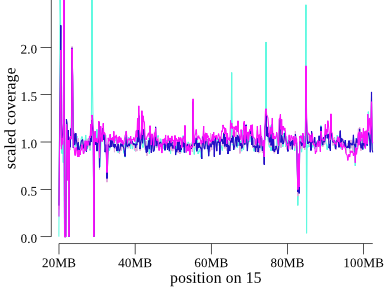

Supplement: Supplementary file 2 — Data file S1 [file sciadv.abp8314_data_file_s1.zip › SHSY5Y-indexcov-depth-15.png]

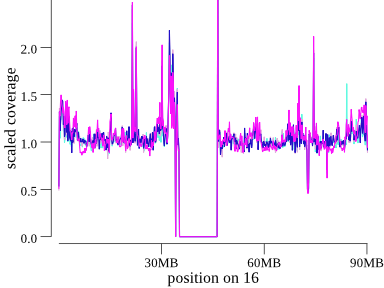

Supplement: Supplementary file 2 — Data file S1 [file sciadv.abp8314_data_file_s1.zip › SHSY5Y-indexcov-depth-16.png]

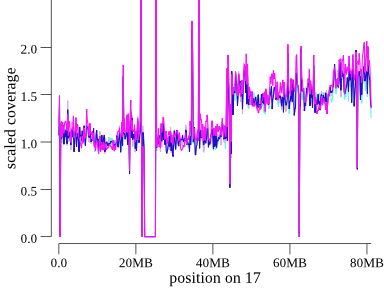

Supplement: Supplementary file 2 — Data file S1 [file sciadv.abp8314_data_file_s1.zip › SHSY5Y-indexcov-depth-17.png]

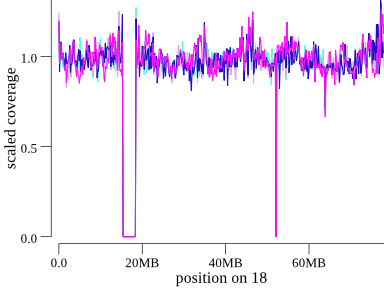

Supplement: Supplementary file 2 — Data file S1 [file sciadv.abp8314_data_file_s1.zip › SHSY5Y-indexcov-depth-18.png]

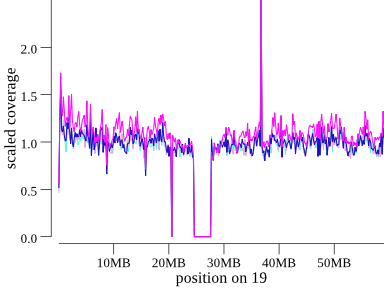

Supplement: Supplementary file 2 — Data file S1 [file sciadv.abp8314_data_file_s1.zip › SHSY5Y-indexcov-depth-19.png]

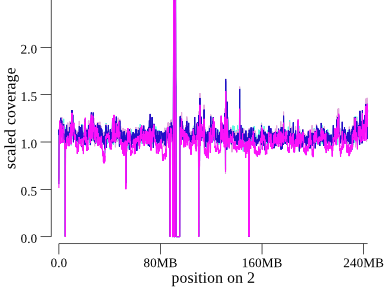

Supplement: Supplementary file 2 — Data file S1 [file sciadv.abp8314_data_file_s1.zip › SHSY5Y-indexcov-depth-2.png]

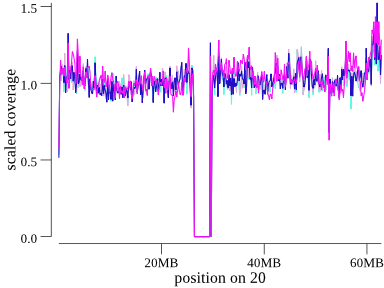

Supplement: Supplementary file 2 — Data file S1 [file sciadv.abp8314_data_file_s1.zip › SHSY5Y-indexcov-depth-20.png]

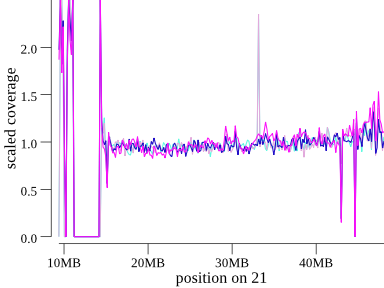

Supplement: Supplementary file 2 — Data file S1 [file sciadv.abp8314_data_file_s1.zip › SHSY5Y-indexcov-depth-21.png]

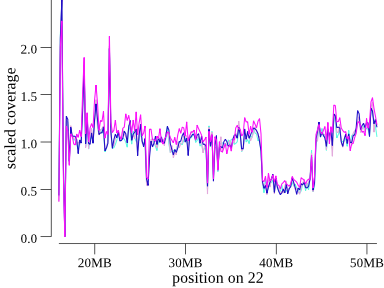

Supplement: Supplementary file 2 — Data file S1 [file sciadv.abp8314_data_file_s1.zip › SHSY5Y-indexcov-depth-22.png]

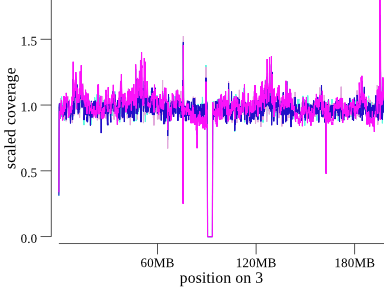

Supplement: Supplementary file 2 — Data file S1 [file sciadv.abp8314_data_file_s1.zip › SHSY5Y-indexcov-depth-3.png]

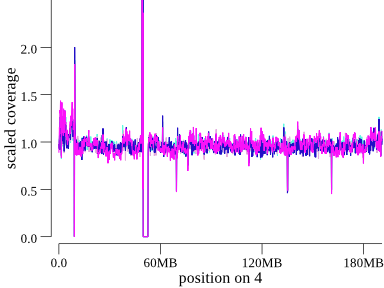

Supplement: Supplementary file 2 — Data file S1 [file sciadv.abp8314_data_file_s1.zip › SHSY5Y-indexcov-depth-4.png]

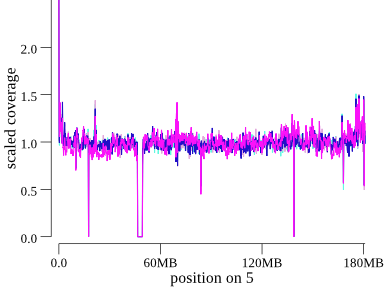

Supplement: Supplementary file 2 — Data file S1 [file sciadv.abp8314_data_file_s1.zip › SHSY5Y-indexcov-depth-5.png]

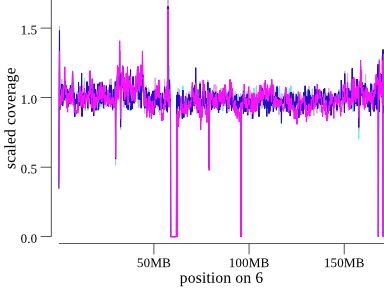

Supplement: Supplementary file 2 — Data file S1 [file sciadv.abp8314_data_file_s1.zip › SHSY5Y-indexcov-depth-6.png]

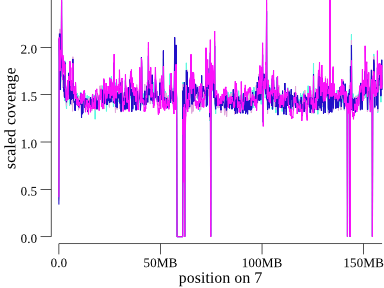

Supplement: Supplementary file 2 — Data file S1 [file sciadv.abp8314_data_file_s1.zip › SHSY5Y-indexcov-depth-7.png]

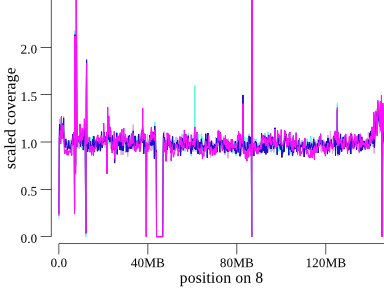

Supplement: Supplementary file 2 — Data file S1 [file sciadv.abp8314_data_file_s1.zip › SHSY5Y-indexcov-depth-8.png]

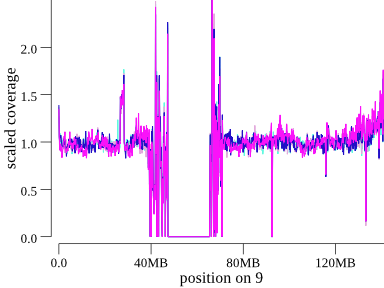

Supplement: Supplementary file 2 — Data file S1 [file sciadv.abp8314_data_file_s1.zip › SHSY5Y-indexcov-depth-9.png]

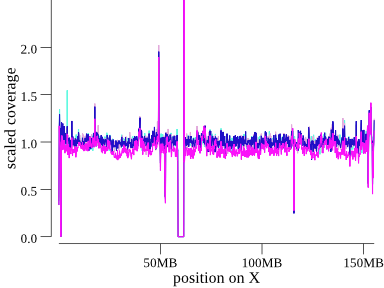

Supplement: Supplementary file 2 — Data file S1 [file sciadv.abp8314_data_file_s1.zip › SHSY5Y-indexcov-depth-X.png]

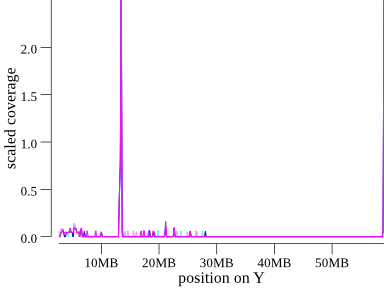

Supplement: Supplementary file 2 — Data file S1 [file sciadv.abp8314_data_file_s1.zip › SHSY5Y-indexcov-depth-Y.png]

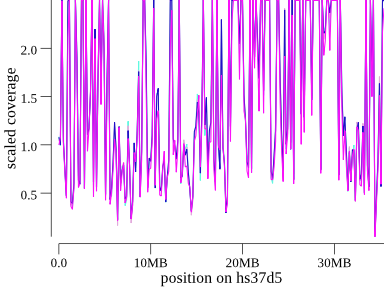

Supplement: Supplementary file 2 — Data file S1 [file sciadv.abp8314_data_file_s1.zip › SHSY5Y-indexcov-depth-hs37d5.png]

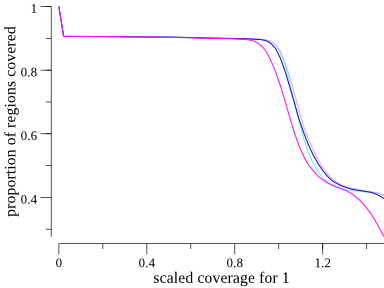

Supplement: Supplementary file 2 — Data file S1 [file sciadv.abp8314_data_file_s1.zip › SHSY5Y-indexcov-roc-1.png]

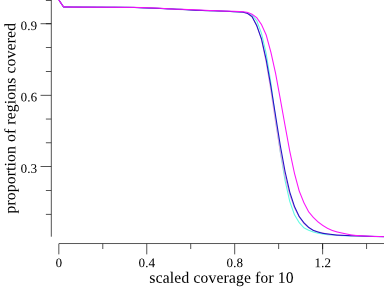

Supplement: Supplementary file 2 — Data file S1 [file sciadv.abp8314_data_file_s1.zip › SHSY5Y-indexcov-roc-10.png]

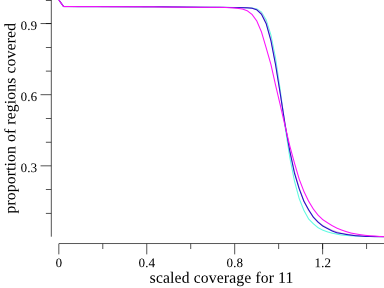

Supplement: Supplementary file 2 — Data file S1 [file sciadv.abp8314_data_file_s1.zip › SHSY5Y-indexcov-roc-11.png]

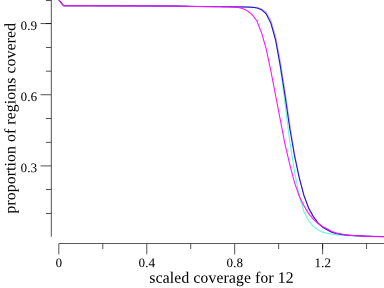

Supplement: Supplementary file 2 — Data file S1 [file sciadv.abp8314_data_file_s1.zip › SHSY5Y-indexcov-roc-12.png]

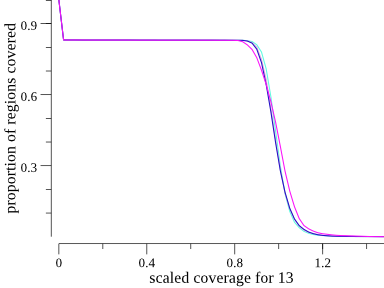

Supplement: Supplementary file 2 — Data file S1 [file sciadv.abp8314_data_file_s1.zip › SHSY5Y-indexcov-roc-13.png]

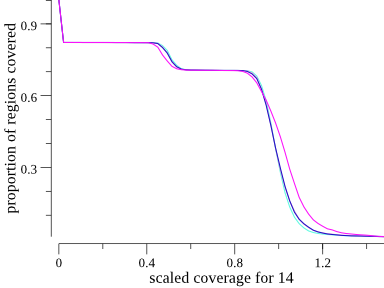

Supplement: Supplementary file 2 — Data file S1 [file sciadv.abp8314_data_file_s1.zip › SHSY5Y-indexcov-roc-14.png]

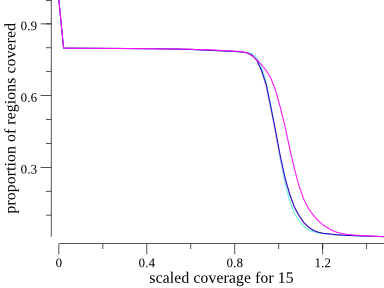

Supplement: Supplementary file 2 — Data file S1 [file sciadv.abp8314_data_file_s1.zip › SHSY5Y-indexcov-roc-15.png]

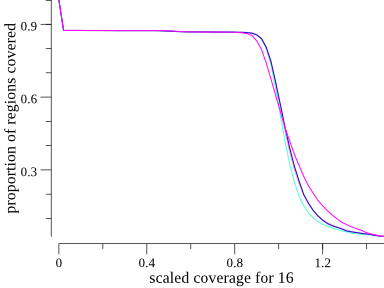

Supplement: Supplementary file 2 — Data file S1 [file sciadv.abp8314_data_file_s1.zip › SHSY5Y-indexcov-roc-16.png]

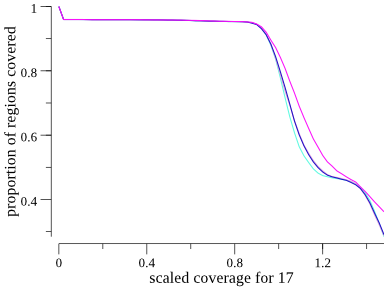

Supplement: Supplementary file 2 — Data file S1 [file sciadv.abp8314_data_file_s1.zip › SHSY5Y-indexcov-roc-17.png]

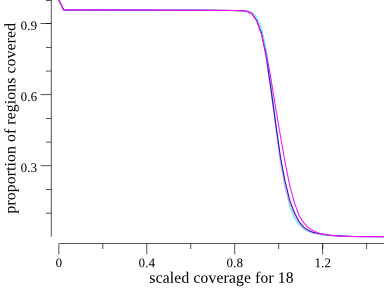

Supplement: Supplementary file 2 — Data file S1 [file sciadv.abp8314_data_file_s1.zip › SHSY5Y-indexcov-roc-18.png]

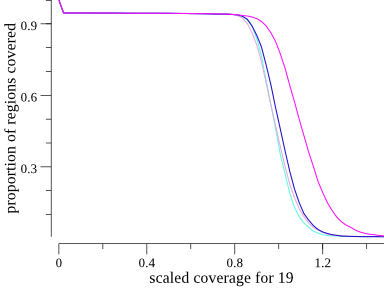

Supplement: Supplementary file 2 — Data file S1 [file sciadv.abp8314_data_file_s1.zip › SHSY5Y-indexcov-roc-19.png]

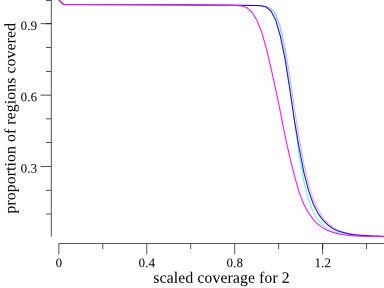

Supplement: Supplementary file 2 — Data file S1 [file sciadv.abp8314_data_file_s1.zip › SHSY5Y-indexcov-roc-2.png]

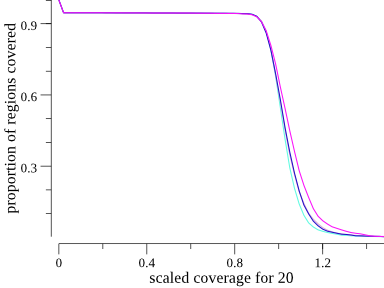

Supplement: Supplementary file 2 — Data file S1 [file sciadv.abp8314_data_file_s1.zip › SHSY5Y-indexcov-roc-20.png]

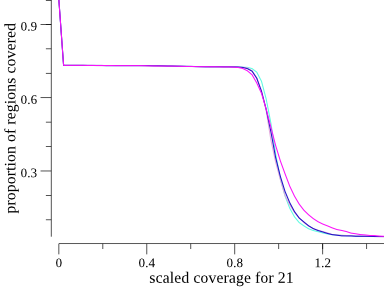

Supplement: Supplementary file 2 — Data file S1 [file sciadv.abp8314_data_file_s1.zip › SHSY5Y-indexcov-roc-21.png]

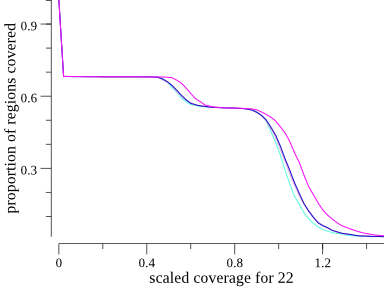

Supplement: Supplementary file 2 — Data file S1 [file sciadv.abp8314_data_file_s1.zip › SHSY5Y-indexcov-roc-22.png]

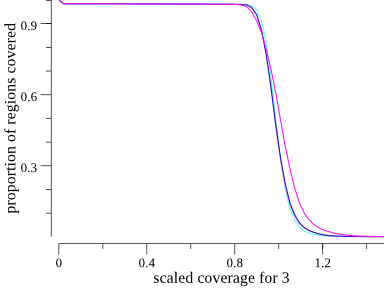

Supplement: Supplementary file 2 — Data file S1 [file sciadv.abp8314_data_file_s1.zip › SHSY5Y-indexcov-roc-3.png]

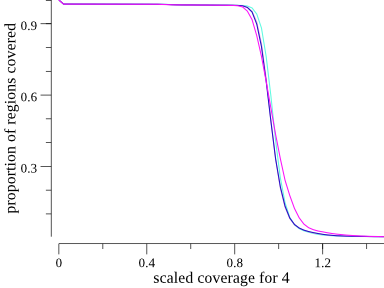

Supplement: Supplementary file 2 — Data file S1 [file sciadv.abp8314_data_file_s1.zip › SHSY5Y-indexcov-roc-4.png]

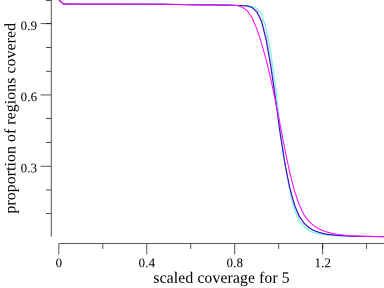

Supplement: Supplementary file 2 — Data file S1 [file sciadv.abp8314_data_file_s1.zip › SHSY5Y-indexcov-roc-5.png]

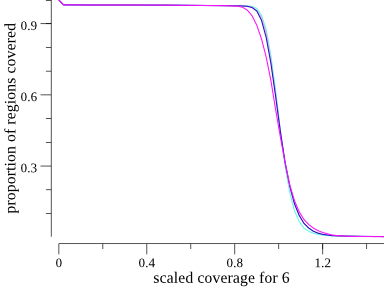

Supplement: Supplementary file 2 — Data file S1 [file sciadv.abp8314_data_file_s1.zip › SHSY5Y-indexcov-roc-6.png]

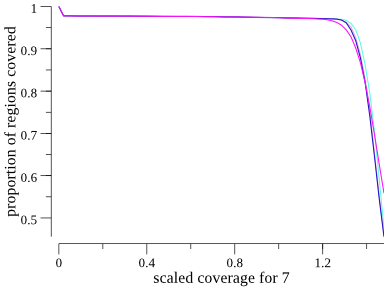

Supplement: Supplementary file 2 — Data file S1 [file sciadv.abp8314_data_file_s1.zip › SHSY5Y-indexcov-roc-7.png]

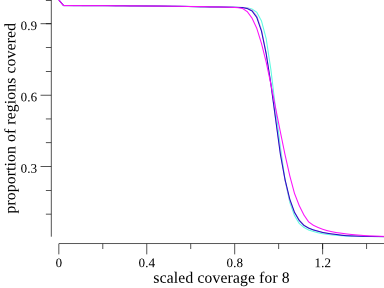

Supplement: Supplementary file 2 — Data file S1 [file sciadv.abp8314_data_file_s1.zip › SHSY5Y-indexcov-roc-8.png]

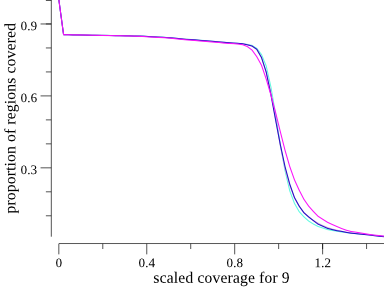

Supplement: Supplementary file 2 — Data file S1 [file sciadv.abp8314_data_file_s1.zip › SHSY5Y-indexcov-roc-9.png]

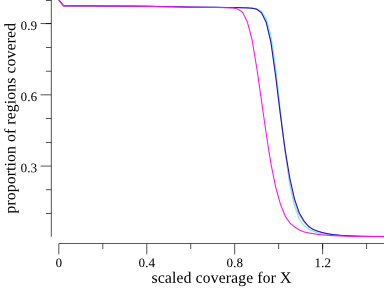

Supplement: Supplementary file 2 — Data file S1 [file sciadv.abp8314_data_file_s1.zip › SHSY5Y-indexcov-roc-X.png]

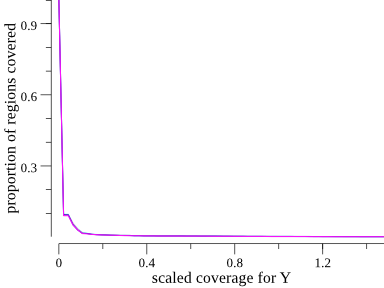

Supplement: Supplementary file 2 — Data file S1 [file sciadv.abp8314_data_file_s1.zip › SHSY5Y-indexcov-roc-Y.png]

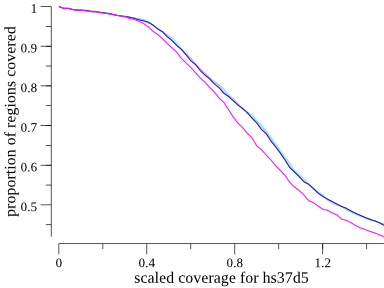

Supplement: Supplementary file 2 — Data file S1 [file sciadv.abp8314_data_file_s1.zip › SHSY5Y-indexcov-roc-hs37d5.png]

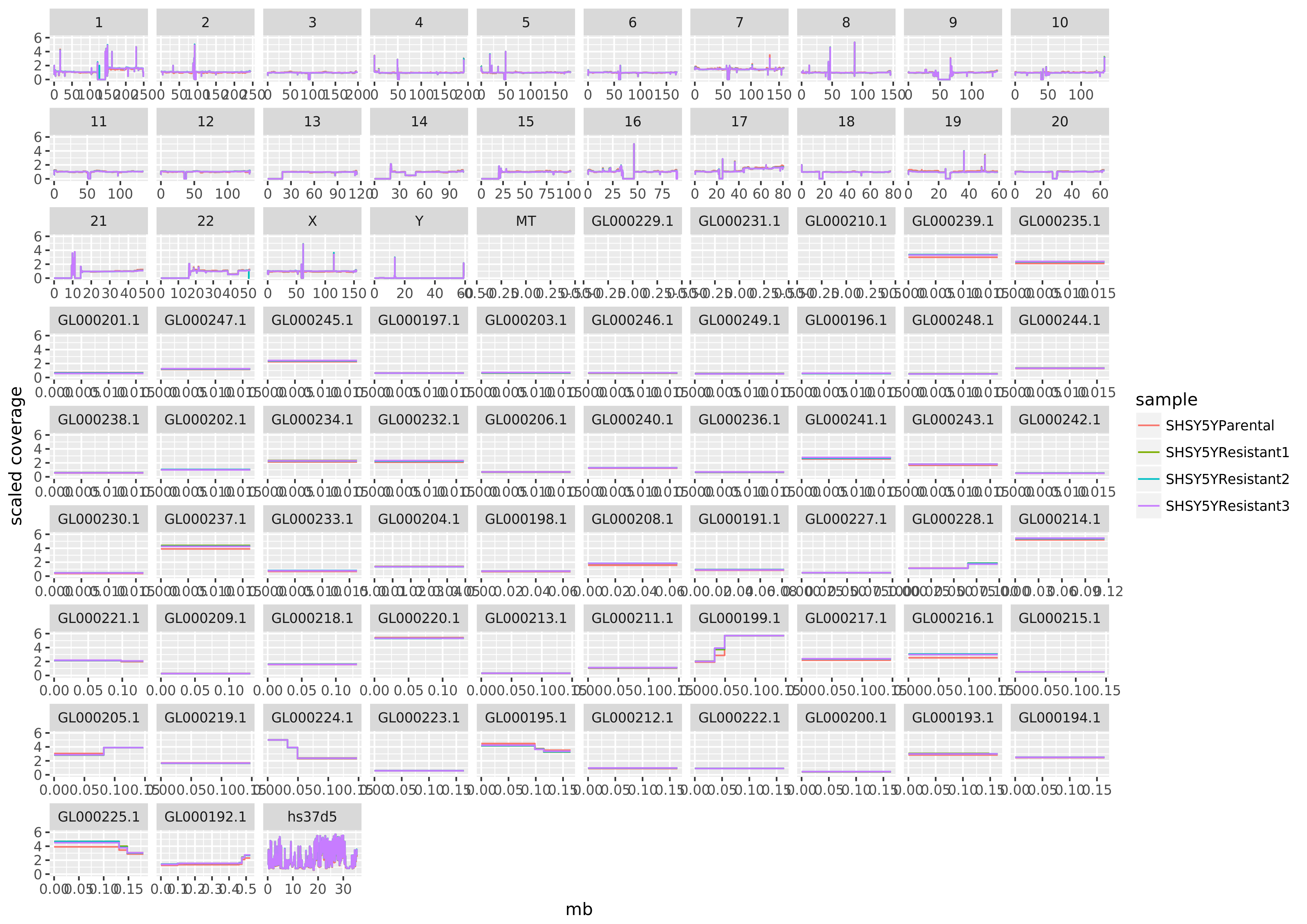

Supplement: Supplementary file 2 — Data file S1 [file sciadv.abp8314_data_file_s1.zip › SHSY5Y-indexcov.smooth.png]
